# Selectively Halogenated Flavonolignans—Preparation and Antibacterial Activity

**DOI:** 10.3390/ijms232315121

**Published:** 2022-12-01

**Authors:** Martina Hurtová, Kristýna Káňová, Simona Dobiasová, Kateřina Holasová, Denisa Čáková, Lan Hoang, David Biedermann, Marek Kuzma, Josef Cvačka, Vladimír Křen, Jitka Viktorová, Kateřina Valentová

**Affiliations:** 1 Institute of Microbiology of the Czech Academy of Sciences, Vídeňská 1083, 142 20 Prague, Czech Republic; 2 Department of Biochemistry and Microbiology, University of Chemistry and Technology Prague, Technická 5, 166 28 Prague, Czech Republic; 3 Institute of Organic Chemistry and Biochemistry of the Czech Academy of Sciences, Flemingovo nám. 542, 160 00 Prague, Czech Republic

**Keywords:** flavonoids, flavonolignans, halogenation, biological activity, multidrug resistance, bacteria

## Abstract

A library of previously unknown halogenated derivatives of flavonolignans (silybins A and B, 2,3-dehydrosilybin, silychristin A, and 2,3-dehydrosilychristin A) was prepared. The effect of halogenation on the biological activity of flavonolignans was investigated. Halogenated derivatives had a significant effect on bacteria. All prepared derivatives inhibited the AI-2 type of bacterial communication (quorum sensing) at concentrations below 10 µM. All prepared compounds also inhibited the adhesion of bacteria (*Staphyloccocus aureus* and *Pseudomonas aeruginosa*) to the surface, preventing biofilm formation. These two effects indicate that the halogenated derivatives are promising antibacterial agents. Moreover, these derivatives acted synergistically with antibiotics and reduced the viability of antibiotic-resistant *S. aureus*. Some flavonolignans were able to reverse the resistant phenotype to a sensitive one, implying that they modulate antibiotic resistance.

## 1. Introduction

Halogen-containing compounds have an outstanding position in medicinal chemistry. Natural products containing halogens are widely distributed in marine organisms and possess numerous interesting biological activities. For example, briarane diterpenoids containing chlorine have been shown to have antibacterial activity [1], hemigerans containing bromine exhibited antifungal activity against *Saccharomyces cerevisiae* [2], and prulonide A (containing both chlorine and bromine) isolated from *Synoicum* showed cytotoxicity against breast cancer cell lines at a concentration of 1 µM [3]. Synthetic halides are commonly used as drugs; e.g., the antimetabolite 5-fluorouracil is used in cancer treatment [4]. Natural products are often altered by synthetic modifications to improve their biological activity. Flavopiridol, a semisynthetic chlorinated flavonoid, is currently undergoing clinical trials as a potent cyclin-dependent kinase 2 inhibitor [5,6].

**Figure 1:**
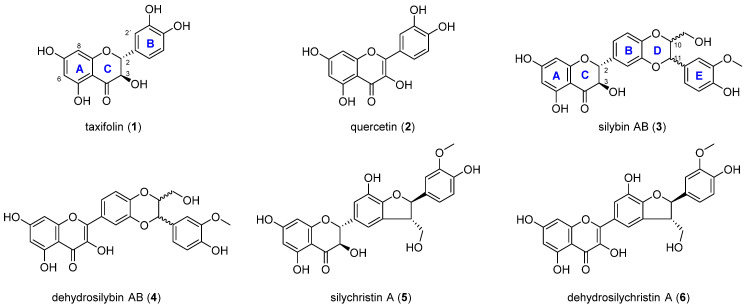
Structure of selected flavonols and flavonolignans.

Flavonoids (e.g., taxifolin (**1**) and quercetin (**2**)) are phenolic secondary metabolites found in plants and fungi. They usually have beneficial biological effects, such as antioxidant, antimutagenic, and anti-inflammatory activities [7]. Flavonolignans form a small subclass of flavonoids, which are mainly isolated from silymarin (extract from the fruits of milk thistle *Silybum marianum*). Silymarin contains mainly silybin AB (**3**), silychristin A (**5**), silydianin, isosilybin AB, and, as minor components, 2,3-dehydrosilybin AB (**4**), 2,3-dehydrosilychristin A (**6**), (Figure 1), taxifolin (**1**), some minor flavonoids, and ca. 30% of a polymeric phenolic fraction [8,9]. These compounds and their derivatives generally have low toxicity and interesting biological activity, such as hepatoprotective activity, anti-inflammatory activity, antioxidant activity, and the ability to modulate the pumps associated with multidrug resistance [8,10,11,12].

The halogenation of flavonoids has been described only for taxifolin and quercetin. Brominated derivatives of quercetin can generally be prepared by the reaction with elemental bromine or with *N*-bromosuccinimide (NBS) [13]. The reaction with elemental bromine leads to a complex mixture of brominated compounds. High regioselectivity of bromination with NBS was achieved only when quercetin was protected at groups 5-OH and 7-OH (methyl, ethyl, isopropyl) [14]. Dibromination of taxifolin was carried out with two equivalents of NBS. Monobrominated taxifolin was prepared by selective debromination of 6,8-dibromotaxifolin with Na_2_SO_3_ and NaHCO_3_ [15].

We have recently developed a new method using α,β-dibromohydrocinnamic acid (DBHCA) for the selective monobromination and dibromination of flavonoids [16]. This method allows substitution exclusively at C-6 in flavonoids saturated at C-2, C-3 (taxifolin (**1**), silybin AB (**3**), silychristin A (**5**)), and C-8 in 2,3-unsaturated flavonoids (quercetin (**2**), 2,3-dehydrosilybin AB (**4**), 2,3-dehydrosilychristin A (**6**)). Here, the bromine is eliminated in situ from DBHCA to form cinnamic acid. Dibromination of flavonolignans at C-6 and C-8 can be achieved at higher temperatures [16].

The chlorination of quercetin at C-6, C-8, and C-3 was previously performed with hypochlorous acid (HOCl) [17]. Freitas et al. described the mono- and dichlorination of quercetin at C-6, C-8, or C-3 with *N*-chlorosuccinimide (NCS) [18]. Iodinated derivatives were prepared with *N*-iodosuccinimide (NIS) on selectively protected quercetin (methyl, ethyl, isopropyl, and benzyl). This method yields an iodinated product at C-6 in the case of protection at group 7-OH. When both the 5-OH and 7-OH groups were protected, iodination occurred at C-8 [19].

Electrophilic fluorination of the A-ring of flavonoids has not yet been described. Fluorinated derivatives of flavones were prepared from fluorinated synthones by various semisynthetic methods, e.g., cyclodehydration of 1-(2-hydroxyphenyl-5-fluoro)-3-aryl-1,3-propanodiones [20] or a reaction of 1-(2-bromo-4-fluorophenyl)-3-phenylprop-2-yn-1-one with benzaldehyde oxime in the presence of a base [21]. In addition, 8-trifluoromethyl-3,5,7,3′,4′-*O*-pentamethyl-quercetin was prepared using Chen’s reagent (methyl fluorosulfonyldifluoroacetate) [22].

Chlorination of quercetin substantially improved anti-inflammatory activity [23] and antioxidant activity [17], compared with that of the parent molecule. Mono- and dibromination of quercetin increased antiviral activity [13] and lipophilicity, which facilitated the transport across the cell membrane [24], while 8-trifluoromethyl-3,5,7,3′,4′-*O*-pentamethyl-quercetin blocked bladder cancer cell growth and promoted apoptotic progression more effectively than quercetin [22]. Unfortunately, some of the reported in vitro studies were performed using mixtures of products, not pure halogenated compounds.

Based on previously published work on the enhancement of biological activity by halogenation, our goal was to prepare a library of novel selectively halogenated derivatives of silymarin flavonolignans that would serve as a platform for further synthetic modifications and basic physicochemical and biological evaluations to identify new lead structures. Since the biological activity of halogenated derivatives of taxifolin (**1**) and quercetin (**2**) has been studied only in mixtures, we prepared their pure brominated derivatives to determine their biological activity. Specifically, the abilities of the halogenated derivatives to inhibit bacterial communication and biofilm formation and to modulate antibiotic resistance in bacteria, as well as their antiradical, reducing, anti-lipoperoxidant, cytotoxic, and anti-inflammatory activities and their ability to modulate doxorubicin-resistant phenotypes in human ovarian carcinoma cells, were evaluated and compared to their parent compounds. 

## 2. Results and Discussion

### 2.1. Chemistry

**Scheme 1:**
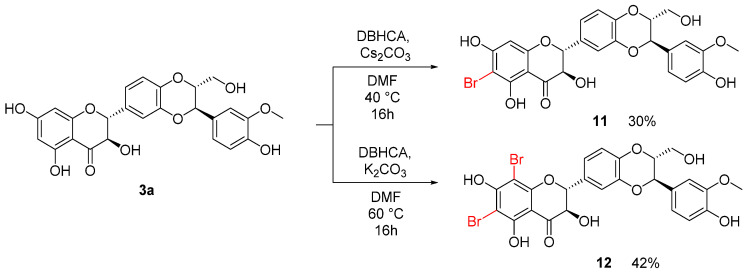
Use of α,β-dibromohydrocinnamic acid (DBHCA) for bromination of silybin A [16]. Bromines are highlighted in red.

Monobrominated derivatives were prepared from the respective flavonoid, employing our original method for selective monobromination using DBHCA in the presence of Cs_2_CO_3_. The selectivity of this reaction was controlled by using different bases, e.g., K_2_CO_3_, and higher temperatures to prepare dibrominated derivatives (Scheme 1) [16].

**Scheme 2:**
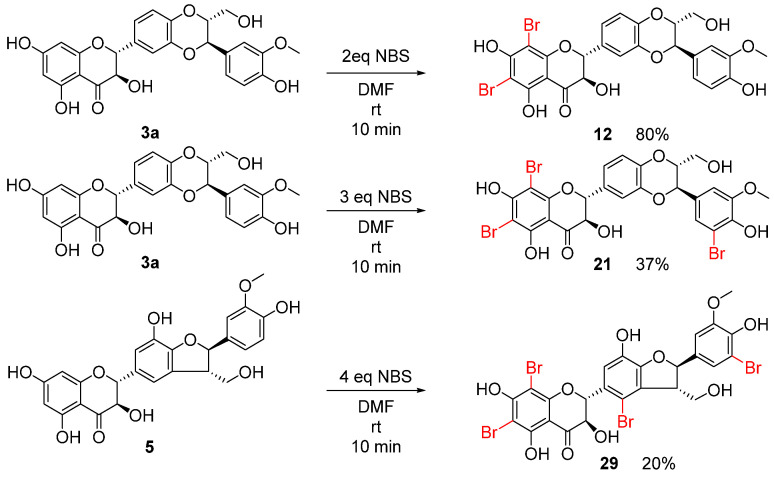
Bromination of silybin A (**3a**) and silychristin A (**5**) using different concentrations of NBS. Bromines are highlighted in red.

The bromination of silybin with one equivalent of NBS afforded the mixture of mono- and dibrominated product and unreacted starting material (ratio 1:1:1, determined by HPLC). The low yield and complicated separation made this reaction impractical. Di- and tribrominated derivatives were prepared by electrophilic substitution with different concentrations of NBS (Scheme 2).

The use of different concentrations of NBS revealed that the most reactive positions for an electrophilic attack in the flavonolignan structure are C-6 and C-8. The use of four equivalents of NBS with silychristin A (**5**) yielded a mixture of 6,8,19-tribromosilychristin A (**23**) and 6,8,12,19-tetrabromosilychristin A (**29**). The use of four equivalents of NBS with silybin A (**3a**) afforded a complex mixture of inseparable polybrominated products. Monobrominated derivatives at C-8 (8-bromosilybin A (**19**) and 8-bromosilybin B (**20**)) were prepared in good yields by selective dehalogenation reaction of 6,8-dibromosilybin A (**12**) and 6,8-dibromosilybin B (**14**) in the presence of Na_2_SO_3_ and NaHCO_3_.

**Scheme 3:**
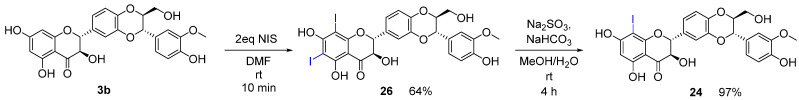
The preparation of iodinated derivatives of silybin B (**24**, **26**). Iodines are highlighted in blue.

Iodinated derivatives of 2,3-saturated flavonoids (taxifolin (**1**), silybin AB (**3**), silychristin A (**5**)) were prepared in good yields using NIS. Diiodinated derivatives were prepared using two equivalents of NIS and 8-iodosilybin B (**24**) was prepared by selective dehalogenation of 6,8-diiodosilybin B (**26**) with Na_2_SO_3_ and NaHCO_3_ in excellent yields (97%) (Scheme 3).

Iodinated 2,3-unsaturated derivatives (quercetin (**2**), 2,3-dehydrosilybin AB (**4**), 2,3-dehydrosilychristin A (**6**)) were highly unstable and their decomposition was observed by NMR analysis, yielding a complex mixture of uncharacterized products. All iodinated derivatives of 2,3-saturated flavonoids exhibited low stability during storage and were decomposed to a mixture of the respective 8-iodo derivative and/or the deiodinated compound.

A mixture of monochlorinated derivatives at C-6 and C-8 of silybin was prepared using one equivalent of NCS. The use of more equivalents of NCS resulted in a mixture of inseparable (poly)chlorinated compounds.

**Figure 2:**
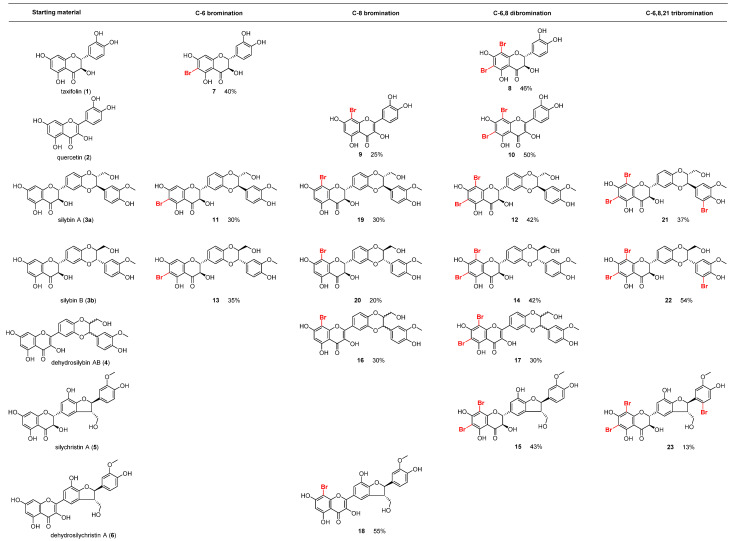
Prepared brominated derivatives of flavonoids. Bromines are highlighted in red.

**Figure 3:**
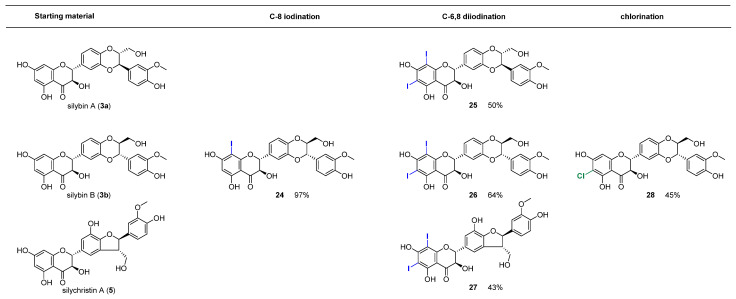
Prepared iodinated and chlorinated derivatives of flavonolignans. Iodinies are highlighted in blue.

All derivatives prepared for the biological tests are summarized in Figure 2 and Figure 3.

**Scheme 4:**
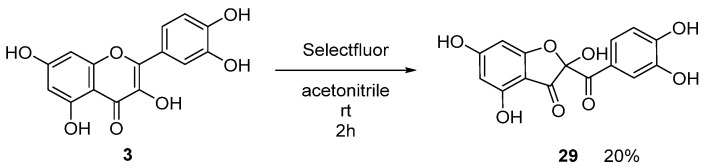
The reaction of quercetin (**2**) with Selectfluor led to the contraction of the C-ring, affording compound **29**.

Fluorination of silybin AB (**3**) with diethylaminosulfur trifluoride (DAST) yielded a mixture of products containing, mainly, the product of oxidation (dehydrogenation) at C-2. The reaction of quercetin with Selectfluor (1-fluoro-4-methyl-1,4-diazoniabicyclo [2.2.2]octane-bis-(tetrafluoroborate)) led to the oxidation of the C-ring, forming a five-membered benzofuranone ring (Scheme 4).

All of the above fluorinating agents are very strong oxidative reagents that are not suitable for use with oxidation-prone compounds such as flavonoids. The formation of the Grignard reagent using Mg with 8-iodo-3,3′,4′,5,7-penta-*O*-isopropylquercetin or 8-bromo-3,3′,4′,5,7-penta-*O*-isopropylquercetin and the reaction with Xtalfluor were not successful. Therefore, the electrophilic fluorination of flavonoids remains a major challenge.

### 2.2. Biological Activity

Recently, we discovered that flavonolignans modulate antibiotic resistance and virulence of *Staphylococcus aureus* by affecting the corresponding efflux pumps [12]. Moderately lipophilic structures are required for this type of biological activity, which involves the interaction with cell membranes and transmembrane proteins [25]. Because bromination increases lipophilicity [24], all prepared compounds were tested for their potential to inhibit bacterial communication (quorum sensing), surface colonization (biofilm formation), and the modulation of antibiotic resistance in both Gram-positive and Gram-negative bacteria.

#### 2.2.1. Inhibition of Bacterial Communication

Bacterial intercellular communication (quorum sensing) is the process by which bacteria determine (sense) the number (quorum) of bacterial cells in their environment. In this cell-to-cell chemical communication, bacteria produce small molecules—autoinducers (AI)—that are secreted into the extracellular environment and, at the same time, determine their quantity in their environment via transmembrane receptors. When autoinducers are present in sufficient concentrations, the signaling pathway is activated, significantly altering the expression profile of bacteria. As a result, bacterial toxins and virulence factors can be produced, or biofilm formation and sporulation may occur [26].

Commercially available strains of *Vibrio campbellii* were used to evaluate the ability of flavonoids and their derivatives to inhibit bacterial quorum sensing. *V. campbellii* uses two types of autoinducing molecules for its communication—*N*-acyl-homoserine lactones (Autoinducer I, AI-1, strain BAA 1118) and furanosyl borate diester (Autoinducer II, AI-2, strain BAA 1119) [27]. AI-1-based communication is mainly used by Gram-negative bacteria, while AI-2-based communication is found in both Gram-negative and Gram-positive bacteria. The mutant strains of *V. campbellii* BA1118 and BA1119 respond only to AI-1 or AI-2, respectively, and the response to the respective AI concentration is measured as luminescence. The higher the AI concentration, the higher the luminescence signal. The ability to inhibit the communication was expressed as the selectivity index (SI), calculated as the ratio between the concentration that halved the viability (IC_50_) and the concentration that halved the communication (EC_50_) of the respective *V. campbellii* strains. The higher the SI, the better the inhibitor, and this allows for better dosing at concentrations that are not themselves antimicrobial. The antimicrobial activity of quorum-sensing inhibitors is undesirable because it creates a selection pressure and can lead to the development of resistance. The measured data of the parent flavonoids and their halogenated derivatives are summarized in Table 1.

AI-1-type communication was inhibited by 2,3-dehydrosilybin AB (**4**) (SI 2.7, EC_50_ = 59.9 µM) and 2,3-dehydrosilychristin A (**6**) (SI 1.9, EC_50_ = 22.3 µM) (Table 1), while 8-bromination significantly improved the activity, especially in the case of silybin A (**19**, SI 3.4), silybin B (**20**, SI 2.9), and 2,3-dehydrosilychristin A (**18**, SI 2.7). Of all the compounds, 6,8-dibromodehydrosilybin AB (**17**, SI > 3.6) was the most promising for inhibiting AI-1, which is consistent with the previously reported inhibition of AI-1-type quorum sensing by several flavonoids [28], along with the hypothesis that halogenation may enhance this ability [29].

AI-2-type communication was significantly inhibited by the parent compounds taxifolin (**1**) (SI 11.0, EC_50_ = 7.3 µM), silybin A (**3a**) (SI 7.0, EC_50_ = 17.1 µM), and silybin B (**3b**) (SI 6.8, EC_50_ = 22.3 µM). The ability of flavonolignans to inhibit AI-2-type communication was significantly increased for all 8-bromo derivatives, namely quercetin (**9**), silybin A (**19**), silybin B (**20**), 2,3-dehydrosilybin AB (**16**), and 2,3-dehydrosilychristin A (**18**), as well as in the cases of 6-chlorosilybin B (**28**), 6,8-dibromoquercetin (**10**), and 6,8,21-tribromosilybin A (**21**). In such cases, halogenation significantly improved the selectivity index.

This work contains the first study of the effects of halogenation on the ability of flavonoids to inhibit bacterial communication. In the case of AI-2-based communication, brominated derivatives inhibited activity with an EC_50_ value below 10 µM, and toxicity against bacteria was very low. Compounds capable of affecting bacterial communication have promising therapeutic potential in the field of regulating bacterial virulence [30]. We also suggest that chlorinated derivatives of flavonoids may be the most promising inhibitors of bacterial communication. Unfortunately, their preparation was not successfully optimized in this work, due to the high reactivity of chlorine in electrophilic substitutions.

#### 2.2.2. Effect on Biofilm Formation

Biofilm formation is a fully organized, multistep process in which bacteria are constantly communicating with each other. We found that most tested flavonoids and their derivatives inhibited bacterial cell-to-cell communication; accordingly, their ability to affect the surface adhesion of Gram-positive (*S. aureus*) and Gram-negative (*Pseudomonas aeruginosa*) bacteria was also investigated.

**Figure 4:**
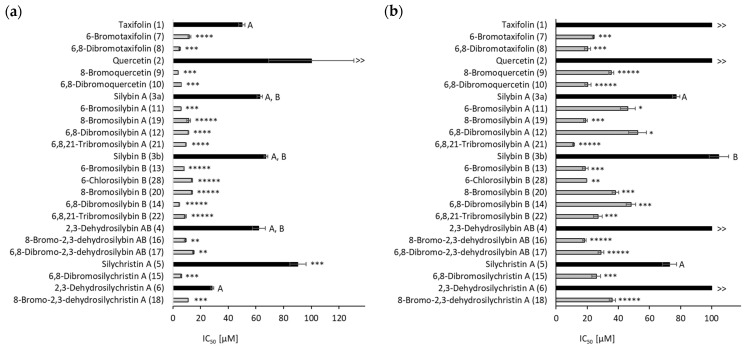
Inhibition of *S. aureus* (ATCC, 25923) (**a**) and *P. aeruginosa* (CCM, 3955) (**b**) surface colonization by flavonoids and their halogenated derivatives. The data are presented as the average of three repetitions with the standard error. A *t*-test was used for the comparison of the halogenated derivative and its parent compound (* *p* ≤ 0.05, ** *p* ≤ 0.01, *** *p* ≤ 0.005, **** *p* ≤ 0.001, ***** *p* ≤ 0.0005). ANOVA with Duncan´s post hoc test was used for the comparison of the parent compounds (different capital letters to the right of the respective bars indicate significant differences).

The inhibition of *P. aeruginosa* biofilm formation was only observed for silybin A (**3a**, IC_50_ = 77 µM), silybin B (**3b**, IC_50_ = 105 µM), and silychristin A (**5**, IC_50_ = 73 µM). Except for quercetin (**2**), all compounds inhibited *S. aureus* biofilm formation, with an IC_50_ value of less than 100 µM. In all cases, halogenation resulted in a significant increase in inhibitory activity, compared with that of the parent compounds with an IC_50_ value mostly below 10 µM (Figure 4).

Disruption of the matured biofilm was not detected by any of the tested flavonoids or their derivatives, up to a concentration of 100 µM.

Flavonoids (myricetin, hesperetin, and phloretin) have previously been reported to inhibit *S. aureus* biofilm formation at concentrations of 4 µM and less [31]. Our work is the first to report the effect of halogenation on the ability of flavonoids to inhibit biofilm formation.

#### 2.2.3. Modulation of Antibiotic-Resistant Phenotype in Resistant Bacteria

The ability of the compounds to modulate the antibiotic-resistant phenotype in resistant bacteria was also investigated. To use flavonoids as modulators of antibiotic resistance, they should not, themselves, possess antimicrobial activity. Multidrug-resistant clinical strains of *P. aeruginosa* and *S. aureus* were incubated with the concentration range of flavonoids and their halogenated derivatives to determine antimicrobial activity. Neither of the tested derivatives exhibited antimicrobial activity. None of the compounds tested were able to halve bacterial growth below a concentration of 100 µM.

Clinically relevant antibiotics were selected for sensitization testing according to the European Committee on Antimicrobial Susceptibility Testing (EUCAST). Neither colistin and imipenem at breakpoint concentrations (according to EUCAST, 4 mg/mL imipenem and 2 mg/mL colistin) affected the growth of the multidrug-resistant clinical strain of *P. aeruginosa*. Similarly, neither chloramphenicol and gentamicin at breakpoint concentrations (8 mg/mL and 1 mg/mL, respectively) affected the multidrug-resistant clinical strain of *S. aureus*. Flavonoids and their derivatives (1–40 µM) were used in combination therapy, along with the breakpoint concentration of antibiotics. After co-incubation, the minimum inhibitory concentration of flavonoids inhibiting the visible growth of bacteria was determined (Table 2).

None of the compounds tested affected the sensitivity of *P. aeruginosa* to imipenem: 8-bromo-2,3-dehydrosilybin AB (**16**) reversed the colistin-resistant phenotype in *P. aeruginosa* to a sensitive one, while 6,8-dibromo-2,3-dehydrosilybin AB (**17**) reduced growth to 47%. The chloramphenicol-resistant phenotype of *S. aureus* was not affected by the flavonoids or halogenated derivatives. Only 2,3-dehydrosilychristin A (**6**, 40 µM) reduced its growth, to 69%. The gentamicin-resistant phenotype of *S. aureus* was reversed by 5 µM 2,3-dehydrosilybin AB (**4**) and the growth of this multidrug-resistant strain was reduced to 37% by the addition of 40 µM taxifolin (**1**).

The 2,3-saturated parent flavonolignans (silybins and silychristin) had no sensitizing potential, but their bromination significantly enhanced this activity in gentamicin-resistant *S. aureus*. Moreover, the presence of an additional bromine atom on the E ring in 6,8,21-tribromosilybin A (**21**) and 6,8,21-tribromosilybin B (**22**) further enhanced this activity and reversed the resistant phenotype to a sensitive one, at 40 µM.

#### 2.2.4. Antioxidant Capacity, Reducing Potential and Lipoperoxidation Inhibition

To further elucidate how halogenation affects other properties of the flavonoids studied, their 2,2′-azino-bis-(3-ethylbenzothiazoline-6-sulfonic acid) cation (ABTS) and 1,1-diphenyl-2-picrylhydrazyl (DPPH) radical scavenging, the Folin–Ciocalteu reagent (FCR) reducing, the ferric-reducing antioxidant power (FRAP), and the anti-lipoperoxidation activities were evaluated by in vitro methods (Supplementary Information Tables S1 and S2). The activity of the parent flavonoids was consistent with previously published data [32,33,34,35,36,37,38], with the 2,3-unsaturated compounds being more active. Halogenation did not affect these activities, with a few exceptions: 6,8-dibromotaxifolin (**8**) showed increased activity in FRAP and FCR assays, compared with taxifolin (**1**); 6,8-dibromoquercetin (**10**) showed much higher activity than both 8-bromoquercetin (**9**) and quercetin (**2**) in the FRAP test; and 8-iodosilybin B (**24**) showed 2.5-fold higher anti-lipoperoxidant activity than silybin B (**3b**). In the case of anti-lipoperoxidation activity, taxifolin (**1**) and 6,8-dibromotaxifolin (**8**) showed the highest inhibitory potential, with higher activity than that of quercetin and Trolox (positive control) (Table S2). Quercetin (**2**), 8-bromoquercetin (**9**), and 6,8-dibromoquercetin (**10**) also showed high potential to inhibit lipid peroxidation, with activity increasing with an increasing number of bromines. The values for the parent compounds can be found in the literature, but the data presented here are new for halogenated flavonolignans. To date, only the DPPH scavenging activity of brominated flavonols has been studied [24]; however, because the authors used crude reaction mixtures and not pure brominated derivatives, their results are not comparable to ours.

#### 2.2.5. Cytotoxicity and Anti-Inflammatory Activity

In addition, the cytotoxicity of these compounds was evaluated as the ability to decrease the viability of human dermal fibroblasts (HDF) and human ovarian carcinoma cells that are resistant to doxorubicin (HOC/DOX) (Table S3). Neither of the tested derivatives exhibited cytotoxicity against healthy or cancer cells. These results are consistent with our previous findings [34,38] that silybin and silychristin had no direct anticancer effect but showed the potential to act as adjuvant therapy to conventional chemotherapeutic agents and to modulate tumor resistance. Moreover, flavonoids and flavonolignans have been a common component of the human diet for centuries and have never shown adverse effects [39]. Halogenation did not improve the ability of flavonoids to modulate the drug-resistant phenotype in doxorubicin-resistant human ovarian carcinomas (Figure S2).

To our knowledge, this is the first report of the effects of bromination on the anti-inflammatory activity of flavonoids, which was evaluated as the ability of flavonoids to decrease nitric oxide production (Tables S4 and S5). The results indicate that nine of the 16 compounds tested were more active than the positive control, indomethacin.

## 3. Materials and Methods

### 3.1. General Experimental Procedures

Procedures using oxygen or moisture-sensitive materials were performed with anhydrous solvents (vide infra) under an atmosphere of argon in flame-dried flasks, using standard Schlenk techniques. Analytical TLC was performed on Al plates (Silica Gel 60 F254; Merck, Darmstadt, Germany). Purification was performed in a preparative HPLC system using an ASAHIPAK GS-310 20F column (Shodex, Munich, Germany), with MeOH as the mobile phase, a flow rate of 5 mL/min, and detection at 254 nm and 369 nm. The preparative HPLC system (Shimadzu, Kyoto, Japan) consisted of an LC-8A high-pressure pump with an SPD-20A dual wavelength detector (with a preparative cell), FRC-10A, and a fraction collector. The system was connected to a PC via a CBM-20A command module and controlled via the LabSolution 1.24 SPI software suite supplied with the instrument. All analytical HPLC separations were performed using a Shimadzu Prominence System (Shimadzu, Kyoto, Japan) consisting of a DGU-20A mobile phase degasser, two LC-20AD high-pressure pumps, a SIL-20AC refrigerated autosampler, a CTO-10AS column oven, and an SPDM20 A diode array detector. Shimadzu Solution software was used to acquire chromatographic data at a rate of 40 Hz. A monolithic Chromolith Performance RP-18e column (100 × 3 mm i.d., Merck, Darmstadt, Germany) was coupled with a guard column (5 × 4.6 mm, Merck, Darmstadt, Germany). Mobile phase A, CH_3_CN/H_2_O/HCOOH (5:95:0.1), and phase B, CH_3_CN/H_2_O/HCOOH (80:20:0.1), were used for the analyses with the following gradient: 0–6 min 10–80% B; 6–8 min 80% B; 10–12 min 80–10% A. The flow rate was 0.4 mL/min at 25 °C. MS parameters were as follows: negative mode; ESI interface voltage, 4.5 kV; detector voltage, 1.15 kV; nebulization gas flow, 1.5 mL/min; drying gas flow, 15 mL/min; heater block temperature, 200 °C; DL temperature, 250 °C; scan mode 300–800 *m*/*z*. PDA data were acquired in the 200 nm to 450 nm range, and the signal at 285 nm was used to monitor separation. Flash column chromatography was performed on Kieselgel 60 (60–200 mesh). NMR analyses were performed using Bruker Avance III 700 MHz (700.13 MHz for ^1^H, 176.05 MHz for ^13^C), Bruker Avance III 600 MHz (600.23 MHz for ^1^H, 150.93 MHz for ^13^C), and Bruker Avance III 400 MHz (399.87 MHz for ^1^H, 100.55 MHz for ^13^C) spectrometers in DMSO-*d*_6_ at 30 °C and in CDCl_3_ at 20 °C. The signals in DMSO-*d*_6_ and CDCl_3_ were used as reference (δ_H_ 2.499, δ_C_ 39.46 for DMSO-*d*_6_ and δ_H_ 7.263, δ_C_ 77.01 for CDCl_3_). Spectra were recorded using the manufacturer’s software. The ^1^H and ^13^C NMR spectra were zero filled to 4-fold data points and multiplied by a window function before Fourier transformation. To improve resolution, a double-exponential Lorentz–Gauss function with two parameters was applied for ^1^H, and line broadening (1 Hz) was applied for ^13^C to obtain a better signal-to-noise ratio. Chemical shifts were reported on the δ-scale, and the digital resolution justified the reported values to three (δ_H_) or two (δ_C_) decimal places.

High-resolution mass spectra (HRMS) were measured using a LTQ Orbitrap XL hybrid mass spectrometer (Thermo Fisher Scientific, Waltham, MA, USA) equipped with an electrospray ion source and operated at the resolution of 100,000. The samples were loop injected into methanol/water (4:1), with a flow rate of 100 μL/min.

Mono- and dibrominated compounds (compounds **7**–**17**) were prepared according to a previously published method for selective bromination [16]. Commercially available reagents, ligands, and pooled microsomes from male rat liver (M9066), 1,1-diphenyl-2-picrylhydrazyl (DPPH) radical, and tert-butyl hydroperoxide (tBH) were purchased from Sigma Aldrich (Darmstadt, Germany), Alfa Aesar (Ward Hill, MA, USA), Acros Organics (Morris Plains, NJ, USA), and TCI chemicals (Gurugram, India) and, unless stated otherwise, were used without further purification. Pure diastereomers of silybin were prepared using diastereomeric enzymatic resolution [40]. The 2,3-dehydrosilybin AB and 2,3-dehydrosilychristin A were prepared as described previously [36,41]. The FRAP (ferric reducing antioxidant power, KF-01-003) and the ABTS (2,2’-azino-bis-(3-ethylbenzothiazoline-6-sulphonic acid, KF-01-002) radical scavenging kits were acquired from Bioquochem (Llanera, Spain). The Folin–Ciocalteu reagent, human dermal fibroblasts (HDF), doxorubicin-resistant human ovarian carcinoma (HOC/DOX), Dulbecco’s Modified Eagle’s Medium, antibiotic antimycotic solution, murine macrophages (RAW 264.7), and Griess reagent were purchased from Merck (Darmstadt, Germany). Fetal bovine serum and MEM medium were purchased from Thermo Fisher Scientific (Waltham, MA, USA). *Vibrio campbellii* BAA-1118 and BAA-1119 strains (American Type Culture Collection, ATCC, Manassas, VA, USA) were used for the detection of bacterial communication inhibition. A SpectraMax i3x microplate reader (Molecular Devices, San Jose, CA, USA) was used for the biological activity measurements. GraphPad Prism software version 5.00 for Windows (GraphPad Software, San Diego, CA, USA) and Statext software ver. 2.1 (STATEXT LLC, Wayne, NJ, USA) were used for the evaluation of the results.

### 3.2. General Procedure A for Tribromination

NBS (3.0 eq) was added to a solution of starting material (1.0 eq) in DMF (2 mL) at room temperature and the mixture was stirred for 30 min. The reaction mixture was poured into water and extracted with ethyl acetate (3 × 10 mL). The combined organic fractions were washed with brine, dried over Na_2_SO_4_, and evaporated in vacuo. The residue was purified by preparative HPLC chromatography (ASAHIPACK, 5 mL/min MeOH isocratic) to afford the corresponding product.

For 6,8,21-tribromosilybin A (**21**), general procedure A was followed and yielded **21** as brown petals (110 mg, yield 37%, HPLC purity 96%—Figure S25); ^1^H and ^13^C NMR data (DMSO-*d*_6_, 30 °C, 700.13 and 176.05 MHz, respectively), Table S7, Figures S7 and S8; HRMS (ESI, *m*/*z*) calcd for C_25_H_18_O_10_Br_3_ [M − H]^−^ 714.84556, found 714.84382, Figure S35.

For 6,8,21-tribromosilybin B (**22**), general procedure A was followed and afforded **22** as a white solid (160 mg, yield 54%, HPLC purity 100%—Figure S26); ^1^H and ^13^C NMR data (DMSO-*d*_6_, 30 °C, 399.87 and 100.55 MHz, respectively), Table S8, Figures S9 and S10; HRMS (ESI, *m*/*z*) calcd for C_25_H_18_O_10_Br_3_ [M − H]^−^ 714.84556, found 714.84485, Figure S36.

For 6,8,20-tribromosilychristin A (**23**), general procedure A was followed and afforded **23** as a white solid (20 mg, yield 13%, HPLC purity 94%—Figure S27); ^1^H and ^13^C NMR data (DMSO-*d*_6_, 30 °C, 600.23 and 150.93 MHz, respectively), Table S9, Figures S11 and S12; HRMS (ESI, *m*/*z*) calcd for C_25_H_18_O_10_Br_3_ [M − H]^−^ 714.84556, found 714.84418, Figure S37.

### 3.3. General Procedure B for Diiodination

NIS (2.0 eq) was added to a solution of the starting material (1.0 eq) in DMF (2 mL) at room temperature and the mixture was stirred for 30 min. The reaction mixture was poured into water and extracted with ethyl acetate (3 × 10 mL). The combined organic fractions were washed with brine, dried over sodium sulfate, and evaporated in vacuo. The residue was purified by preparative HPLC chromatography (ASAHIPACK, 5 mL/min isocratic) to afford the corresponding product.

For 6,8-diiodosilybin A (**25**), general procedure B was followed and yielded **25** as a white solid (30 mg, yield 50%, HPLC purity 96%—Figure S29); ^1^H and ^13^C NMR data (DMSO-*d*_6_, 30 °C, 600.23 and 150.93 MHz, respectively), Table S11, Figures S15 and S16; HRMS (ESI, *m*/*z*) calcd for C_25_H_19_O_10_I_2_ [M − H]^−^ 732.90731, found 732.90678, Figure S39.

For 6,8-diiodosilybin B (**26**), general procedure B was followed and afforded **26** as a white solid (153 mg, yield 64%, HPLC purity 97%—Figure S30); ^1^H and ^13^C NMR data (DMSO-*d*_6_, 30 °C, 600.23 and 150.93 MHz, respectively), Table S12, Figures S17 and S18; HRMS (ESI, *m*/*z*) calcd for C_25_H_19_O_10_I_2_ [M − H]^−^ 732.90731, found 732.90619, Figure S40.

For 6,8-diiodosilychristin A (**27**), Ggeneral procedure B was followed and yielded **27** as a white solid (65 mg, yield 43%, HPLC purity 96%—Figure S31); ^1^H and ^13^C NMR data (DMSO-*d*_6_, 30 °C, 600.23 and 150.93 MHz, respectively), Table S13, Figures S19 and S20; HRMS (ESI, *m*/*z*) calcd for C_25_H_19_O_10_I_2_ [M − H]^−^ 732.90731, found 732.90620, Figure S41.

### 3.4. General Procedure C for Chlorination

NCS (1.0 eq) was added to a solution of starting material (1.0 eq) in DMF (2 mL) at room temperature and the mixture was stirred for 30 min. The reaction mixture was poured into water and extracted with EtOAc (3 × 10 mL). The combined organic fractions were washed with brine, dried over Na_2_SO_4_, and evaporated in vacuo. The residue was purified by preparative HPLC chromatography (ASAHIPACK, 5 mL/min isocratic) to afford the corresponding product.

For 6-chlorosilybin B (**28a**) and 8-chlorosilybin B (**28b**), general procedure C was followed and afforded an inseparable mixture containing 6-chlorosilybin B (66%, **28a**) and 8-chlorosilybin B (25%, **28b**) as a white solid (70 mg, 45%, HPLC chromatogram—Figure S32); ^1^H and ^13^C NMR data (DMSO-*d*_6_, 30 °C, 600.23 and 150.93 MHz, respectively), Table S14, Figures S21 and S22; HRMS (ESI, *m*/*z*) calcd for C_25_H_20_O_10_Cl [M − H]^−^ 515.07505, found 515.07385, Figure S42.

### 3.5. General Procedure D for Selective Dehalogenation

To a solution of dihalogenated compound (1.0 eq) in MeOH (1 mL) was added a solution of NaHCO_3_ (1.0 eq) in water (0.5 mL), followed by dropwise addition of Na_2_SO_3_ (1 eq) in water (0.5 mL). The reaction mixture was stirred at room temperature for 4 h. The reaction mixture was evaporated and the residue was dissolved in EtOAc and extracted with water (3 × 10 mL). The combined organic fractions were washed with brine, dried over Na_2_SO_4_, and evaporated in vacuo. The residue was purified by preparative HPLC chromatography (ASAHIPACK, 5 mL/min isocratic) to give the corresponding product.

For 8-bromosilybin A (**19**), general procedure D was followed and afforded **19** as a white solid (50 mg, yield 30%, HPLC purity 96%—Figure S23); ^1^H and ^13^C NMR data (DMSO-*d*_6_, 30 °C, 600.23 and 150.93 MHz, respectively), Table S5, Figures S3 and S4; HRMS (ESI, *m*/*z*) calcd for C_25_H_20_O_10_Br [M − H]^−^ 559.02453, found 559.02344, Figure S33.

For 8-bromosilybin B (**20**) general procedure D was followed and yielded **20** as a white solid (20 mg, yield 20%, HPLC purity 97%—Figure S24); ^1^H and ^13^C NMR data (DMSO-*d*_6_, 30 °C, 600.23 and 150.93 MHz, respectively), Table S6, Figures S5 and S6; HRMS (ESI, *m*/*z*) calcd for C_25_H_20_O_10_Br [M − H]^−^ 559.02453, found 559.02355, Figure S34.

For 8-iodosilybin A (**24**), general procedure D was followed and yielded **24** as a white solid (112 mg, yield 97%, HPLC purity 92%—Figure S28); ^1^H and ^13^C NMR data (DMSO-*d*_6_, 30 °C, 700.13 and 176.05 MHz, respectively), Table S10, Figures S13 and S14; HRMS (ESI, *m*/*z*) calcd for C_25_H_20_O_10_I [M − H]^−^ 607.01066, found 607.00892, Figure S38.

### 3.6. Inhibition of Quorum Sensing

The inhibition of bacterial extracellular communication was measured using *Vibrio campbellii* strains BAA-1118 and BAA-1119. The experiment was performed according to the methods of Szemerédi et al. [42]. Briefly, 5 × 105 CFU/mL (colony-forming units per milliliter) were seeded in an Autoinducer Bioassay medium (NaCl 17.5 g/L, MgSO_4_ 12.3 g/L, Casamino acids 2 g/L, 10 mL of 1 M potassium phosphate pH 7.0, 10 mL of 0.1 M L-arginine, and 10 mL of glycerol in 970 mL of deionized water) in a 96-well plate. Immediately, the tested compounds were added at the final concentration of 0.2–200 µM and luminescence was recorded for 16 h by a SpectraMax i3x microplate reader set at 30 °C, an integration time of 10,000 ms, and shaking for 60 s before measurement. The effective concentration of compounds halving the luminescence (EC_50_) was determined from the sum of luminescence. At the same time, the viability of the culture was determined by the resazurin assay, which gave the IC_50_ value for each compound. The selectivity index was calculated as the ratio of EC_50_ (the concentration that halves the cell communication) and IC_50_ (the concentration halving the viability). The EC_50_ and IC_50_ were calculated using GraphPad Prism software version 5.00 for Windows with a nonlinear regression curve fit.

### 3.7. Sensitization of Antibiotic-Resistant Bacteria

Clinical isolates of *P. aeruginosa* and *S. aureus* were obtained from the General University Hospital in Prague (Prague, Czech Republic). *P. aeruginosa* was resistant to ciprofloxacin, oxacillin, ticarcillin, colistin, gentamicin, and imipenem. *S. aureus* was resistant to ciprofloxacin, gentamicin, erythromycin, chloramphenicol, clindamycin, oxacillin, cefotaxime, and vancomycin. Bacteria were cultivated in Mueller–Hinton broth (MH broth, Merck). The susceptibility of the bacteria was evaluated by the minimum inhibitory concentrations (MIC). The MIC of derivatives in the presence of the antibiotic cut-off concentration was determined according to ISO 20776-1: 2020. The antibiotic cut-off concentration was chosen according to the European Committee on Antimicrobial Susceptibility Testing (EUCAST, Clinical breakpoints—bacteria (ver. 11.0), 1 January 2021). The 40 µM concentration of the derivatives was chosen as the highest concentration for the tests. Cell viability was determined by measuring absorbance (A590 nm) after 24 h incubation at 37 °C, 150 rpm.

### 3.8. Inhibition of Biofilm Formation and Disruption of Maturated Biofilm

The activity of derivatives on biofilms was tested with *S. aureus* (ATCC, 25923) and *P. aeruginosa* (CCM, 3955), according to the previously described method [43]. The highest tested concentration of derivatives was 100 µM. The activity was evaluated using an IC_50_ comparison between the parent compound and its derivative.

### 3.9. Antioxidant Activity, Reducing Potential, and Lipid Peroxidation Inhibition

The antiradical activity was evaluated by the ability to scavenge ABTS and DPPH radicals [33,44,45]. Reducing capacity was assessed by the ability to reduce the Folin–Ciocalteu reagent (FCR) and ferric ions (FRAP) [36,46]. The ability to inhibit lipid peroxidation of pooled male rat microsomal liver membranes induced by tert-butyl hydroperoxide was also evaluated and the results were expressed as IC_50_ values [36,47].

### 3.10. Cytotoxicity

The cytotoxicity of flavonoids and their halogenated derivatives was determined as their ability to decrease the viability of human dermal fibroblasts (HDF) and doxorubicin-resistant human ovarian carcinomas (HOC/DOX). Both cell lines were cultivated in Dulbecco’s Modified Eagle’s Medium supplemented with fetal bovine serum (FBS, 10% *v*/*v*) and 1 × antibiotic antimycotic solution and incubated at 37 °C in the atmosphere of 5% CO2. The experiment was performed according to a previously published method [48]. Briefly, 1 × 10^5^ cells/mL were seeded into a 96-well plate (100 µL/well). After 24 h, flavonoids and their derivatives were administered at the concentration range of 31.25–500 µM. After 72 h, the viability was determined by resazurin assay (0.03 mg/mL of resazurin in PBS), where the fluorescence was measured after 2 h of incubation by the SpectraMax i3x microplate reader at a wavelength of 560/590 nm (excitation/emission). The concentration inhibiting half of the population (IC_50_) was determined using nonlinear regression in GraphPad Prism software. The selectivity index was calculated as the ratio between the IC_50_ for HDF (control, non-cancerous cells) and the IC_50_ for HOC/DOX (cancer cells).

### 3.11. Inhibition of Nitric Oxide Production

The anti-inflammatory activity of the tested flavonolignans was determined as the ability to reduce nitric oxide production by murine macrophages (RAW 264.7) stimulated by bacterial lipopolysaccharides, as previously described [34]. Briefly, RAW 264.7 cells were cultivated in DMEM medium supplemented with 10% FBS and 1 × antibiotic antimycotic solution. For the experiment, 1 × 10^6^ cells/mL was seeded into the 96-well plate (100 µL/well). After 48 h, LPS (100 ng/mL) and the samples (6.25–100 µM) were added to MEM medium (Eagle’s Minimum Essential Media, no phenol red). After 24 h, the medium was mixed with Griess reagent (0.04 g/mL), prepared freshly in deionized water at the ratio of 1:1. The absorbance was measured after 15 min at 540 nm by the SpectraMax i3x microplate reader. Cell viability was determined by resazurin assay.

### 3.12. Sensitization of Doxorubicin-Resistant Human Ovarian Carcinoma Cells

The sensitization of doxorubicin-resistant human ovarian cancer cells (HOC/DOX) was performed, as previously described [38]. Briefly, the HOC/DOX cells were cultivated and seeded as described above. The concentration of doxorubicin that halved the viability (IC_50_) of HOC/DOX was determined after 72 h incubation, using the resazurin assay, and then divided by the IC_50_ of doxorubicin in the presence of the tested compound (10 µM). This ratio is referred to as the sensitization fold (SF). The compound was able to sensitize HOC/DOX to doxorubicin when SF > 1. The higher the SF, the better the sensitizing agent.

### 3.13. Statistical Analysis

Data from the biological activity tests were analyzed using the statistical package Statext ver. 2.1 ANOVA, Scheffé, and least-square difference tests for post hoc comparisons between pairs of means. Data are presented as the average number of replicates (n) with the standard error (SE). A *t*-test was used for the comparison between the halogenated derivative and its parent compound (* *p* ≤ 0.05, ** *p* ≤ 0.01, *** *p* ≤ 0.005, **** *p* ≤ 0.001, ***** *p* ≤ 0.0005). Differences were considered statistically significant when *p* < 0.05.

## 4. Conclusions

In conclusion, a library of new halogenated derivatives of flavonolignans was prepared. To understand how halogenation affects biological activity, all prepared derivatives were tested for their basic physicochemical properties and biological activity. Brominated derivatives of flavonoids were able to inhibit bacterial communication (quorum sensing) and biofilm formation with an IC_50_ below 10 µM. Halogenated derivatives were also able to inhibit the growth of gentamicin-resistant *S. aureus*, and 6,8,21-tribromosilybins A (**21**) and B (**22**) were able to revert the resistant phenotype into a sensitive one. In vitro results indicated that brominated derivatives are promising antibacterial derivatives, but further toxicological and pharmacological tests need to be performed. The prepared derivatives showed higher anti-inflammatory activity than the positive control. Possible mechanisms of action in biological assays are under further investigation. To our knowledge, this is the first paper dealing with the preparation and biological activity of brominated flavonolignans.

## Figures and Tables

**Table 1:** The ability of flavonoids and their derivatives to inhibit communication was expressed as the selectivity index (SI), calculated as the ratio between the concentration that halved the viability (IC_50_, µM) and the concentration that halved the communication (EC_50_, µM) of *V. campbellii* BA1118 and BA1119 strains that respond only to autoinducer 1 (AI-1) or AI-2, respectively.

	*Vibrio campbellii* BAA 1118 (AI-1)				*Vibrio campbellii* BAA 1119 (AI-2)			
	EC_50_ [µM]	IC_50_ [µM]	SI	SA ^$^	EC_50_ [µM]	IC_50_ [µM]	SI	SA ^$^
Taxifolin (**1**)	63.7 ± 2.7	71.0 ± 3.0	1.1 ± 0.1	E	7.3 ± 0.3	80.0 ± 1.7	11.0 ± 0.6	A
6-Bromotaxifolin (**7**)	38.9 ± 1.6	59.0 ± 0.1	1.5 ± 0.1	*	11.9 ± 0.5	19.7 ± 1.0	1.7 ± 0.2	***
6,8-Dibromotaxifolin (**8**)	38.1 ± 2.0	55.5 ± 1.0	1.5 ± 0.1	*	10.1 ± 0.4	109.3 ± 0.8	10.8 ± 0.6	
Quercetin (**2**)	15.9 ± 0.5	24.5 ± 0.8	1.5 ± 0.1	C, D	4.8 ± 0.2	10.6 ± 0.5	2.2 ± 0.2	C
8-Bromoquercetin (**9**)	25.6 ± 0.5	27.8 ± 0.5	1.08 ± 0.04	*****	0.4 ± 0.02	1.1 ± 0.1	2.9 ± 0.3	*****
6,8-Dibromoquercetin (**10**)	7.3 ± 0.4	13.8 ± 0.5	1.9 ± 0.2	**	0.7 ± 0.02	3.5 ± 0.1	5.0 ± 0.3	**
Silybin A (**3a**)	97.1 ± 1.0	131.0 ± 3.7	1.3 ± 0.1	D	17.1 ± 0.5	119.6 ± 3.1	7.0 ± 0.4	B
6-Bromosilybin A (**11**)	24.4 ± 0.7	51.7 ± 0.7	2.1 ± 0.1	*****	9.4 ± 0.5	51.5 ± 0.6	5.5 ± 0.3	*
8-Bromosilybin A (**19**)	33.9 ± 1.8	114.9 ± 5.4	3.4 ± 0.3	***	4.6 ± 0.3	>200	≥43.1 ± 2.6	***
6,8-Dibromosilybin A (**12**)	12.2 ± 0.4	23.5 ± 1.0	1.9 ± 0.2	***	13.2 ± 0.3	80.5 ± 4.8	6.1 ± 0.5	
6,8,21-Tribromosilybin A (**21**)	>200	>200			30.7 ± 1.6	>200	≥6.5 ± 0.3	
Silybin B (**3b**)	121.5 ± 0.6	116.6 ± 0.9	0.96 ± 0.01	E	22.3 ± 0.5	152.4 ± 2.4	6.8 ± 0.2	B
6-Bromosilybin B (**13**)	14.5 ± 0.5	16.1 ± 0.1	1.11 ± 0.04	*	26.1 ± 1.3	52.7 ± 0.6	2.0 ± 0.1	*****
6-Chlorosilybin B (**28**)	114.2 ± 5.0	130.1 ± 5.9	1.1 ± 0.1		3.2 ± 0.2	>200	≥62.6 ± 3.6	***
8-Bromosilybin B (**20**)	44.2 ± 0.7	126.6 ± 4.0	2.9 ± 0.1	***	4.7 ± 0.2	200	≥42.9 ± 2.1	***
6,8-Dibromosilybin B (**14**)	14.7 ± 0.7	16.6 ± 0.8	1.1 ± 0.1		19.3 ± 0.5	60.2 ± 3.4	3.1 ± 0.3	*****
6,8,21-Tribromosilybin B (**22**)	4.3 ± 0.3	7.3 ± 0.1	1.7 ± 0.1	*	12.0 ± 0.5	34.4 ± 1.8	2.9 ± 0.3	****
2,3-Dehydrosilybin AB (**4**)	59.9 ± 2.0	163.2 ± 3.8	2.7 ± 0.2	A	10.2 ± 0.4	25.3 ± 1.0	2.5 ± 0.2	C
8-Bromo-2,3-dehydrosilybin AB (**16**)	>200	>200			6.9 ± 0.3	87.0 ± 2.5	12.7 ± 1.0	*****
6,8-Dibromo-2,3-dehydrosilybin AB (**17**)	55.5 ± 1.6	>200	≥3.6 ± 0.1	***	4.1 ± 0.2	126.8 ± 7.4	31.2 ± 3.5	****
Silychristin A (**5**)	>200	>200			>200	>200		
6,8-Dibromosilychristin A (**15**)	>200	>200			>200	>200		
2,3-dehydrosilychristin A (**6**)	22.3 ± 1.0	43.0 ± 1.5	1.9 ± 0.2	B	11.1 ± 0.1	11.0 ± 0.4	0.99 ± 0.05	D
8-Bromo-2,3-dehydrosilychristin A (**18**)	29.4 ± 0.7	80.1 ± 1.7	2.7 ± 0.1	**	10.5 ± 0.2	23.0 ± 0.2	2.2 ± 0.1	*****
5-Fluorouracil (PC)	(0.39 ± 0.05) × 10^−3^	(0.68 ± 0.01) × 10^−3^	1.73 ± 0.05	B, C	(0.44 ± 0.03) × 10^−3^	(3.41 ± 0.03) × 10^−3^	7.8 ± 0.5	B

The data are presented as the average of three repetitions with the standard error. **^$^** Statistical analysis (SA): *t*-test was used for the comparison of the halogenated derivative and its parent compound (* *p* ≤ 0.05, ** *p* ≤ 0.01, *** *p* ≤ 0.005, **** *p* ≤ 0.001, ***** *p* ≤ 0.0005). ANOVA with Duncan’s post hoc test was used for the comparison of parent compounds and positive control (PC) 5-fluorouracil (different capital letters indicate significant differences).

**Table 2:** Viability of multidrug-resistant *P. aeruginosa* and *S. aureus* cultivated in the combination of breakpoint antibiotic concentration (according to EUCAST) and flavonoid (40 µM, unless otherwise stated).

	*Pseudomonas aeruginosa*		*Staphylococcus aureus*	
	Imipenem	Colistin	Chloramphenicol	Gentamicin
	4 mg/L	2 mg/L	8 mg/L	1 mg/L
**Taxifolin (1)**	-	-	-	37 ± 1%
6-Bromotaxifolin (**7**)	-	-	-	46 ± 4%
6,8-Dibromotaxifolin (**8**)	-	-	-	54 ± 5%
**Quercetin (2)**	-	-	-	-
8-Bromoquercetin (**9**)	-	-	-	-
6,8-Dibromoquercetin (**10**)	-	-	-	-
**Silybin A** (**3a**)	-	-	-	-
6-Bromosilybin A (**11**)	-	-	-	37 ± 1%
8-Bromosilybin A (**19**)	-	-	-	41 ± 3%
6,8-Dibromosilybin A (**12**)	-	-	-	40 ± 1%
6,8,21-Tribromosilybin A (**21**)	-	-	-	reversion of resistance
**Silybin B** (**3b**)	-	-	-	-
6-Bromosilybin B (**13**)	-	-	-	67 ± 4%
6-Chlorosilybin B (**28**)	-	-	-	34 ± 2%
8-Bromosilybin B (**20**)	-	-	-	48 ± 9%
6,8-Dibromosilybin B (**14**)	-	-	-	41 ± 1%
6,8,21-Tribromosilybin B (**22**)	-	-	-	reversion of resistance
**2,3-Dehydrosilybin AB** (**4**)	-	-	-	reversion of resistance [5 µM]
8-Bromo-2,3-dehydrosilybin AB (**16**)	-	reversion of resistance	-	-
6,8-Dibromo-2,3-dehydrosilybin AB (**17**)	-	47 ± 3%	-	-
**Silychristin A** (**5**)	-	-	-	-
6,8-Dibromosilychristin A (**15**)	-	-	-	viability 63 ± 3%
**2,3-Dehydrosilychristin A** (**6**)	-	-	69 ± 4% [10 µM]	-
8-Bromo-2,3-dehydrosilychristin A (**18**)	-	-	-	-
**Sulbactam** (**PC**)	-	-	reversion of resistance	-

The data are presented as the average of three repetitions with the standard error. Neither antibiotic nor flavonoid affects the cell viability if applied separately. “-“ indicates that no effect (100% viability) was observed up to the highest flavonolignan concentration tested (>40 µM); “reversion of resistance“ indicates that the compound reversed the resistant phenotype to a sensitive one; “PC“ positive control.

## Data Availability

Not applicable.

## Supplementary Materials

The following supporting information can be downloaded at: https://www.mdpi.com/article/10.3390/ijms232315121/s1.
