# Supplementary material for: Selectively Halogenated Flavonolignans—Preparation and Antibacterial Activity
Source: Int J Mol Sci. 2022 Dec 1;23(23):15121. doi: 10.3390/ijms232315121 (PMC9738062; doi:10.3390/ijms232315121)
Supplement: Supplementary file 1 [file ijms-23-15121-s001.zip › ijms-2053981-supplementary.pdf]

Supplementary Materials

Biological Activity

Table S1. The antiradical and reducing capacity of flavonoids and their halogenated derivatives.

Compound	ABTS [CEAC][a]		FCR [GAE][b]		FRAP [Fe^{2+}eq][c]		DPPH (IC50) [μM][d]	
**Taxifolin (1)**	1.08 ± 0.02	B	1.92 ± 0.05	D	1.45 ± 0.07	F	2.91 ± 0.19	A
6-Bromotaxifolin (**7**)	0.97 ± 0.03	**	1.70 ± 0.01	***	1.33 ± 0.03		7.77 ± 0.35	*****
6,8-Dibromotaxifolin (**8**)	1.04 ± 0.05		2.08 ± 0.04	*	2.20 ± 0.03	*****	4.37 ± 0.22	***
**Quercetin (2)**	2.87 ± 0.23	E	2.33 ± 0.05	E	2.84 ± 0.02	G	2.06 ± 0.07	A
8-Bromoquercetin (**9**)	2.92 ± 0.28		2.01 ± 0.06	****	2.44 ± 0.06	*****	2.44 ± 0.09	*
6,8-Dibromoquercetin (**10**)	2.63 ± 0.19		1.70 ± 0.02	*****	3.31 ± 0.14	**	3.59 ± 0.22	***
**Silybin A (3a)**	0.50 ± 0.03	A	1.35 ± 0.06	B	0.071 ± 0.003	A	321.8 ± 11.5	E
6-Bromosilybin A (**11**)	0.60 ± 0.04		1.06 ± 0.04	****	0.072 ± 0.002		472.1 ± 43.1	
8-Bromosilybin A (**19**)	0.68 ± 0.03	***	1.01 ± 0.02	*****	0.057 ± 0.002	***	> 500	
6,8-Dibromosilybin A (**12**)	0.30 ± 0.04	***	0.85 ± 0.03	*****	0.084 ± 0.004	*	383.1 ± 17.3	*
6,8-Diiodosilybin A (**25**)	0.41 ± 0.01	*	0.95 ± 0.01	*****	0.053 ± 0.003	*****	> 500	
6,8,21-Tribromosilybin A (**21**)	0.12 ± 0.02	*****	0.99 ± 0.02	*****	0.079 ± 0.002		375.2 ± 32.6	
**Silybin B (3b)**	0.55 ± 0.05	A	1.39 ± 0.08	B	0.078 ± 0.004	A	259.1 ± 8.6	D
6-Bromosilybin B (**13**)	0.75 ± 0.02	***	1.17 ± 0.04	*	0.063 ± 0.001	***	354.4 ± 20.1	*
6-Chlorosilybin B (**28**)	0.71 ± 0.02	**	1.25 ± 0.01		0.050 ± 0.002	*****	> 500	
8-Bromosilybin B (**20**)	0.47 ± 0.01		1.10 ± 0.02	**	0.056 ± 0.002	*****	> 500	
8-Iodosilybin B (**24**)	0.47 ± 0.04		1.01 ± 0.02	***	0.079 ± 0.002		97.1 ± 1.2	***
6,8-Dibromosilybin B (**14**)	0.35 ± 0.04	**	1.10 ± 0.05	**	0.081 ± 0.004		255.9 ± 8.9	
6,8-Diiodosilybin B (**26**)	0.48 ± 0.01		0.84 ± 0.04	*****	0.048 ± 0.001	*****	249.3 ± 18.6	
6,8,21-Tribromosilybin B (**22**)	0.17 ± 0.01	*****	1.01 ± 0.04	*	0.062 ± 0.002	****	237.5 ± 3.2	
**2,3-Dehydrosilybin AB (4)**	1.46 ± 0.08	C	1.68 ± 0.03	C	0.72 ± 0.03	C	14.4 ± 0.2	B
8-Bromo-2,3-dehydrosilybin AB (**16**)	1.15 ± 0.03	**	1.56 ± 0.03	***	0.19 ± 0.01	*****	13.4 ± 0.1	*
6,8-Dibromo-2,3-dehydrosilybin AB (**17**)	1.49 ± 0.04		1.50 ± 0.02	*****	0.54 ± 0.04	***	11.7 ± 0.5	*
**Silychristin A (5)**	1.73 ± 0.02	C	1.87 ± 0.07	D	0.25 ± 0.02	B	47.2 ± 1.0	C
6,8-Dibromosilychristin A (**15**)	1.28 ± 0.02	*****	2.03 ± 0.05		0.34 ± 0.03	*	106.2 ± 5.8	**
6,8-Diiodosilychristin A (**27**)	1.13 ± 0.01	*****	1.79 ± 0.05		0.21 ± 0.01		168.0 ± 12.3	**
6,8,20-Tribromosilychristin A (**23**)	1.16 ± 0.04	*	1.57 ± 0.04	***	0.28 ± 0.02		164.0 ± 2.2	*****
**2,3-Dehydrosilychristin A (6)**	2.10 ± 0.06	D	2.43 ± 0.02	E	1.16 ± 0.04	E	10.8 ± 0.4	AB
8-Bromo-2,3-dehydrosilychristin A (**18**)	1.41 ± 0.03	*****	2.22 ± 0.01	*****	0.90 ± 0.08	*	11.1 ± 0.6	
**Positive control (PC)**	1.01 ± 0.01[e]	B	1.00 ± 0.02[f]	A	1.01 ± 0.01[g]	D	3.5 ± 0.1[h]	AB

Data are expressed as mean ± standard error of at least three independently performed measurements performed in triplicates. [a] 2,2′-Azino-bis-(3-ethylbenzothiazoline-6-sulfonic acid) cation radical scavenging (vitamin C equivalent antioxidant capacity); [b] Folin-Ciocalteu reagent reduction (gallic acid equivalents); [c] ferric reducing antioxidant power (Fe^{2+} equivalents); [d] 1,1-diphenyl-2-picrylhydrazyl radical scavenging; [e] vitamin C; [f] gallic acid; [g] Fe^{2+}; [h] Trolox. ANOVA with Duncan´s post hoc test was used for the comparison of parent compounds and positive control for each essay independently (different capital letters indicate significant differences). T-test was used for the comparison of halogenated derivatives and their parent compounds (* $p \leq 0.05$, ** $p \leq 0.01$, *** $p \leq 0.005$, **** $p \leq 0.001$, ***** $p \leq 0.0005$).

Table S2. The capacity of flavonoids and their halogenated derivatives to inhibit lipid peroxidation.

**Compound**	**ILP IC50 [μM][a]**	
**Taxifolin (1)**	16.3 ± 1.1	A
6-Bromotaxifolin (**7**)	200.6 ± 8.4	*****
6,8-Dibromotaxifolin (**8**)	22.5 ± 1.0	*****
**Quercetin (2)**	39.0 ± 2.9	B, C
8-Bromoquercetin (**9**)	38.3 ± 1.9	
6,8-Dibromoquercetin (**10**)	28.6 ± 1.1	***
**Silybin A (3a)**	213.3 ± 11.3	E
6-Bromosilybin A (**11**)	448.6 ± 31.0	*****
6,8-Dibromosilybin A (**12**)	710.3 ± 37.8	*****
6,8,21-Tribromosilybin A (**21**)	212.7 ± 15.0	
**Silybin B (3b)**	291.2 ± 8.7	F
6-Bromosilybin B (**13**)	727.8 ± 57.5	*****
6,8-Dibromosilybin B (**14**)	378.4 ± 25.4	**
6,8,21-Tribromosilybin B (**22**)	255.2 ± 17.8	
**2,3-Dehydrosilybin AB (4)**	34.6 ± 2.3	B
8-Bromo-2,3-dehydrosilybin AB (**16**)	49.2 ± 1.9	*****
6,8-Dibromo-2,3-dehydrosilybin AB (**17**)	56.7 ± 1.6	*****
**Silychristin A (5)**	128.8 ± 4.6	D
6,8-Dibromosilychristin A (**15**)	459.1 ± 33.9	*****
6,8,20-Tribromosilychristin A (**23**)	324.1 ± 22.6	*****
6,8-Diiodosilychristin A (**27**)	97.2 ± 3.5	*****
**2,3-Dehydrosilychristin A (6)**	51.9 ± 2.2	C
8-Bromo-2,3-dehydrosilychristin A (**18**)	60.1 ± 2.7	*
**Trolox (PC)**	32.7 ± 2.4	B

Data are presented as mean ± standard error of at least three independently repeated measurements in triplicates. [a] Inhibition of lipid peroxidation of male rat microsomes induced by tert-butyl hydroperoxide. ANOVA with Duncan´s post hoc test was used for the comparison of parent compounds and positive control (PC, different capital letters indicate significant differences). T-test was used for the comparison of the halogenated derivative and its parent compound (* $p \leq 0.05$, ** $p \leq 0.01$, *** $p \leq 0.005$, **** $p \leq 0.001$, ***** $p \leq 0.0005$). PC—positive control.

Table S3. Cytotoxicity of halogenated flavonoids. Cytotoxicity is defined as the concentration of flavonoids and their halogenated derivatives halving the viability (IC50, μM) of human dermal fibroblasts (HDF) and doxorubicin-resistant human ovarian carcinoma (HOC/DOX).

Compound	HDF IC50 [μM]		HOC/DOX IC50 [μM]		SI
**Taxifolin (1)**	93.8 ± 3.7	C, D	183.2 ± 17.2	C	0.51 ± 0.07
6-Bromotaxifolin (**7**)	122.2 ± 4.1	***	347.1 ± 26.8	*	0.35 ± 0.04
6,8-Dibromotaxifolin (**8**)	157.8 ± 1.6	****	233.2 ± 7.4		0.68 ± 0.03
**Quercetin (2)**	54.8 ± 2.7	B	182.3 ± 1.1	C	0.30 ± 0.02
8-Bromoquercetin (**9**)	131.5 ± 12.7	*	171.3 ± 1.2	***	0.77 ± 0.08
6,8-Dibromoquercetin (**10**)	55.0 ± 1.9		54.2 ± 0.3	*****	1.02 ± 0.04
**Silybin A (3a)**	108.6 ± 4.6	D, E	236.5 ± 10.6	D	0.46 ± 0.04
6-Bromosilybin A (**11**)	95.6 ± 3.0		185.6 ± 4.6	*	0.51 ± 0.03
8-Bromosilybin A (**19**)	77.4 ± 1.1	*	202.1 ± 1.1	*	0.38 ± 0.01
6,8-Dibromosilybin A (**12**)	102.2 ± 3.0		303.9 ± 27.1		0.34 ± 0.04
6,8,21-Tribromosilybin A (**21**)	75.0 ± 2.4	**	200.6 ± 0.2	*	0.37 ± 0.01
**Silybin B (3b)**	83.3 ± 5.4	C	202.4 ± 0.4	C	0.41 ± 0.03
6-Bromosilybin B (**13**)	76.4 ± 1.1		161.8 ± 3.8	**	0.47 ± 0.02
6-Chlorosilybin B (**28**)	146.7 ± 2.4	*****	272.4 ± 5.5	****	0.54 ± 0.02
8-Bromosilybin B (**20**)	97.9 ± 4.4		226.6 ± 9.2		0.43 ± 0.04
6,8-Dibromosilybin B (**14**)	127.6 ± 1.8	***	231.0 ± 1.7	***	0.55 ± 0.01
6,8,21-Tribromosilybin B (**22**)	85.8 ± 1.1		183.1 ± 9.4		0.47 ± 0.03
**2,3-Dehydrosilybin AB (4)**	16.8 ± 0,5	A	23.5 ± 1.5	A	0.71 ± 0.07
8-Bromo-2,3-dehydrosilybin AB (**16**)	16.9 ± 0.6		39.4 ± 3.4	*	0.43 ± 0.05
6,8-Dibromo-2,3-dehydrosilybin AB (**17**)	41.7 ± 1.0	*****	91.3 ± 1.6	*****	0.46 ± 0.02
**Silychristin A (5)**	114.9 ± 11.0	E	204.7 ± 15.6	C	0.56 ± 0.10
6,8-Dibromosilychristin A (**15**)	175.5 ± 0.4	*	248.8 ± 12.5		0.71 ± 0.04
**2,3-Dehydrosilychristin A (6)**	44.2 ± 2.1	B	75.7 ± 8.6	B	0.58 ± 0.09
8-Bromo-2,3-dehydrosilychristin A (**18**)	66.7 ± 5.6	*	208.6 ± 1.8	***	0.32 ± 0.03
**Indomethacin (PC)**	221.6 ± 13.3	F	17.1 ± 0.6	A	12.94 ± 1.26

The data are presented as the average of four repetitions with the standard error. T-test was used for the comparison of the halogenated derivative and its parent compound (* $p \leq 0.05$, ** $p \leq 0.01$, *** $p \leq 0.005$, **** $p \leq 0.001$, ***** $p \leq 0.0005$). ANOVA with Duncan´s post hoc test was used for the comparison of parent compound toxicities for each cell line independently (different capital letters indicate significant differences). The selectivity index (SI) represents the ratio between toxicity and anticancer activity (HDF and HOC/DOX). PC – positive control.

Table S4. Anti-inflammatory activity of flavonoids and their halogenated derivatives. The anti-inflammatory activity is presented as the concentration [μM] of flavonoids halving the nitric oxide (NO) production (IC_{50}) in lipopolysaccharide-stimulated macrophages (RAW 264.7).

**Compound**	**NO Production**	**t-test**	
	IC50 [μM]	p	$p \leq 0.05$
Taxifolin (1)	52.4 ± 1.2	0.5959	
6-Bromotaxifolin (**7**)	78.4 ± 7.8	0.0569	
6,8-Dibromotaxifolin (**8**)	62.2 ± 4.2	0.1496	
Quercetin (2)	14.6 ± 2.1	0.0014	*
8-Bromoquercetin (**9**)	>100		
6,8-Dibromoquercetin (**10**)	40.6 ± 2.6	0.0786	
Silybin A (3a)	53.9 ± 4.9	0.6446	
6-Bromosilybin A (**11**)	40.0 ± 3.4	0.0122	*
8-Bromosilybin A (**19**)	36.4 ± 1.7	0.0162	*
6,8-Dibromosilybin A (**12**)	73.9 ± 5.8	0.1930	
6,8,21-Tribromosilybin A (**21**)	70.4 ± 1.7	0.0023	*
Silybin B (3b)	55.6 ± 5.5	0.3993	
6-Bromosilybin B (**13**)	37.0 ± 2.4	0.0468	*
6-Chlorosilybin B (**28**)	52.4 ± 4.5	0.4386	
8-Bromosilybin B (**20**)	38.0 ± 2.2	0.0071	*
6,8-Dibromosilybin B (**14**)	90.4 ± 4.6	0.0123	*
6,8,21-Tribromosilybin B (**22**)	>100		
2,3-Dehydrosilybin AB (4)	27.9 ± 0.5	0.0000	*
8-Bromo-2,3-dehydrosilybin AB (**16**)	>25		
6,8-Dibromo-2,3-dehydrosilybin AB (**17**)	44.4 ± 2.4	0.1855	
Silychristin A (**5**)	72.5 ± 5.4	0.0567	
6,8-Dibromosilychristin A (**15**)	>100		
2,3-Dehydrosilychristin A (**6**)	42.1 ± 2.7	0.0903	
8-Bromo-2,3-dehydrosilychristin A (**18**)	32.6 ± 1.8	0.0001	*
Indomethacin (PC)	51.3 ± 0.6		

The data are presented as the average of four repetitions with the standard error. T-test was used for the comparison of each halogenated derivative with the positive control (* $p \leq 0.05$). The highest tested concentration was 100 μM with except for 8-bromodehydrosilybin AB (**16**), where a concentration higher than 25 μM decreased the cell viability. PC – positive control.

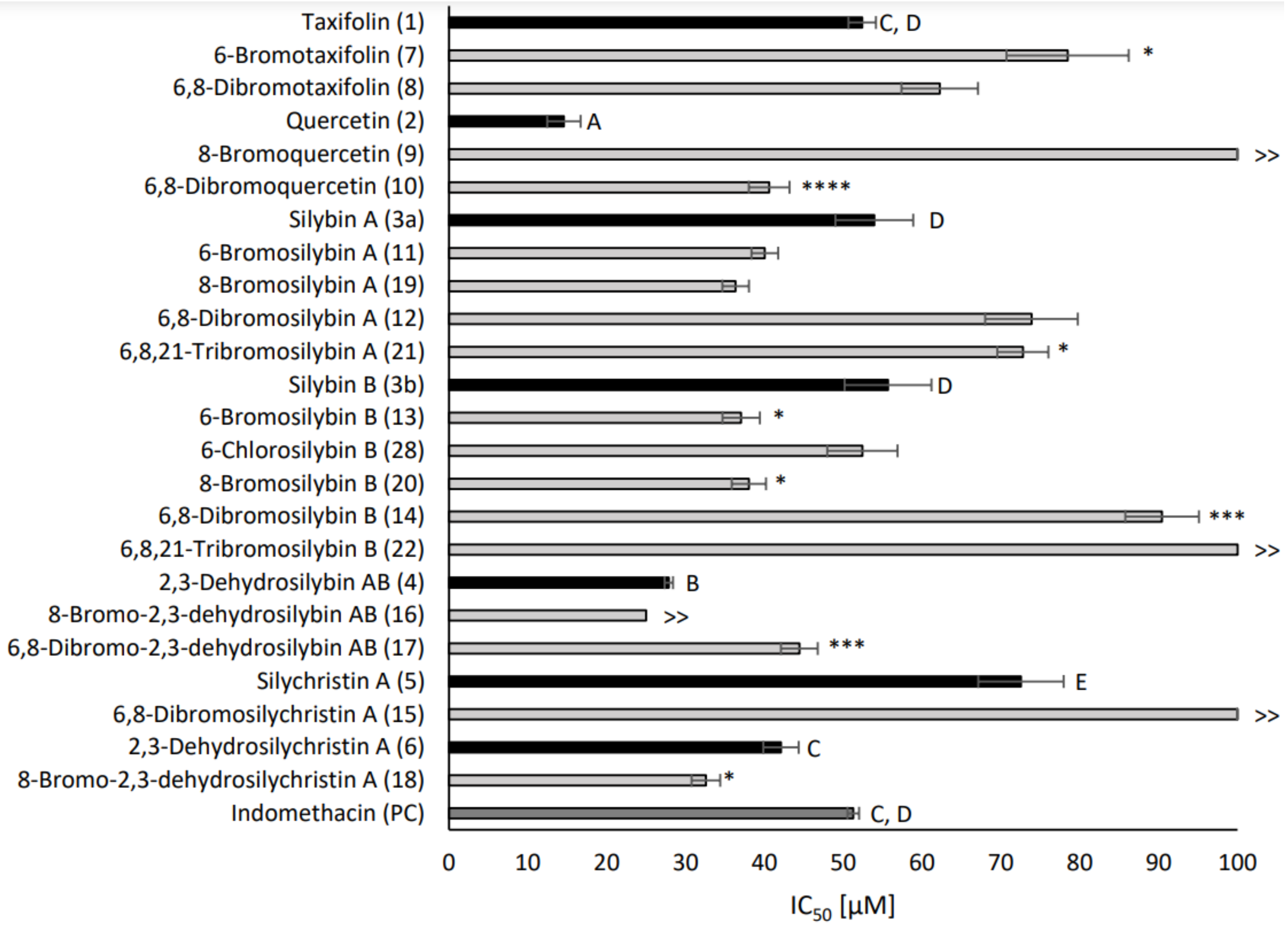

Figure S1. Anti-inflammatory activity of flavonoids and their halogenated derivatives. The anti-inflammatory activity is presented as the concentration [μM] of flavonoids halving the nitric oxide production (IC50) in lipopolysaccharide-stimulated macrophages (RAW 264.7). The data are presented as the average of four repetitions with the standard error. T-test was used for the comparison of each halogenated derivative and its parent compound (* $p \leq 0.05$, ** $p \leq 0.01$, *** $p \leq 0.005$, **** $p \leq 0.001$). ANOVA with Duncan´s post hoc test was used for the comparison of parent compounds and positive control (PC, different capital letters indicate significant differences). The highest tested concentration was 100 μm except for 8-bromo-2,3-dehydrosilybin AB (**16**), where a concentration higher than 25 μm decreased the cell viability. PC—positive control.

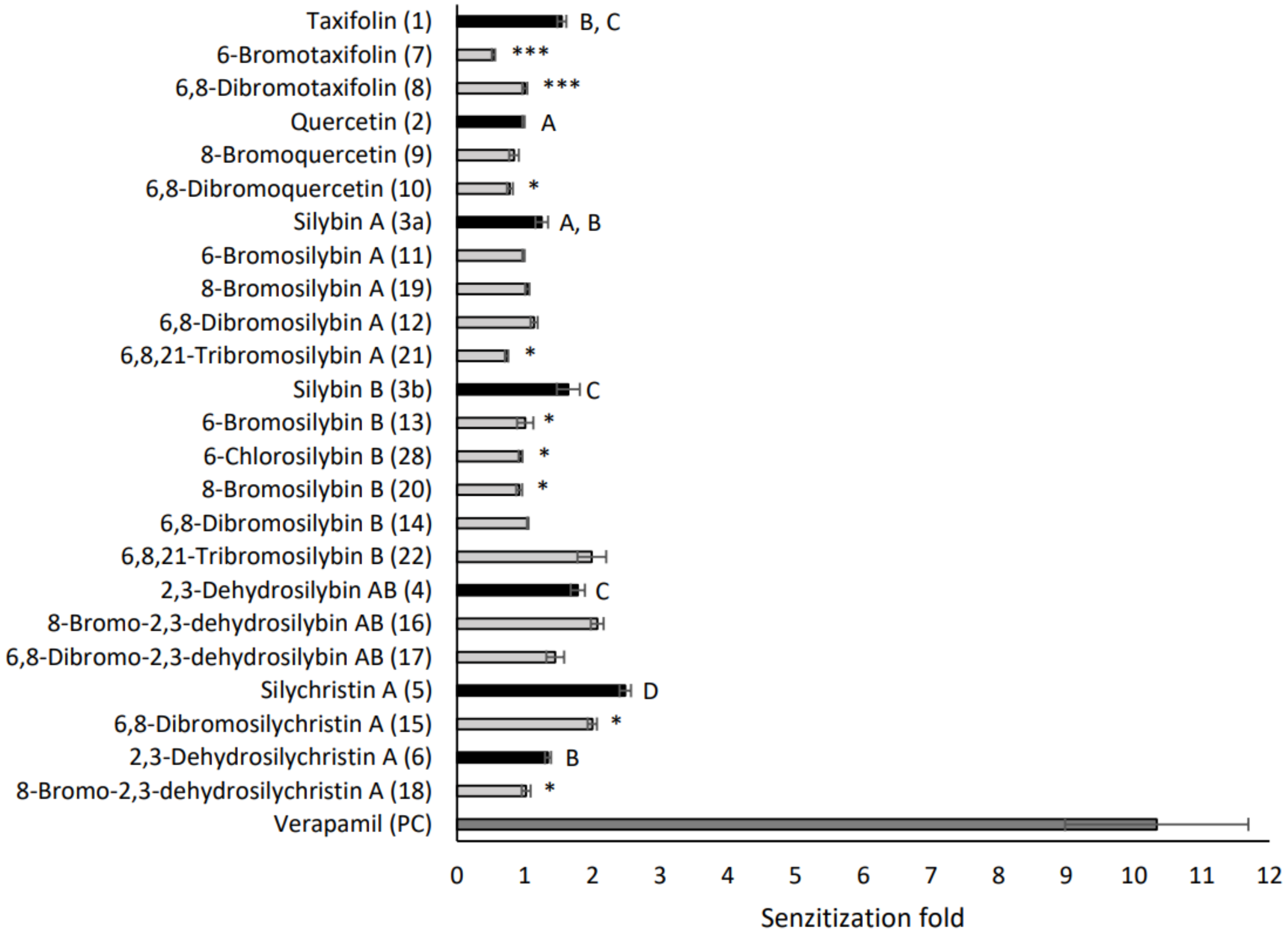

Figure S2. Modulation of doxorubicin-resistant phenotype in human ovarian carcinoma resistant to doxorubicin (hoc/dox) by flavonoids and their halogenated derivatives. Sensitization fold was expressed as the ratio of doxorubicin concentration halving the viability of HOC/DOX and doxorubicin concentration halving the viability of HOC/DOX in the presence of tested compound (10 μM) using verapamil as a positive control (PC). The data are presented as the average of three repetitions with the standard error. T-test was used for the comparison of the halogenated derivative and its parent compound (* $p \leq 0.05$, ** $p \leq 0.01$, *** $p \leq 0.005$, **** $p \leq 0.001$). ANOVA with Duncan´s post hoc test was used for the comparison of parent compounds (different capital letters indicate significant differences). PC—positive control.

NMR Data

Table S5. ^{1}H and ^{13}C NMR data of 8-bromosilybin A (**19**).

Atom #	δC	m.	δH	m.	*J*HH [Hz]
2	82.84	d	5.221	d	11.1
3	71.03	d	4.650	dd	11.1, 6.2
4	197.38	s	-	-	-
4a	100.92	s	-	-	-
5	161.61	s	-	-	-
6	96.17	d	6.144	s	-
7	163.10	s	-	-	-
8	88.10	s	-	-	-
8a	158.41	s	-	-	-
10	78.00	d	4.186	ddd	7.9, 4.5, 2.5
11	75.75	d	4.923	d	7.9
12a	143.17	s	-	-	-
13	116.27	d	7.104	d	2.0
14	129.52	s	-	-	-
15	121.06	d	7.033	dd	8.3, 2.0
16	116.22	d	6.987	d	8.3
16a	143.62	s	-	-	-
17	127.36	s	-	-	-
18	111.70	d	7.019	d	2.0
19	147.52	s	-	-	-
20	146.93	s	-	-	-
21	115.22	d	6.805	d	8.1
22	120.43	d	6.869	dd	8.1, 2.0
23	60.08	t	3.545	ddd	12.2, 4.3, 2.5
			3.352	ddd	12.2, 4.5, 6.1
3-OH	-	-	5.882	d	6.2
5-OH	-	-	11.866	s	-
7-OH	-	-	11.612	br. s	-
19-OMe	55.61	q	3.778	s	-
20-OH	-	-	9.082	s	-
23-OH	-	-	4.900	dd	6.1, 4.3

(DMSO-d6, 30 °C, 600.23 and 150.93 MHz, respectively)

Table S6. ^{1}H and ^{13}C NMR data of 8-bromosilybin B (**20**).

Atom #	δC	m.	δH	m.	*J*HH [Hz]
2	82.89	d	5.226	d	11.1
3	71.11	d	4.656	d	11.1
4	197.55	s	-	-	-
4a	101.03	s	-	-	-
5	161.65	s	-	-	-
6	96.23	d	6.190	s	-
7	163.06	s	-	-	-
8	88.09	s	-	-	-
8a	158.46	s	-	-	-
10	78.05	d	4.201	ddd	7.9, 4.7, 2.5
11	75.85	d	4.913	d	7.9
12a	143.25	s	-	-	-
13	116.41	d	7.100	d	2.0
14	129.57	s	-	-	-
15	121.10	d	7.037	dd	8.3, 2.0
16	116.31	d	6.991	d	8.3
16a	143.69	s	-	-	-
17	127.40	s	-	-	-
18	111.64	d	7.028	d	1.9
19	147.58	s	-	-	-
20	146.96	s	-	-	-
21	115.25	d	6.810	d	8.1
22	120.49	d	6.874	dd	8.1, 1.9
23	60.11	t	3.544	dd	12.3, 2.5
			3.348	dd	12.3, 4.7
3-OH	-	-	n.a.	-	-
5-OH	-	-	11.874 T	br. s	-
7-OH	-	-	11.685 T	br. s	-
19-OMe	55.64	q	3.781	s	-
20-OH	-	-	n.a.	-	-
23-OH	-	-	n.a.	-	-

T—tentative assignment

Table S7. ^{1}H and ^{13}C NMR data of 6,8,21-tribromosilybin A (**21**).

Atom #	δC	m.	δH	m.	*J*HH [Hz]
2	82.94	d	5.287	d	11.4
3	70.90	d	4.746	d	11.4
4	198.01	s	-	-	-
4a	101.32	s	-	-	-
5	158.08	s	-	-	-
6	90.90	s	-	-	-
7	159.19	s	-	-	-
8	89.71	s	-	-	-
8a	157.25	s	-	-	-
10	77.73	d	4.232	ddd	7.9, 4.4, 2.5
11	75.19	d	4.962	d	7.9
12a	143.03	s	-	-	-
13	116.38	d	7.137	d	1.9
14	129.39	s	-	-	-
15	121.32	d	7.048	dd	8.4, 1.9
16	116.35	d	7.005	d	8.4
16a	143.70	s	-	-	-
17	128.40	s	-	-	-
18	110.62	d	7.086	d	1.8
19	148.35	s	-	-	-
20	144.07	s	-	-	-
21	108.95	s	-	-	-
22	123.67	d	7.200	d	1.8
23	60.00	t	3.573	dd	12.4, 2.5
			3.370	dd	12.4, 4.4
3-OH	-	-	6.018^T	br.s	-
5-OH	-	-	12.615	s	-
7-OH	-	-	11.156 T	br.s	-
19-OMe	56.23	q	3.838	s	-
20-OH	-	-	9.610	s	-
23-OH	-	-	4.922^T	br.s	-

(DMSO-d6, 30 °C, 700.13 and 176.05 MHz, respectively); T—tentative assignment; n.a.—.not assigned (due to signal broadening)

Table S8. ^{1}H and ^{13}C NMR data of 6,8,21-tribromosilybin B (**22**).

Atom #	δC	m.	δH	m.	*J*HH [Hz]
2	82.94	d	5.292	d	11.3
3	70.93	d	4.745	d	11.3
4	198.06	s	-	-	-
4a	101.39	s	-	-	-
5	158.09	s	-	-	-
6	90.88	s	-	-	-
7	159.10	s	-	-	-
8	89.68	s	-	-	-
8a	157.25	s	-	-	-
10	77.73	d	4.251	ddd	7.9, 4.5, 2.5
11	75.22	d	4.949	d	7.9
12a	143.06	s	-	-	-
13	116.39	d	7.136	d	2.0
14	129.35	s	-	-	-
15	121.31	d	7.050	dd	8.4, 2.0
16	116.36	d	7.004	d	8.4
16a	143.71	s	-	-	-
17	128.39	s	-	-	-
18	110.58	d	7.094	d	1.8
19	148.37	s	-	-	-
20	144.06	s	-	-	-
21	108.94	s	-	-	-
22	123.67	d	7.200	d	1.8
23	60.00	t	3.573	dd	12.3, 2.5
			3.368	dd	12.3, 4.5
3-OH	-	-	n.a.	-	-
5-OH	-	-	12.618	s	-
7-OH	-	-	n.a.	-	-
19-OMe	56.24	q	3.842	s	-
20-OH	-	-	9.606^{T}	br.s	-
23-OH	-	-	n.a.	-	-

(DMSO-d6, 30 °C,399.87 and 100.55 MHz MHz, respectively); T—tentative assignment; n.a.—not assigned (due signal broadening)

Table S9. ^{1}H and ^{13}C NMR data of 6,8,20-tribromosilychristin A (**23**).

Atom #	δC	m	δH	m	*J*H-H [Hz]
2	83.67	d	5.198	d	11.3
3	71.23	d	4.635	d	11.3
4	197.80	s	-	-	-
4a	101.25	s	-	-	-
5	158.09	s	-	-	-
6	90.87	s	-	-	-
7	159.24	s	-	-	-
8	89.70	s	-	-	-
8a	157.30	s	-	-	-
10	86.13	d	5.510	d	6.8
11	53.39	d	3.489	ddd	7.2, 6.8, 5.6
11a	128.61	s	-	-	-
12	115.13	d	6.893	d	1.7
13	129.53	s	-	-	-
14	115.66	d	6.877	d	1.7
15	140.74	s	-	-	-
15a	146.96	s	-	-	-
16	133.47	s	-	-	-
17	108.92	d	7.022	d	1.9
18	148.39	s	-	-	-
19	143.29	s	-	-	-
20	108.98	s	-	-	-
21	121.48	d	7.119	br.d	1.9
22	62.86	t	3.768	dd	10.8, 5.6
			3.655	dd	10.8, 7.2
3-OH	-	-	n.a.	-	-
5-OH	-	-	12.651	s	-
7-OH	-	-	n.a.	-	-
15-OH	-	-	n.a.	-	-
18-MeO	56.14	q	3.825	s	-
19-OH	-	-	n.a.	-	-
22-OH	-	-	n.a.	-	-

(DMSO-d6, 30 °C, 600.23 and 150.93 MHz, respectively); n.a.—not assigned (due signal broadening)

Table S10. ^{1}H and ^{13}C NMR data of 8-iodosilybin A (**24**).

Atom #	δC	m.	δH	m.	*J*HH [Hz]
2	82.75	d	5.206	d	11.0
3	71.10	d	4.612	br.d	11.0
4	197.32	s	-	-	-
4a	100.79	s	-	-	-
5	162.95	s	-	-	-
6	95.51	d	6.139	s	-
7	165.40	s	-	-	-
8	63.83	s	-	-	-
8a	161.19	s	-	-	-
10	78.00	d	4.214	ddd	8.0, 4.6, 2.4
11	75.83	d	4.907	d	8.0
12a	143.19	s	-	-	-
13	116.28	d	7.105	d	2.0
14	129.62	s	-	-	-
15	120.96	d	7.041	dd	8.4, 2.0
16	116.23	d	6.989	d	8.4
16a	143.58	s	-	-	-
17	127.38	s	-	-	-
18	111.65	d	7.029	d	1.9
19	147.55	s	-	-	-
20	146.93	s	-	-	-
21	115.22	d	6.802	d	8.1
22	120.49	d	6.873	dd	8.1, 1.9
23	60.09	t	3.538	dd	12.2, 2.4
			3.341	dd	12.2, 4.6
3-OH	-	-	5.895	br. s	-
5-OH	-	-	11.931	s	-
7-OH	-	-	11.651	s	-
19-OMe	55.62	q	3.780	s	-
20-OH	-	-	9.105	s	-
23-OH	-	-	4.916	s	-

(DMSO-*d6*, 30 °C, 700.13 and 176.05 MHz, respectively)

Table S11. ^{1}H and ^{13}C NMR data of 6,8-diiodosilybin A (**25**).

Atom #	δC	m.	δH	m.	*J*HH [Hz]
2	82.73	d	5.262	d	11.1
3	70.69	d	4.676	d	11.1
4	197.42	s	-	-	-
4a	101.00	s	-	-	-
5	161.93	s	-	-	-
6	66.71	s	-	-	-
7	163.62	s	-	-	-
8	65.27	s	-	-	-
8a	160.91	s	-	-	-
10	77.99	d	4.218	ddd	8.0, 4.6, 2.5
11	75.80	d	4.913	d	8.0
12a	143.19	s	-	-	-
13	116.23	d	7.112	d	2.0
14	129.36	s	-	-	-
15	120.92	d	7.047	dd	8.4, 2.0
16	116.23	d	6.993	d	8.4
16a	143.62	s	-	-	-
17	127.36	s	-	-	-
18	111.69	d	7.030	d	2.0
19	147.53	s	-	-	-
20	146.93	s	-	-	-
21	115.22	d	6.806	d	8.1
22	120.47	d	6.876	dd	8.1, 2.0
23	60.07	t	3.545	dd	12.3, 2.5
			3.351	dd	12.3, 4.6
3-OH	-	-	n.a.	-	-
5-OH	-	-	12.870	s	-
7-OH	-	-	n.a.	-	-
19-OMe	55.63	q	3.783	s	-
20-OH	-	-	n.a.	-	-
23-OH	-	-	n.a.	-	-

(DMSO-d6, 30 °C, 600.23 and 150.93 MHz, respectively); n.a.—not assigned (due signal broadening)

Table S12. ^{1}H and ^{13}C NMR data of 6,8-diiodosilybin B (**26**).

Atom #	δC	m.	δH	m.	*J*HH [Hz]
2	82.72	d	5.249	d	11.2
3	70.68	d	4.667	d	11.2
4	197.23	s	-	-	-
4a	100.85	s	-	-	-
5	161.97	s	-	-	-
6	66.89	s	-	-	-
7	163.88	s	-	-	-
8	65.49	s	-	-	-
8a	160.91	s	-	-	-
10	77.99	d	4.217	ddd	8.0, 4.6, 2.4
11	75.83	d	4.908	d	8.0
12a	143.21	s	-	-	-
13	116.26	d	7.109	d	2.0
14	129.44	s	-	-	-
15	120.96	d	7.042	dd	8.4, 2.0
16	116.24	d	6.991	d	8.4
16a	143.63	s	-	-	-
17	127.36	s	-	-	-
18	111.65	d	7.028	d	1.9
19	147.54	s	-	-	-
20	146.93	s	-	-	-
21	115.22	d	6.802	d	8.1
22	120.49	d	6.872	dd	8.1, 1.9
23	60.08	t	3.538	dd	12.3, 2.4
			3.341	dd	12.3, 4.6
3-OH	-	-	5.985^{T}	br.s	-
5-OH	-	-	12.893	s	-
7-OH	-	-	10.531 T	br.s	-
19-OMe	55.62	q	3.779	s	-
20-OH	-	-	9.104^{T}	br.s	-
23-OH	-	-	4.885^{T}	br.s	-

(DMSO-d6, 30 °C, 600.23 and 150.93 MHz, respectively; T—tentative assignment; n.a.—not assigned (due signal broadening)

Table S13. ^{1}H and ^{13}C NMR data of 6,8-diiodosilychristin A (**27**).

Atom #	δC	m.	δH	m.	*J*HH [Hz]
2	83.54	d	5.171	d	11.1
3	71.04	d	4.572	d	11.1
4	197.28	s	-	-	-
4a	100.92	s	-	-	-
5	161.95	s	-	-	-
6	66.72	s	-	-	-
7	163.70	s	-	-	-
8	65.29	s	-	-	-
8a	161.01	s	-	-	-
10	86.97	d	5.465	d	7.1
11	53.26	d	3.480	br.ddd	7.1, 6.9, 5.5
11a	128.94	s	-	-	-
12	114.96	d	6.902	dd	1.7, 0.8
13	129.41	s	-	-	-
14	115.37	d	6.863	d	1.7
15	140.66	s	-	-	-
15a	147.03	s	-	-	-
16	132.24	s	-	-	-
17	110.36	d	6.980	d	2.0
18	147.45	s	-	-	-
19	146.27	s	-	-	-
20	115.18	d	6.766	d	8.1
21	118.63	d	6.825	dd	8.1, 2.0
22	62.84	t	3.734	dd	10.8, 5.5
			3.654	dd	10.8, 6.9
3-OH	-	-	n.a.	-	-
5-OH	-	-	12.907	s	-
7-OH	-	-	n.a.	-	-
15-OH	-	-	n.a.	-	-
19-OMe	55.59	q	3.762	s	-
20-OH	-	-	n.a.	-	-
23-OH	-	-	n.a.	-	-

n.a.—not assigned (due to signal broadening)

Table S14. ^{1}H and ^{13}C NMR data **of** 6-chlorosilybin B (**28a**) and 8-chlorosilybin B (**28b**)—(isolated as a mixture containing 6-chloro silybin as a major compound and 8-chloro silybin as a minor compound).

**Atom #**	**δC**	**m.**	**δH**	**m.**	***J*HH [Hz]**
2	82.60	d	5.130	d	11.3
3	71.20	d	4.666	dd	11.3, 6.4
4	198.10	s	-	-	-
4a	100.60	s	-	-	-
5	158.21	s	-	-	-
6	99.63	s	-	-	-
7	161.91	s	-	-	-
8	95.10	d	6.101	s	-
8a	160.09	s	-	-	-
10	78.01	d	4.172	ddd	7.9, 4.7, 2.7
11	75.72	d	4.910	d	7.9
12a	143.16	s	-	-	-
13	116.37	d	7.089	d	2.0
14	129.64	s	-	-	-
15	121.22	d	7.008	dd	8.3, 2.0
16	116.18	d	6.972	d	8.3
16a	143.61	s	-	-	-
17	127.35	s	-	-	-
18	111.68	d	7.011	d	2.0
19	147.52	s	-	-	-
20	146.93	s	-	-	-
21	115.23	d	6.806	d	8.1
22	120.40	d	6.865	dd	8.1, 2.0
23	60.07	t	3.548	m	-
			3.357	m	-
3-OH	-	-	5.876[J]	d	6.4
5-OH	-	-	12.452	s	-
7-OH	-	-	11.593	br. s	-
19-OMe	55.62	q	3.779	s	-
20-OH	-	-	9.083	s	-
23-OH	-	-	4.902	br. m	-

Atom #	δC	m.	δH	m.	*J*HH [Hz]
2	82.91	d	5.224	d	11.1
3	71.03	d	4.673	dd	11.1, 6.3
4	197.56	s	-	-	-
4a	100.85	s	-	-	-
5	160.73	s	-	-	-
6	96.21	d	6.145	s	-
7	162.04	s	-	-	-
8	98.56	s	-	-	-
8a	157.27	s	-	-	-
10	78.01	d	4.184	ddd	7.9, 4.6, 2.5
11	75.75	d	4.923	d	7.9
12a	143.19	s	-	-	-
13	116.37	d	7.107	d	2.0
14	129.46	s	-	-	-
15	121.15	d	7.035	dd	8.3, 2.0
16	116.24	d	6.990	d	8.3
16a	143.67	s	-	-	-
17	127.35	s	-	-	-
18	111.70	d	7.018	d	2.1
19	147.52	s	-	-	-
20	146.93	s	-	-	-
21	115.23	d	6.806	d	8.1
22	120.43	d	6.870	dd	8.1, 2.1
23	60.07	t	3.548	m	-
			3.357	m	-
3-OH	-	-	5.887[J]	d	6.3
5-OH	-	-	11.811	s	-
7-OH	-	-	11.593	br. s	-
19-OMe	55.62	q	3.779	s	-
20-OH	-	-	9.083	s	-
23-OH	-	-	4.902	br. m	-

[J]—J-resolved readout

NMR Spectra

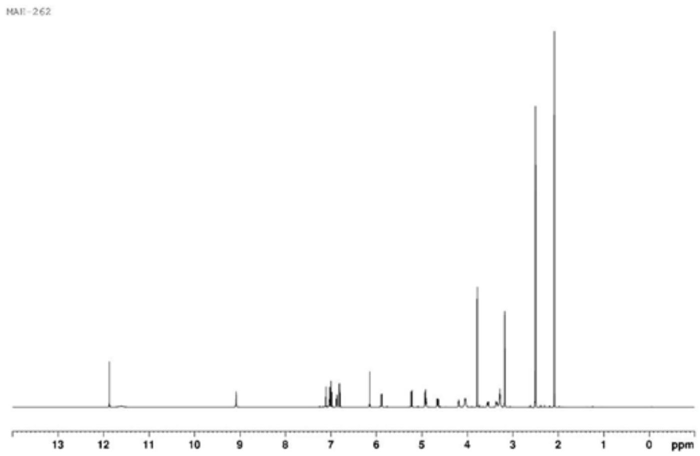

Figure S3. 1H NMR spectrum of 8-bromosilybin A (19). (600.23 MHz, DMSO-d6, 30 °C).

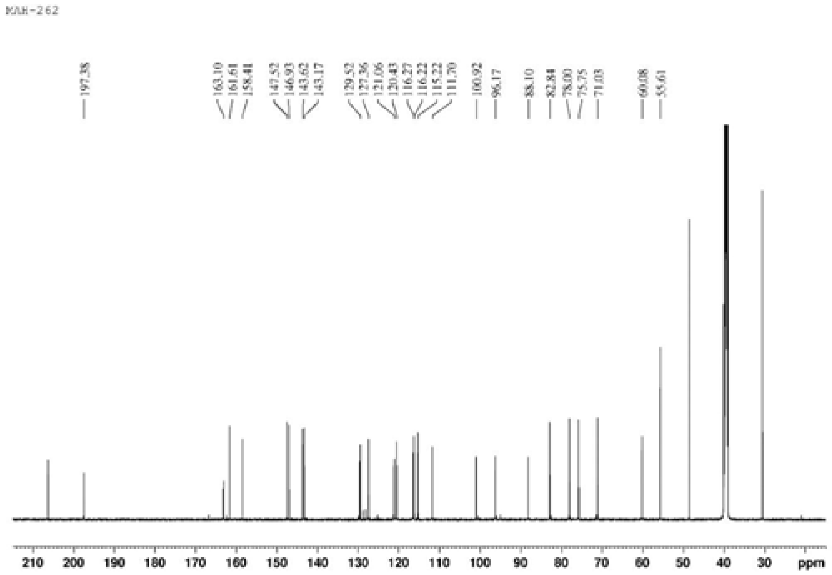

Figure S4. ^{13}C NMR spectrum of 8-bromosilybin A (**19**). (150.93 MHz, DMSO-d_6, 30 °C).

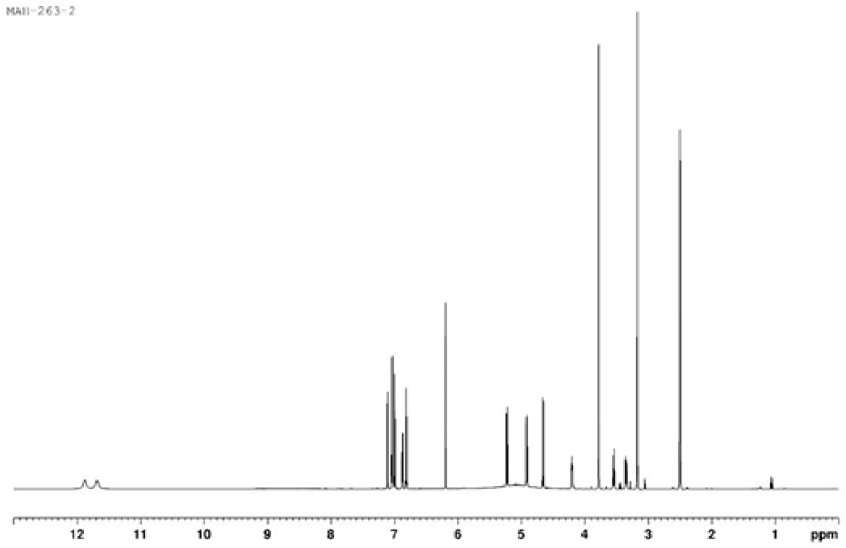

Figure S5. ^{1}H NMR spectrum of 8-bromosilybin B (**20**). (600.23 MHz, DMSO-*d*$_6$, 30 °C).

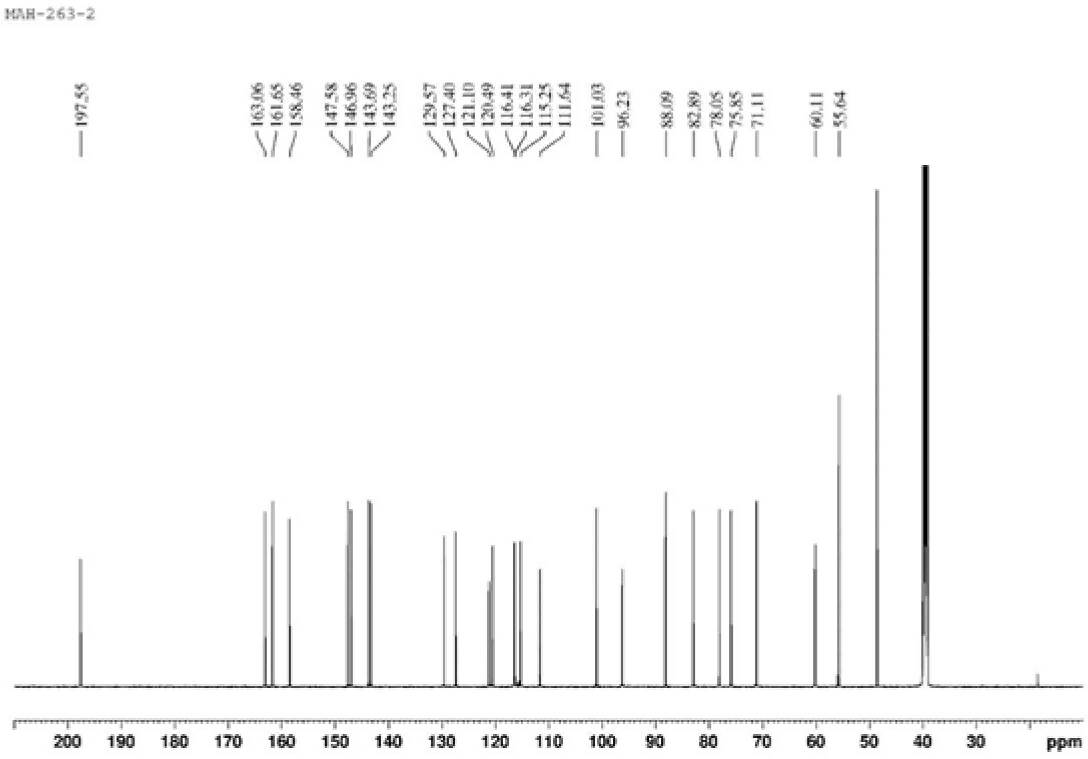

Figure S6. 13C NMR spectrum of 8-bromosilybin B (20). (150.93 MHz, DMSO-d6, 30 °C).

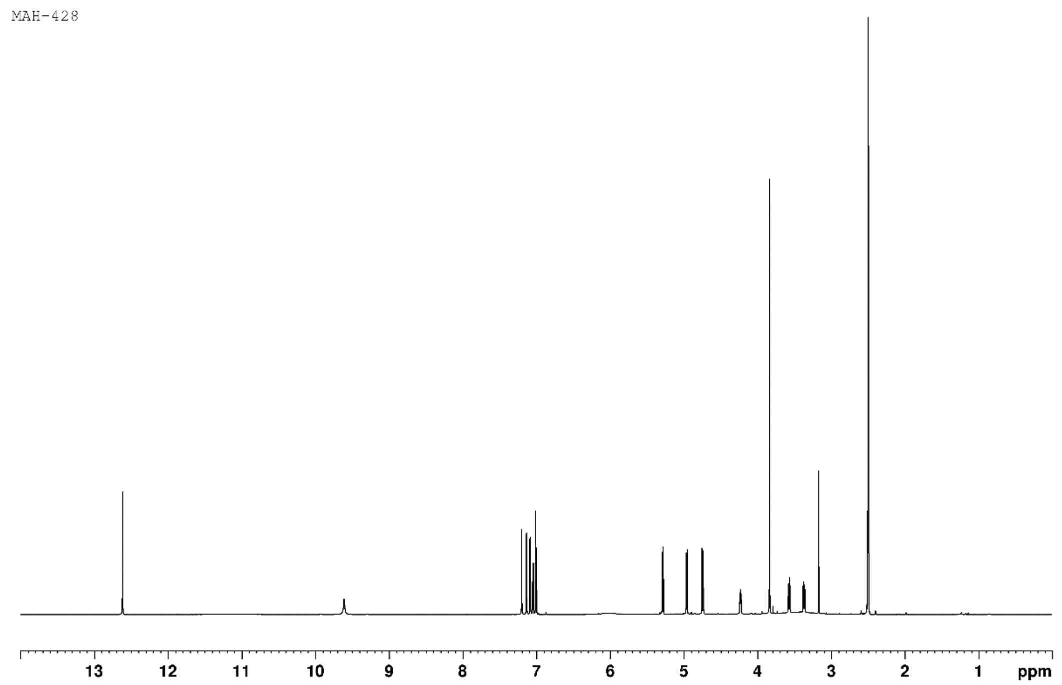

Figure S7. ^{1}H NMR spectrum of 6,8,21-tribromosilybin A (**21**). (700.13 MHz, DMSO-d_6, 30 °C).

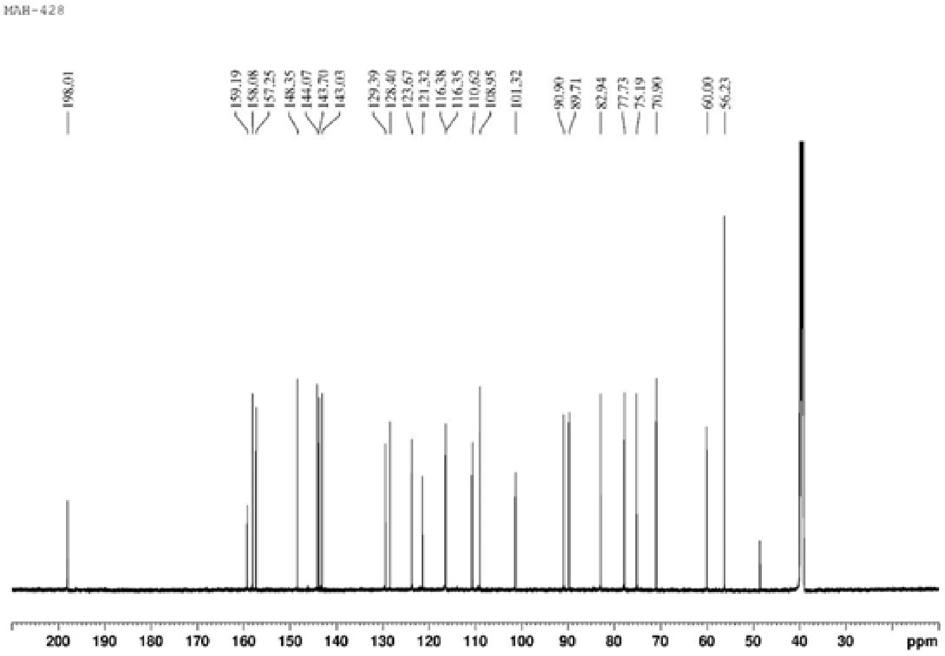

Figure S8. ^{13}C NMR spectrum of 6,8,21-tribromosilybin A (**21**). (176.05 MHz, DMSO-d_6, 30 °C).

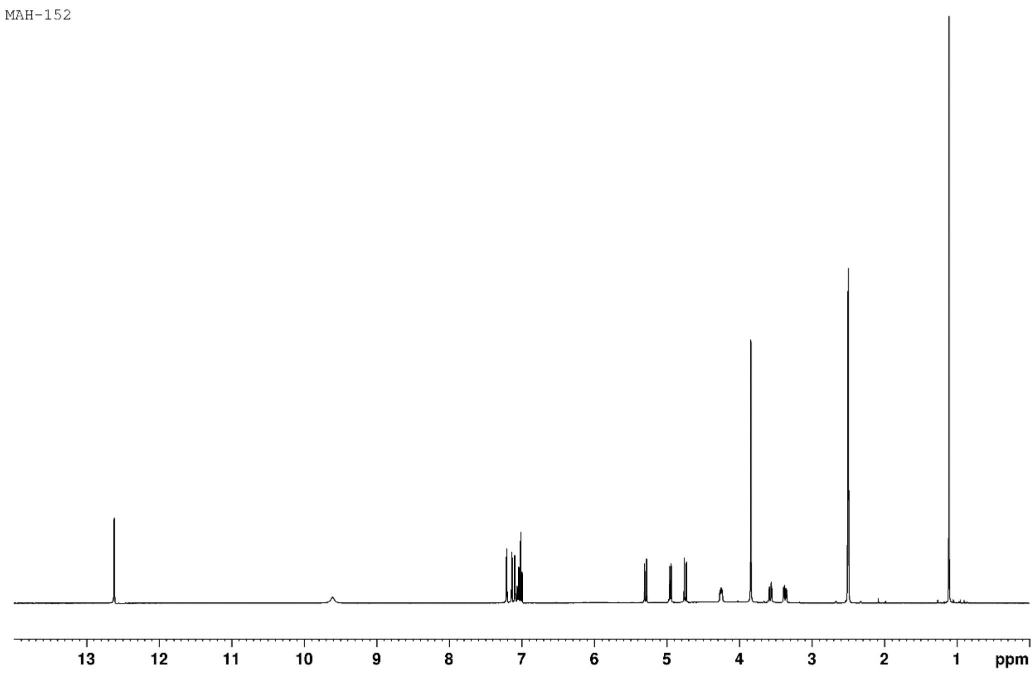

Figure S9. ^{1}H NMR spectrum of 6,8,21-tribromosilybin B (**22**). (399.87 MHz, DMSO-d_6, 30 °C).

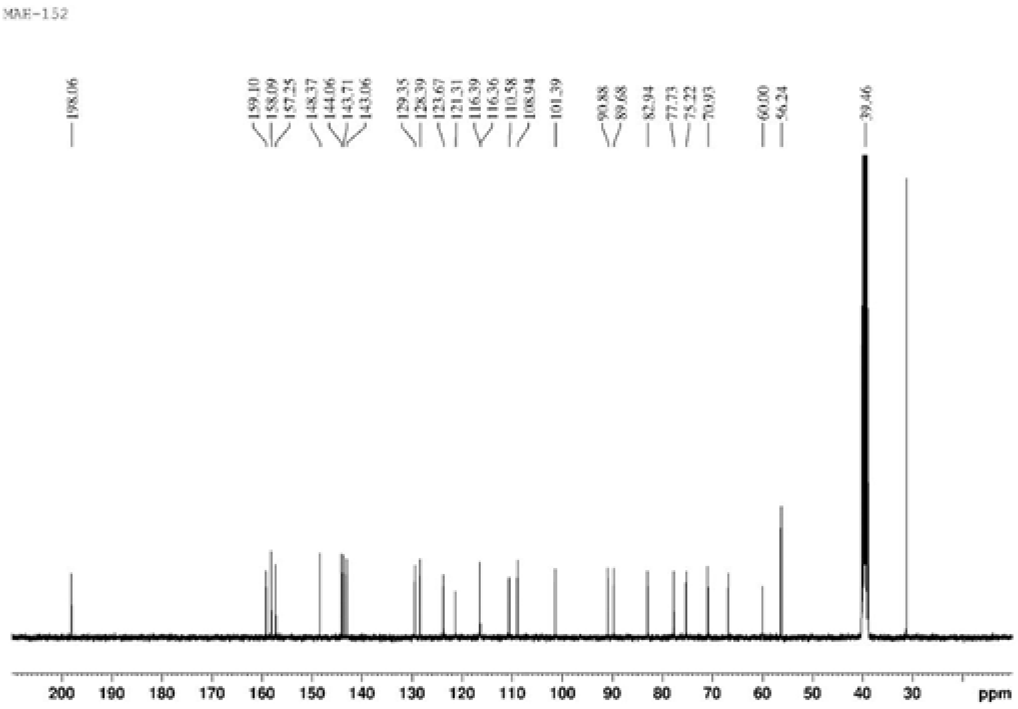

Figure S10. ^{13}C NMR spectrum of 6,8,21-tribromosilybin B (**22**). (100.55 MHz, DMSO-d_6, 30 °C).

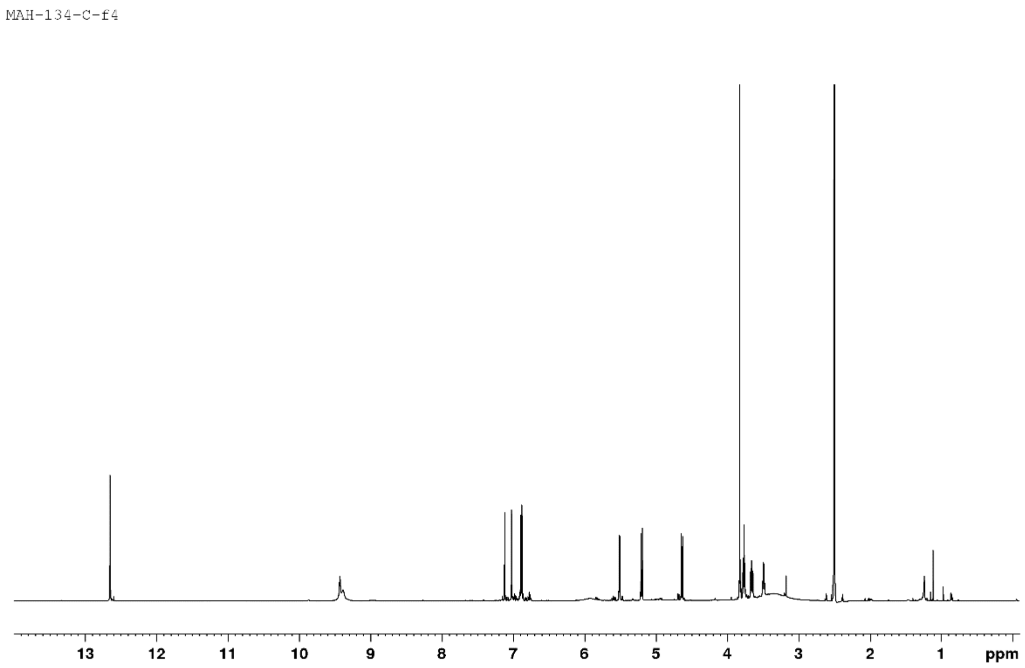

Figure S11. ^{1}H NMR spectrum of 6,8,20-tribromosilychristin A (**23**). (600.23 MHz, DMSO-*d*$_6$, 30 °C).

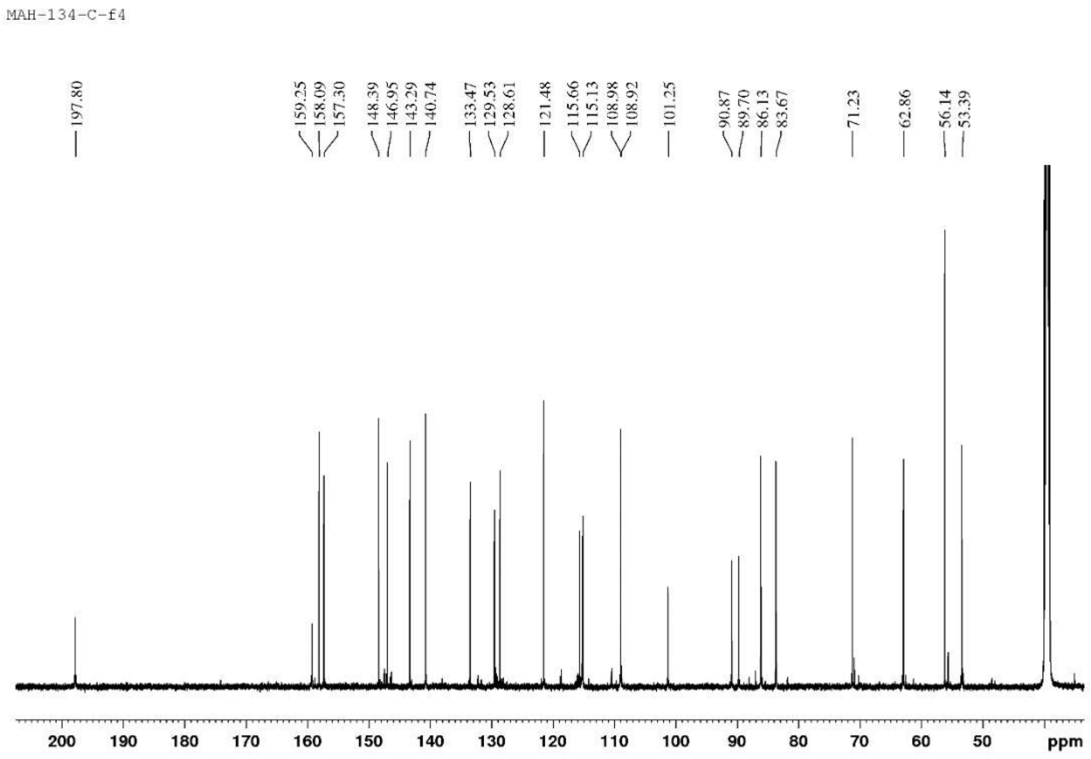

Figure S12. ^{13}C NMR spectrum of 6,8,20-tribromosilychristin A (**23**). (150.93 MHz, DMSO-d_6, 30 °C).

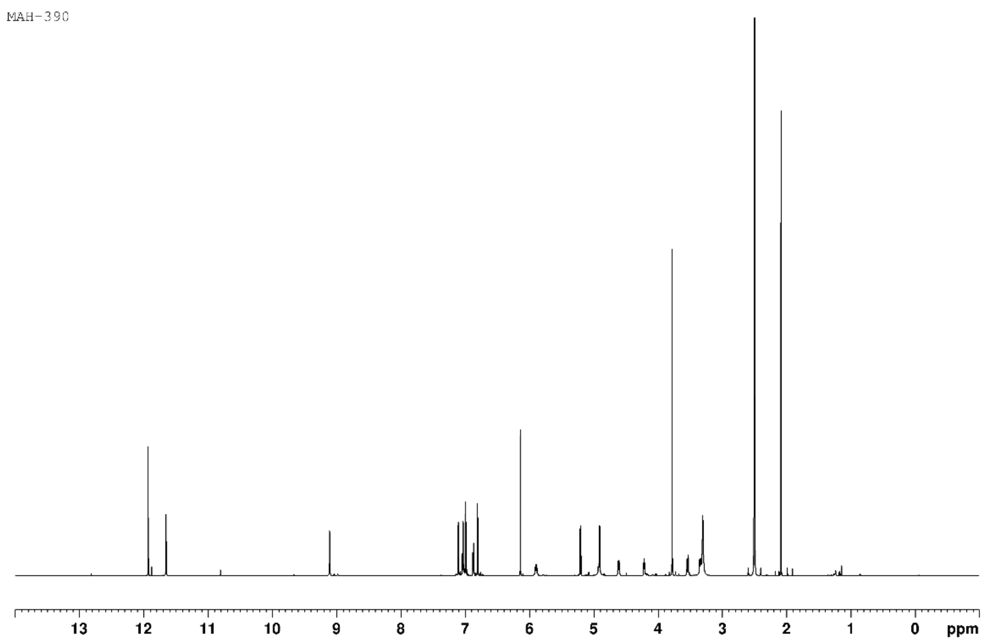

Figure S13. ^{1}H NMR spectrum of 8-iodosilybin A (**24**). (700.13 MHz, DMSO-*d*$_6$, 30 °C).

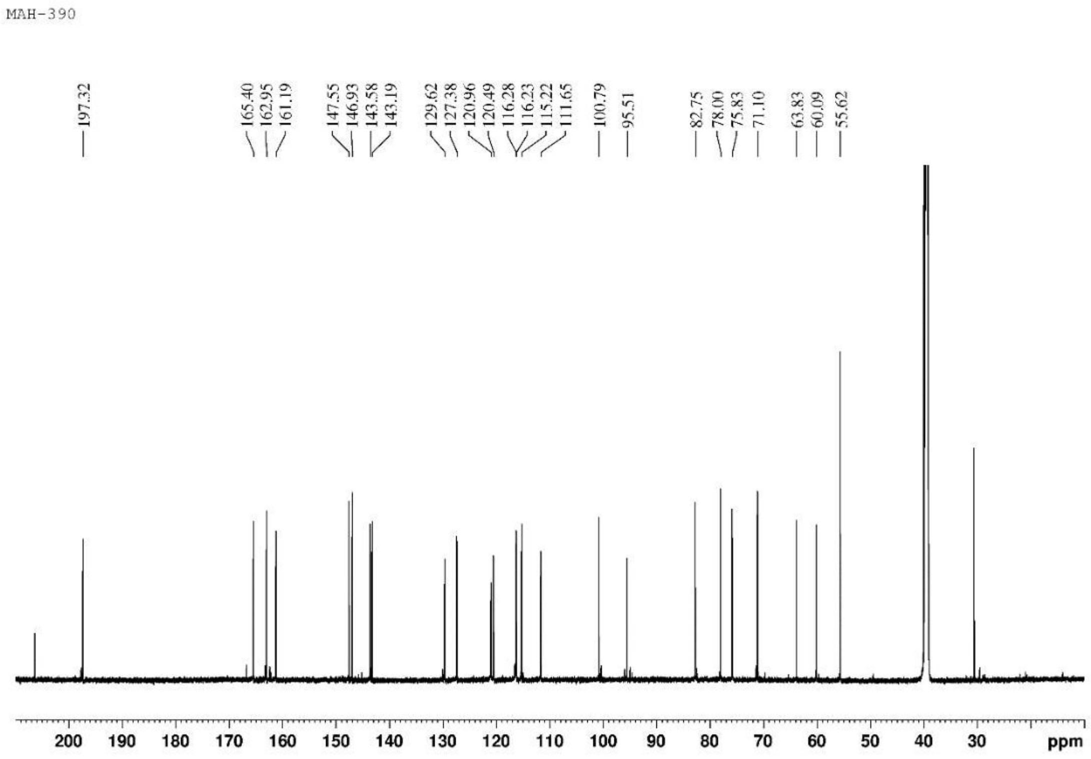

Figure S14. ^{13}C NMR spectrum of 8-iodosilybin A (**24**). (176.05 MHz, DMSO-d_6, 30 °C).

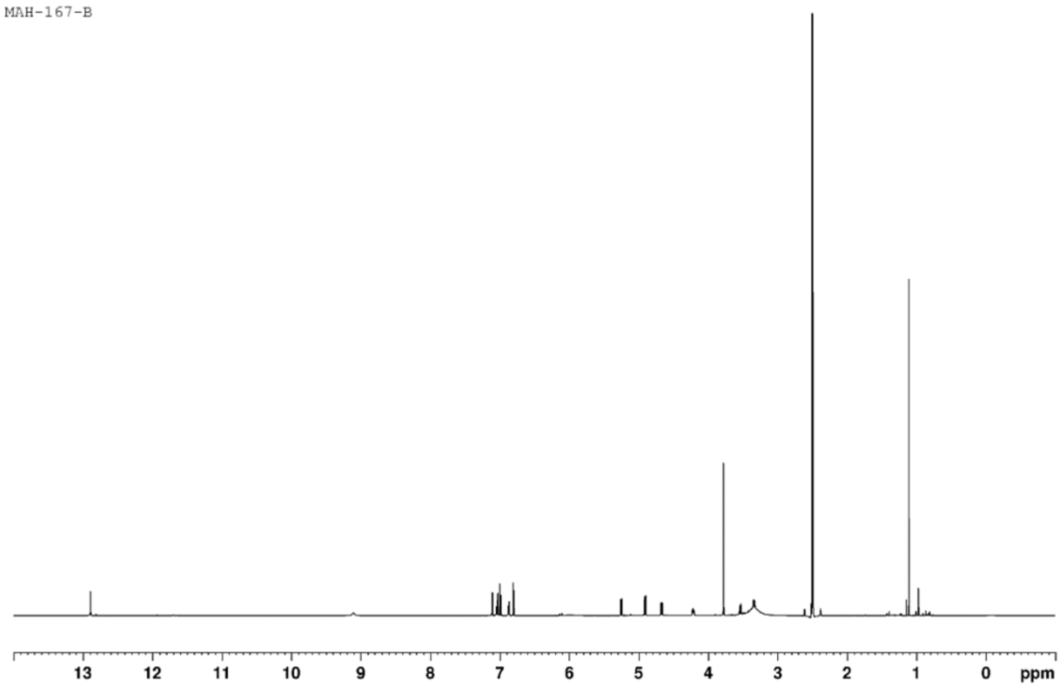

Figure S15. ^{1}H NMR spectrum of 6,8-diiodosilybin A (**25**). (600.23 MHz, DMSO-*d*$_6$, 30 °C).

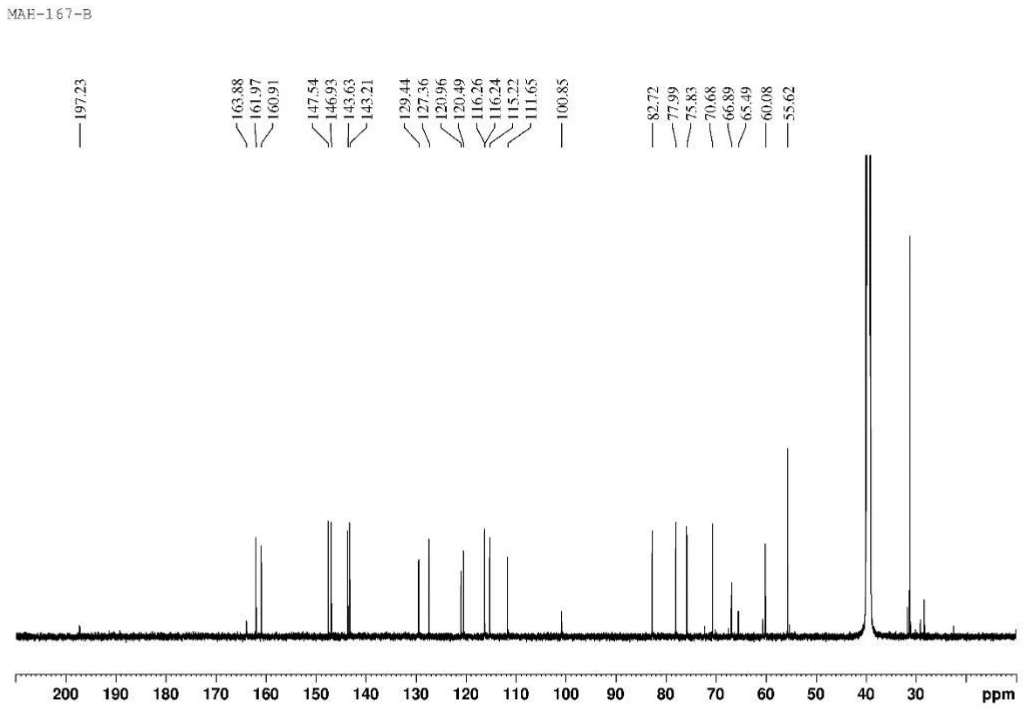

Figure S16. ^{13}C NMR spectrum of 6,8-diiodosilybin A (**25**). (150.93 MHz, DMSO-d_6, 30 °C).

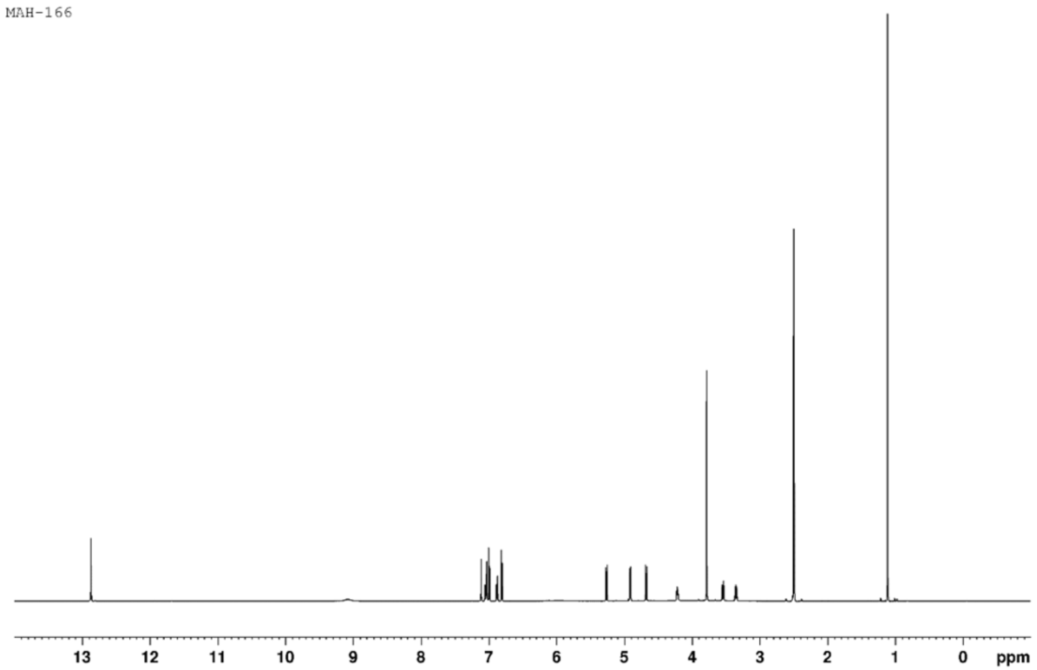

Figure S17. 1H NMR spectrum of 6,8-diiodosilybin B (**26**). (600.23 MHz, DMSO-d6, 30 °C).

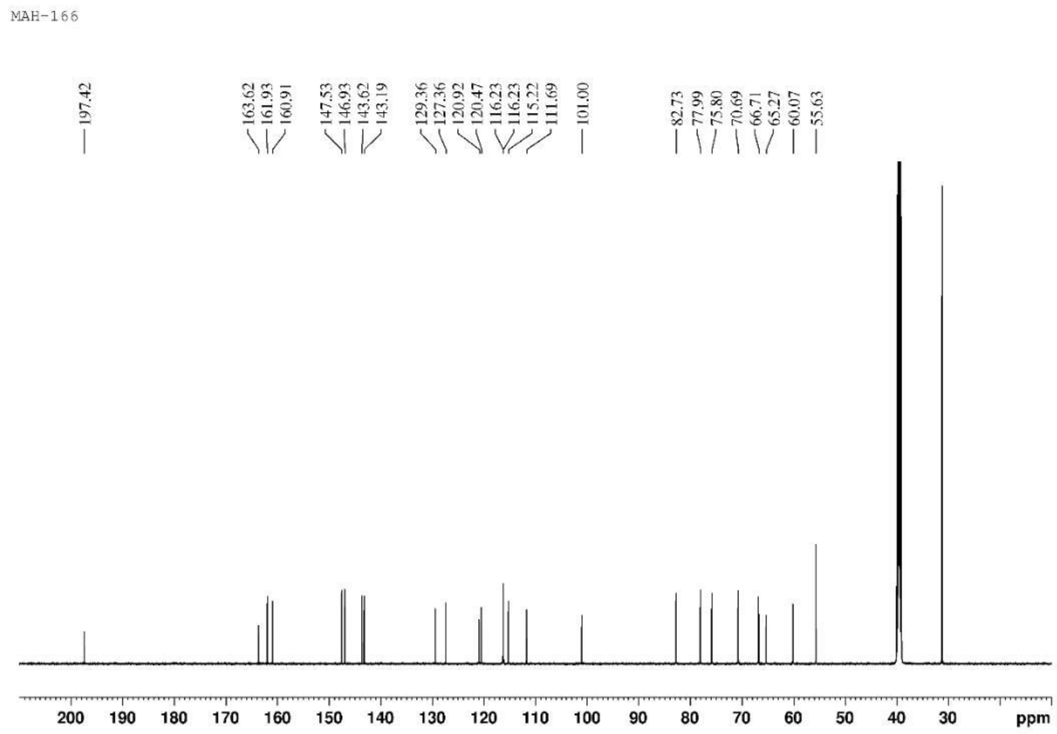

Figure S18. ^{13}C NMR spectrum of 6,8-diiodosilybin B (**26**). (150.93 MHz, DMSO-d_6, 30 °C).

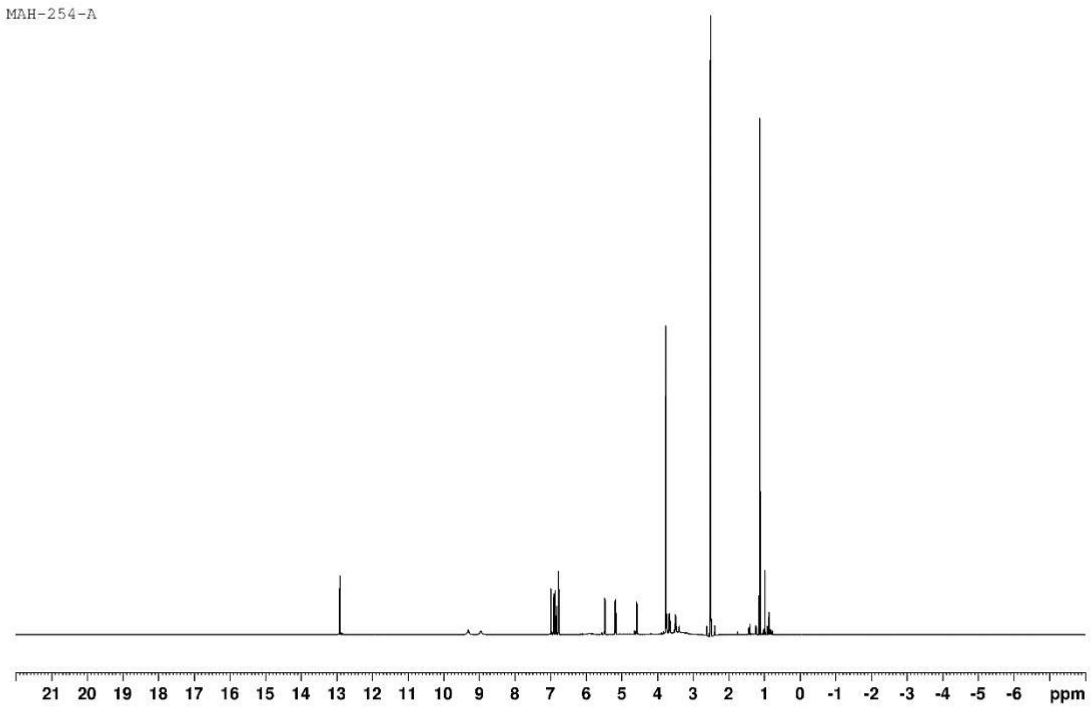

Figure S19. ^{1}H NMR spectrum of 6,8-diiodosilychristin A (**27**). (600.23 MHz, DMSO-d_6, 30 °C).

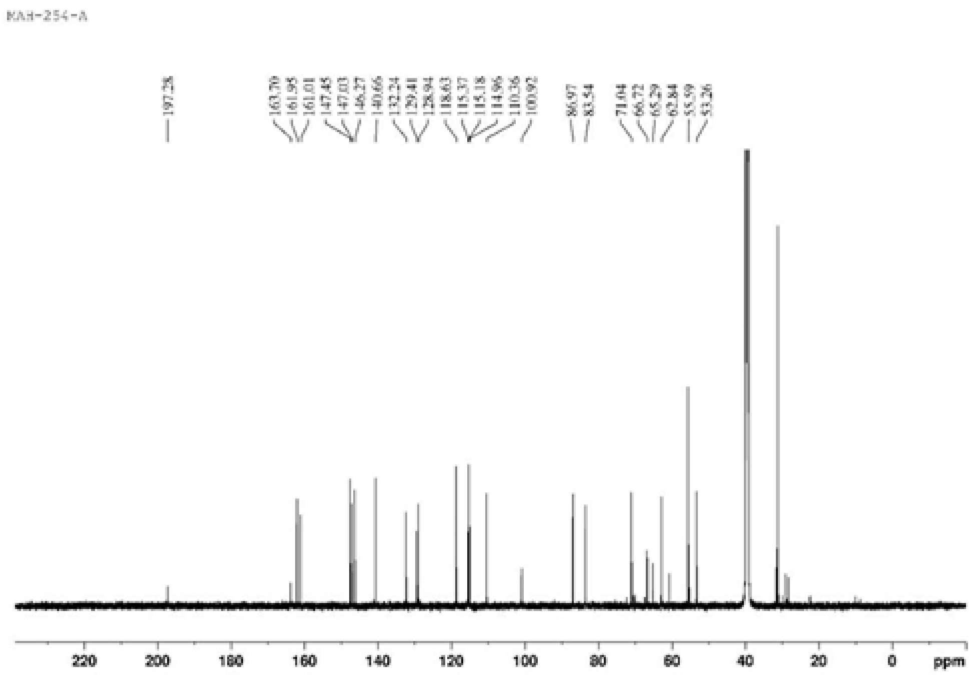

Figure S20. ^{13}C NMR spectrum of 6,8-diiodosilychristin A (**27**). (150.93 MHz, DMSO-d_6, 30 °C).

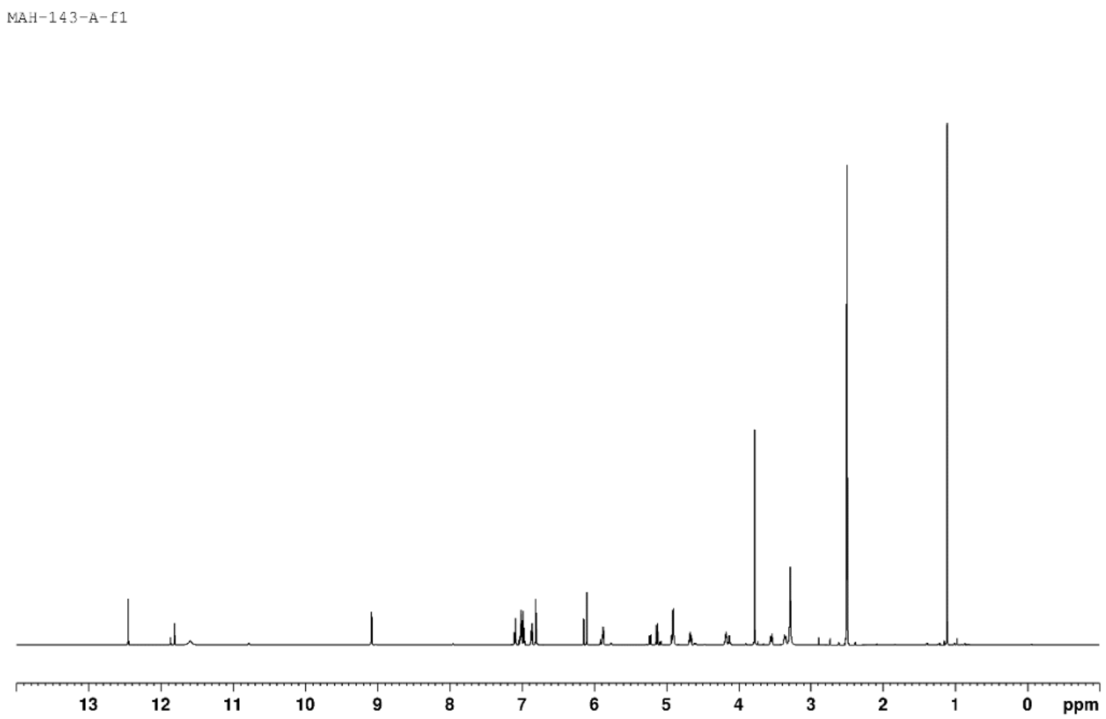

Figure S21. ^{1}H NMR spectrum of 6-chlorosilybin B (**28a**) and 8-chlorosilybin B (**28b**). (600.23 MHz, DMSO-d_6, 30 °C).

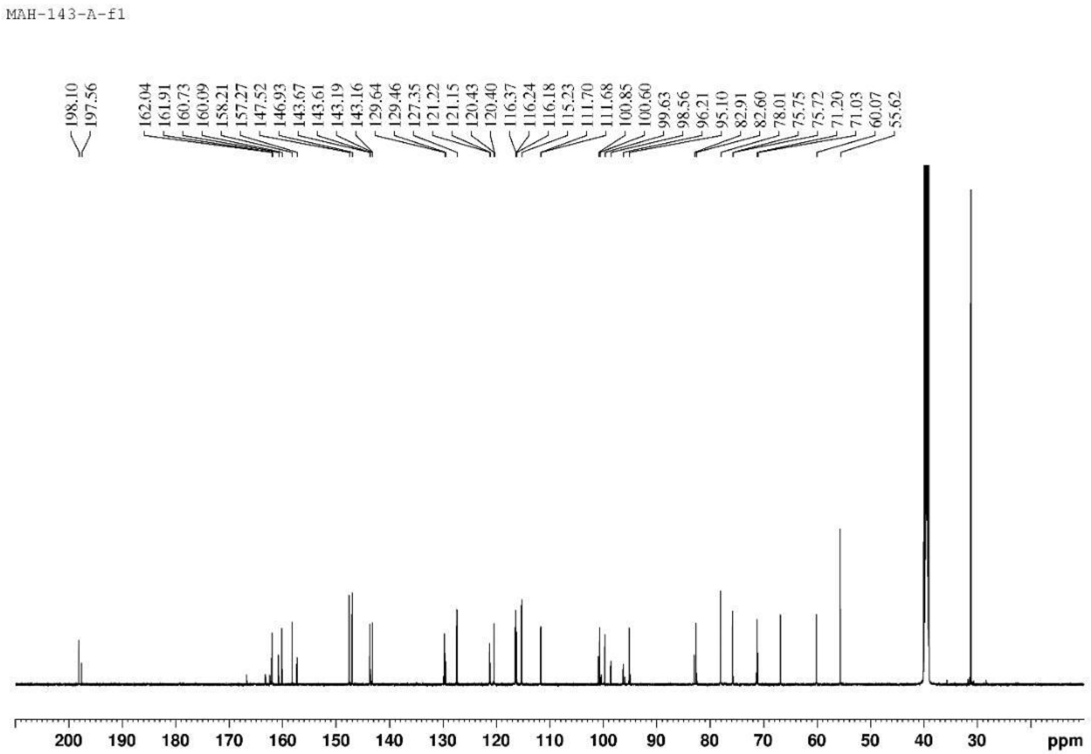

Figure S22. ^{13}C NMR spectrum of 6-chlorosilybin B (**28a**) and 8-chlorosilybin B (**28b**). (150.93 MHz, DMSO-d_6, 30 °C).

HPLC/MS Analysis

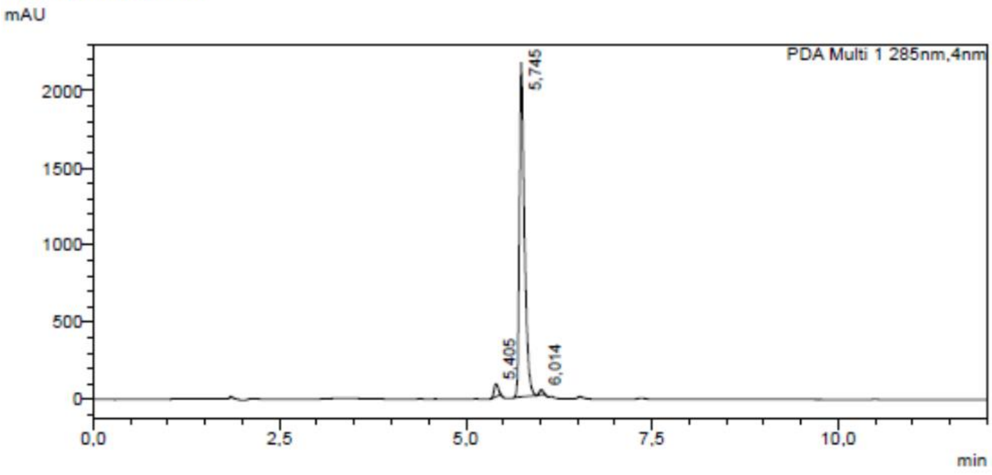

Peak Table

PDA Ch1 285nm

Peak#	Ret. Time	Area	Height	Area%
1	5,405	325220	83641	3,063
2	5,745	10192191	2147474	96,000
3	6,014	99439	30477	0,937
Total		10616851	2261592	100,000

MS Spectrum

Line#:1 R.Time:5,767(Scan#:347)
MassPeaks:613
Spectrum Mode:Single 5,767(347) Base Peak:563(144048)
BG Mode:None Segment 1 - Event 1

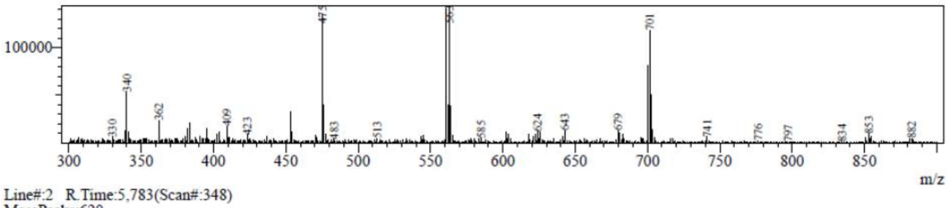

Line#:2 R.Time:5,783(Scan#:348)
MassPeaks:620
Spectrum Mode:Single 5,783(348) Base Peak:561(9227645)
BG Mode:None Segment 1 - Event 2

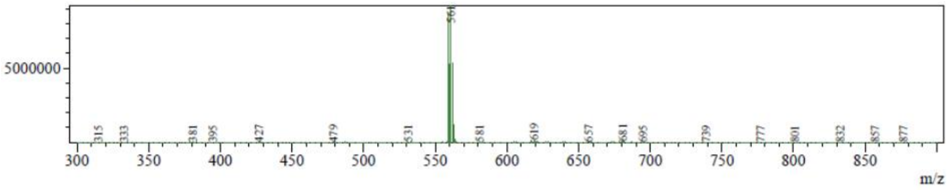

Figure S23. HPLC chromatogram and LC/MS spectra of **8-bromosilybin A (19).** Detected at 285 nm. Chromolith C18, 0.4 mL/min, 25 °C. Product peak 5.745 min, purity 96%.

<Chromatogram>

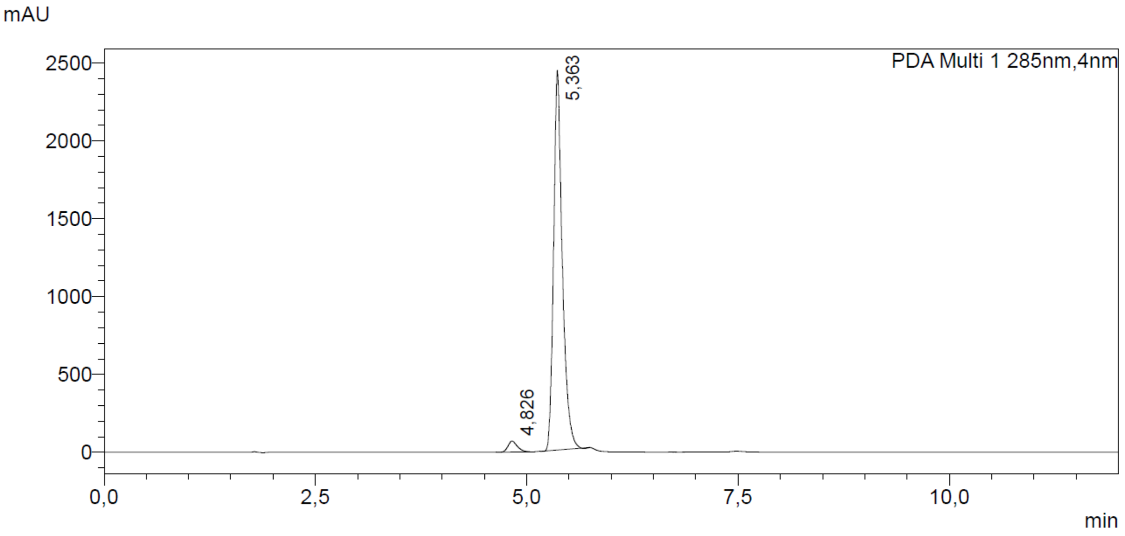

Peak Table

PDA Ch1 285nm

Peak#	Ret. Time	Area	Height	Area%
1	4,826	533260	69923	2,969
2	5,363	17425940	2435586	97,031
Total		17959201	2505509	100,000

MS Spectrum

Line#:1 R.Time:----(Scan#:----)
MassPeaks:224
Spectrum Mode:Averaged 5,167-5,633(311-339) Base Peak:563(125131)
BG Mode:Averaged 1,267-2,233(77-135) Segment 1 - Event 1

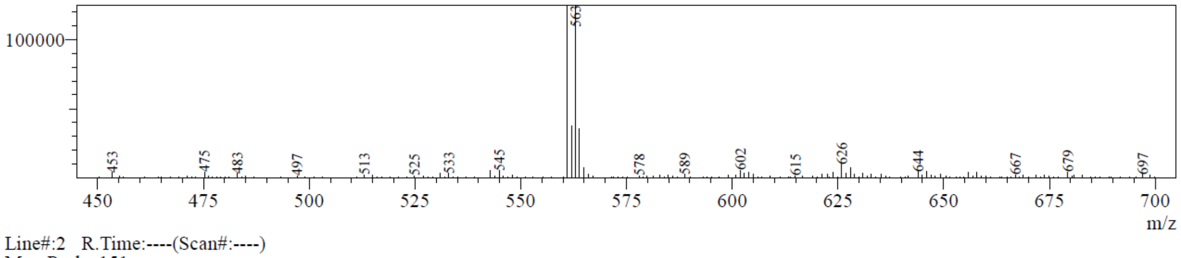

Line#:2 R.Time:----(Scan#:----)
MassPeaks:151
Spectrum Mode:Averaged 5,183-5,650(312-340) Base Peak:561(2301570)
BG Mode:Averaged 1,283-2,250(78-136) Segment 1 - Event 2

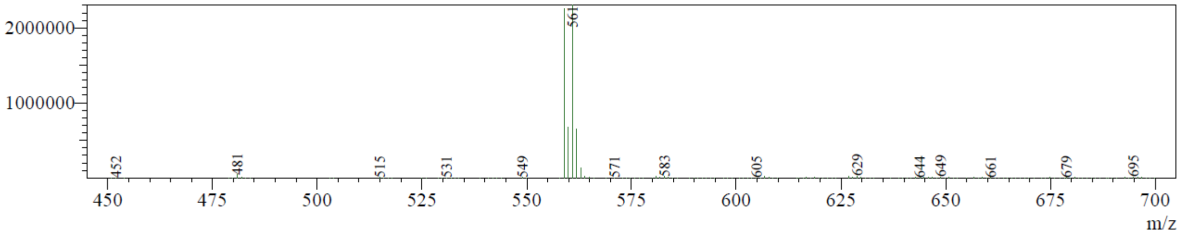

Figure S24. HPLC chromatogram and LC/MS spectra of **8-bromosilybin B (20).** Detected at 285 nm. Chromolith C18, 0.4 mL/min, 25 °C. Product peak 5.363 min, purity 97%.

<Chromatogram>

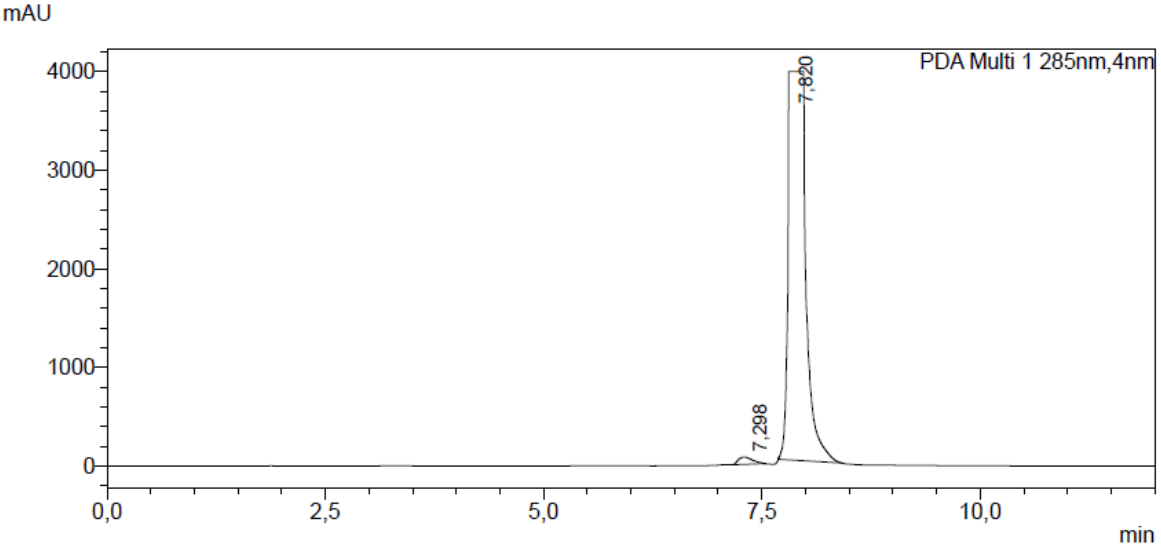

Peak Table

PDA Ch1 285nm

Peak#	Ret. Time	Area	Height	Area%
1	7,298	828623	72019	1,461
2	7,820	55895316	3937295	98,539
Total		56723940	4009314	100,000

MS Spectrum

Line#:1 R.Time:7,950(Scan#:478)
MassPeaks:666
Spectrum Mode:Single 7,950(478) Base Peak:719(6343176)
BG Mode:None Segment 1 - Event 2

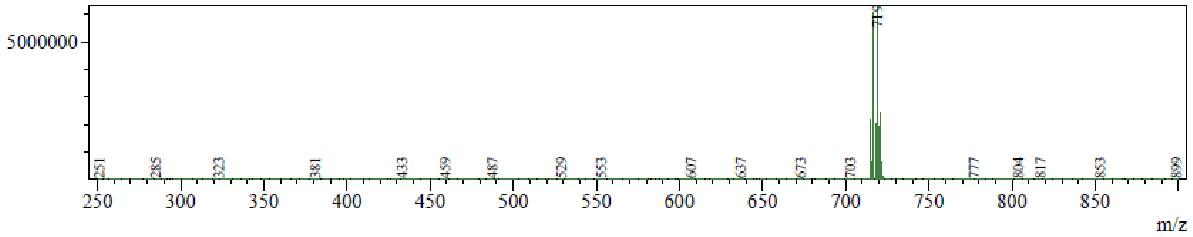

Line#:2 R.Time:7,933(Scan#:477)
MassPeaks:662
Spectrum Mode:Single 7,933(477) Base Peak:475(865515)
BG Mode:None Segment 1 - Event 1

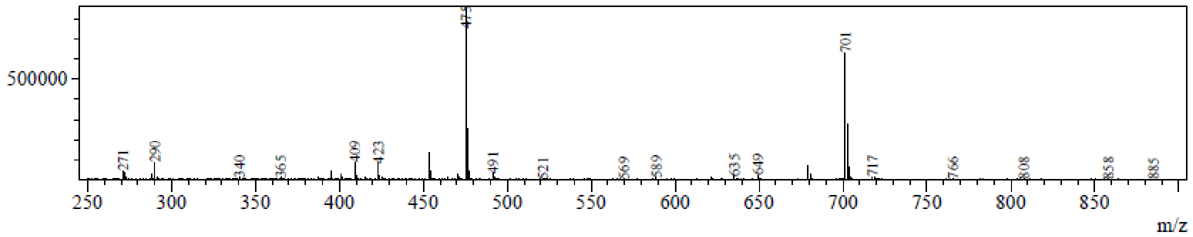

Figure S25. HPLC chromatogram and LC/MS spectra of **6,8,21-tribromosilybin A (21)** Detected at 285 nm. Chromolith C18, 0.4 mL/min, 25 °C. Product peak 7.820 min, purity 96%.

<Chromatogram>

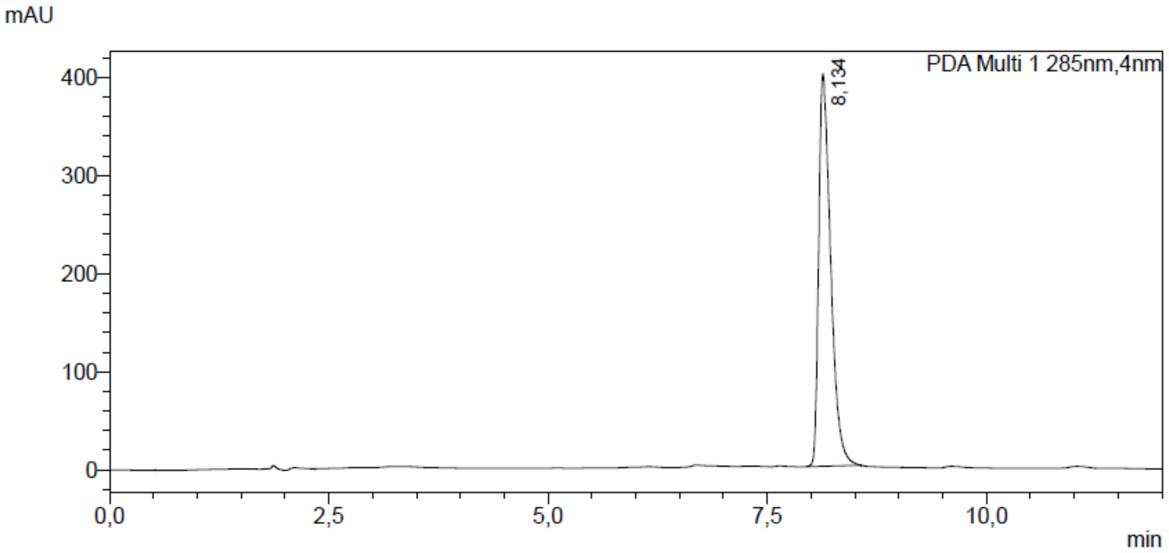

Peak Table

PDA Ch1 285nm

Peak#	Ret. Time	Area	Height	Area%
1	8,134	3800191	399225	100,000
Total		3800191	399225	100,000

MS Spectrum

Line#:1 R.Time:8,150(Scan#:490)
MassPeaks:655
Spectrum Mode:Single 8,150(490) Base Peak:719(1447291)
BG Mode:None Segment 1 - Event 2

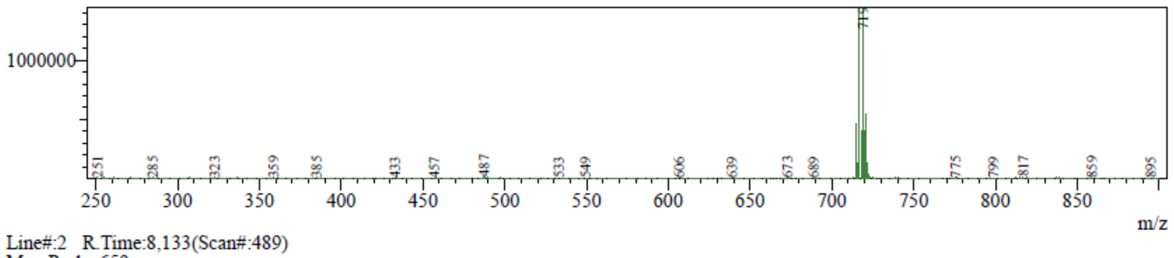

Line#:2 R.Time:8,133(Scan#:489)
MassPeaks:659
Spectrum Mode:Single 8,133(489) Base Peak:475(885898)
BG Mode:None Segment 1 - Event 1

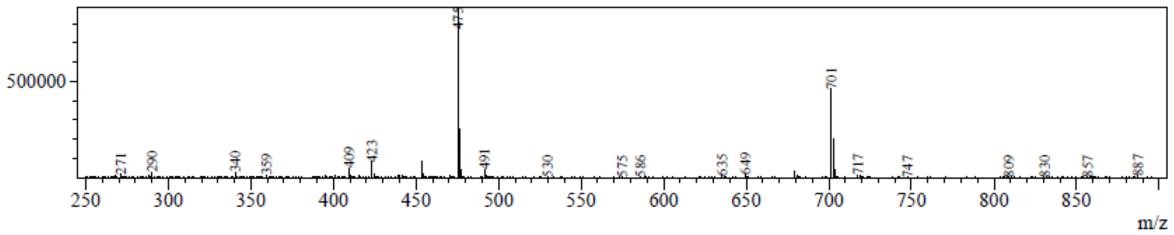

Figure S26. HPLC chromatogram and LC/MS spectra of **6,8,21-tribromosilybin B (22)**

Figure S26. HPLC chromatogram and LC/MS spectra of **6,8,21-tribromosilybin B (22)** Detected at 285 nm. Chromolith C18, 0.4 mL/min, 25 °C. Product peak 8.134 min, purity 100%.

<Chromatogram>

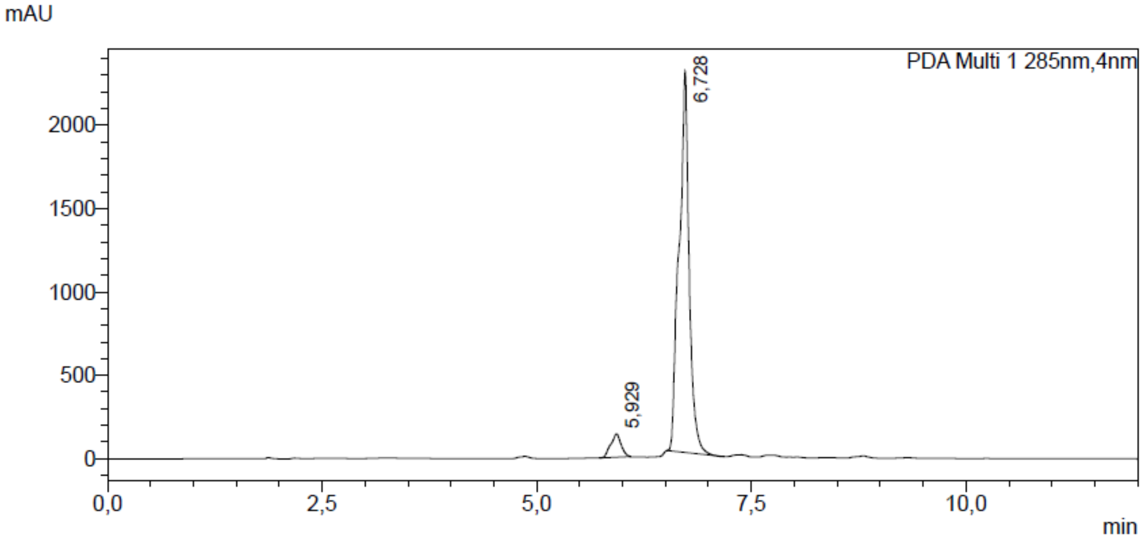

Peak Table

PDA Ch1 285nm

Peak#	Ret. Time	Area	Height	Area%
1	5,929	1165923	139696	5,868
2	6,728	18701588	2275113	94,132
Total		19867511	2414810	100,000

MS Spectrum

Line#:1 R.Time:----(Scan#:----)
MassPeaks:183
Spectrum Mode:Averaged 6,500-7,067(391-425) Base Peak:701(1377205)
BG Mode:Averaged 0,767-2,300(47-139) Segment 1 - Event 1

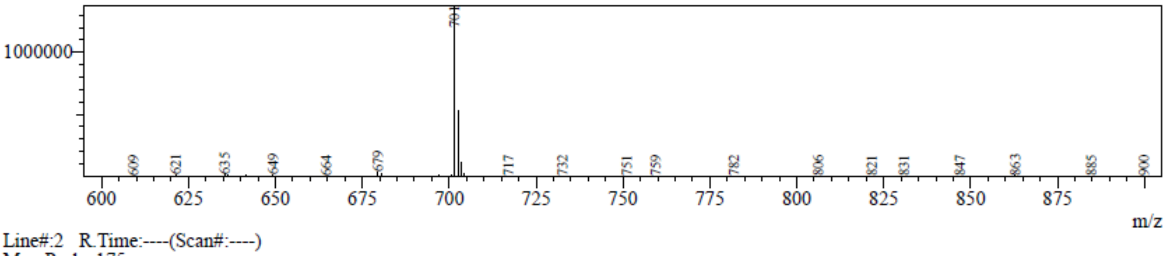

Line#:2 R.Time:----(Scan#:----)
MassPeaks:175
Spectrum Mode:Averaged 6,517-7,083(392-426) Base Peak:719(2986536)
BG Mode:Averaged 0,783-2,317(48-140) Segment 1 - Event 2

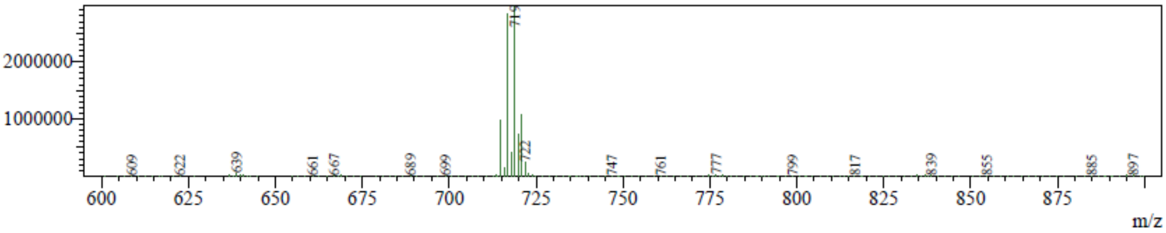

Figure S27. HPLC chromatogram and LC/MS spectra of **6,8,20-tribromosilychristin A (23).** Detected at 285 nm. Chromolith C18, 0.4 mL/min, 25 °C. Product peak 6.728 min, purity 94%. Containing 6% of 6,8-dibromosilychristin A (peak 5.929 min) as an inseparable impurity.

<Chromatogram>

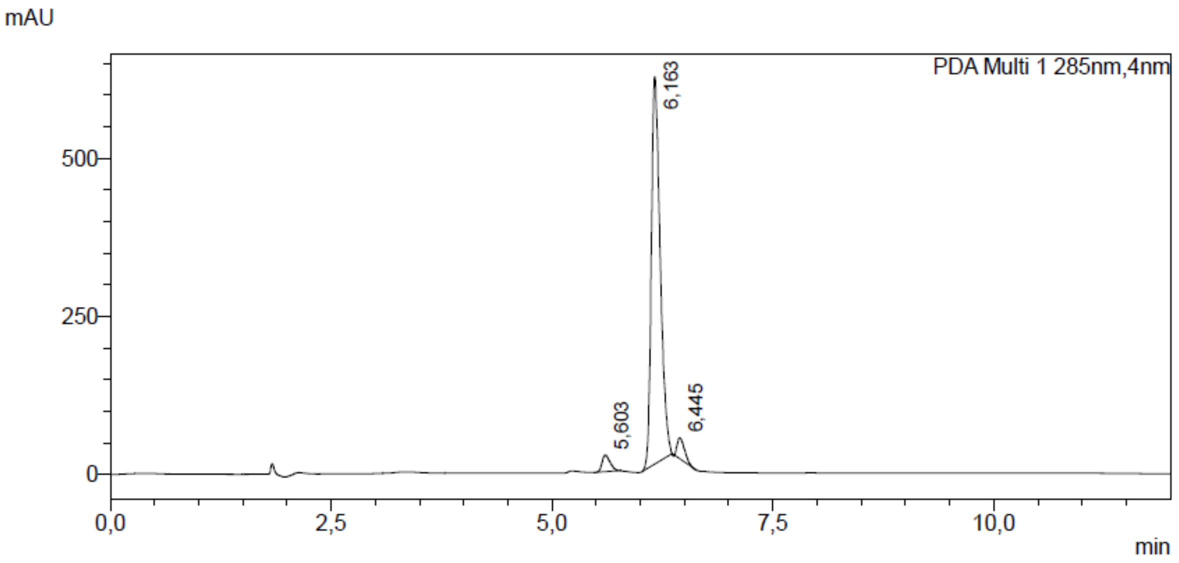

Peak Table

PDA Ch1 285nm

Peak#	Ret. Time	Area	Height	Area%
1	5,603	165799	26206	3,578
2	6,163	4278886	612206	92,333
3	6,445	189513	33027	4,089
Total		4634198	671439	100,000

MS Spectrum

Line#:1 R.Time:----(Scan#:----)
MassPeaks:240
Spectrum Mode:Averaged 6,183-6,250(372-376) Base Peak:607(3397465)
BG Mode:Calc Segment 1 - Event 2

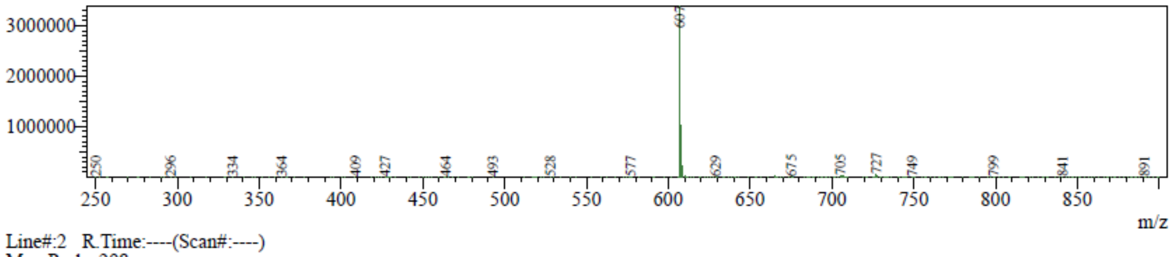

Line#:2 R.Time:----(Scan#:----)
MassPeaks:298
Spectrum Mode:Averaged 6,167-6,233(371-375) Base Peak:701(225540)
BG Mode:Calc Segment 1 - Event 1

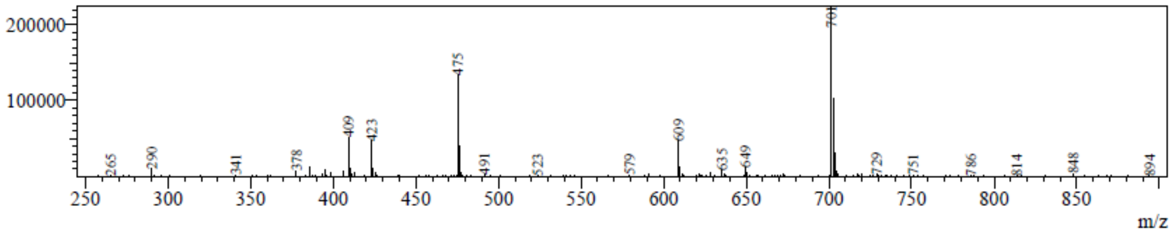

Figure S28. HPLC chromatogram and LC/MS spectra of **8-iodosilybin A (24).** Detected at 285 nm. Chromolith C18, 0.4 mL/min, 25 °C. Product peak 6.163 min, purity 92%. Containing 4% of 6-iodosilychristin A as an inseparable impurity.

<Chromatogram>

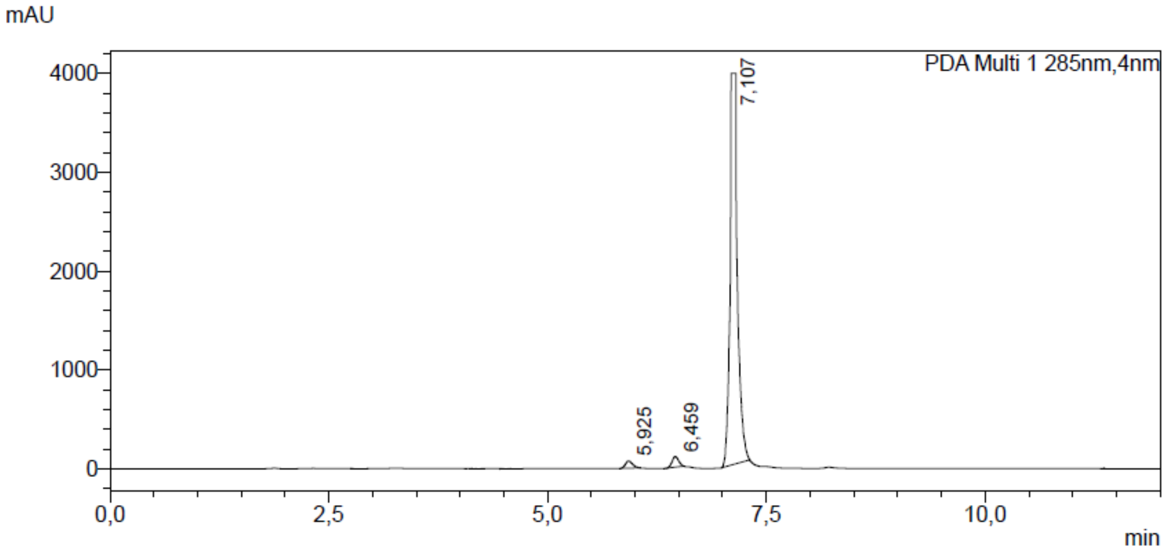

Peak Table

PDA Ch1 285nm

Peak#	Ret. Time	Area	Height	Area%
1	5,925	421525	74103	1,703
2	6,459	565604	108452	2,285
3	7,107	23770285	3961081	96,013
Total		24757413	4143636	100,000

MS Spectrum

Line#:1 R.Time:7,200(Scan#:433)
MassPeaks:402
Spectrum Mode:Single 7,200(433) Base Peak:701(1517537)
BG Mode:None Segment 1 - Event 1

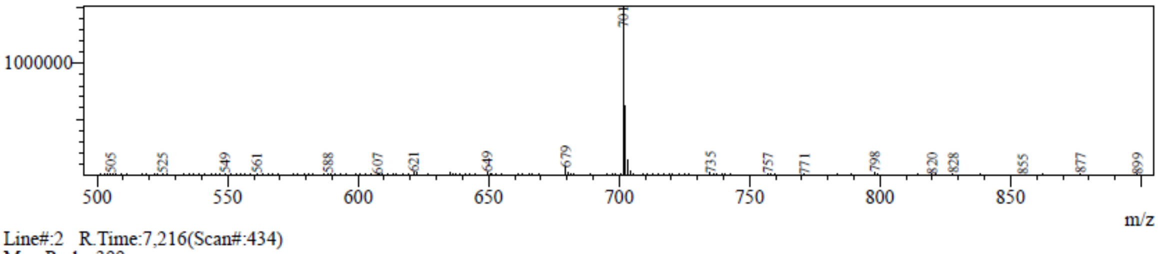

Line#:2 R.Time:7,216(Scan#:434)
MassPeaks:399
Spectrum Mode:Single 7,216(434) Base Peak:733(10014770)
BG Mode:None Segment 1 - Event 2

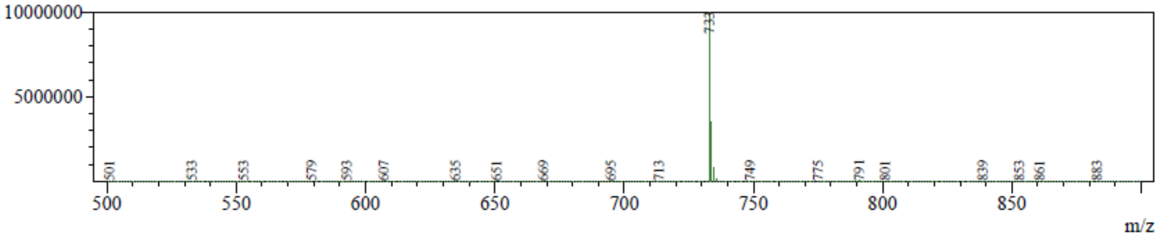

Figure S29. HPLC chromatogram and LC/MS spectra of **6,8-diiodosilybin A (25).** Detected at 285 nm. Chromolith C18, 0.4 mL/min, 25 °C. Product peak 7.107 min, purity 96%.

<Chromatogram>

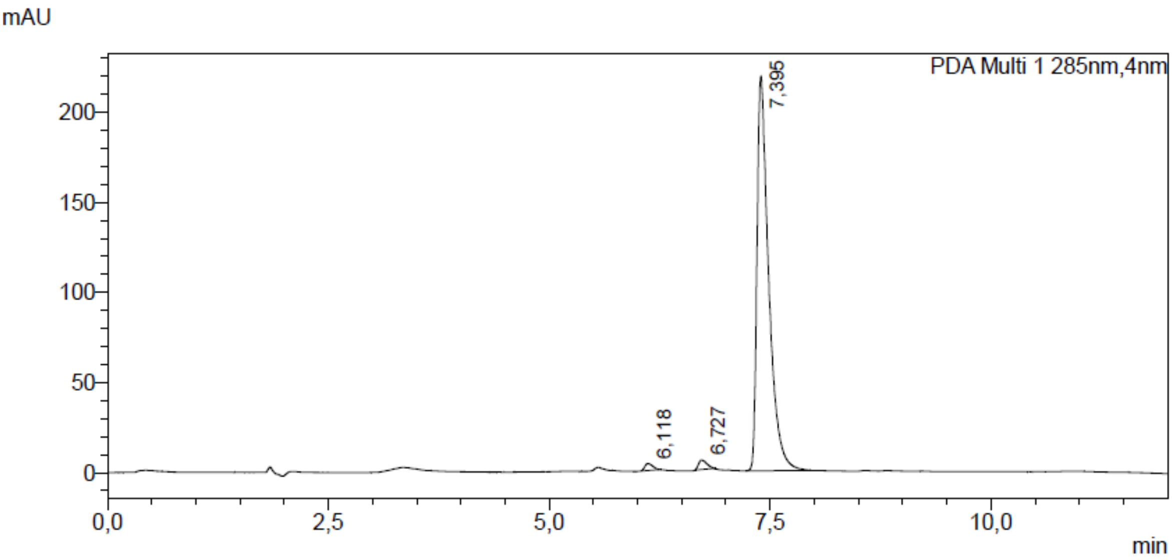

Peak Table

PDA Ch1 285nm

Peak#	Ret. Time	Area	Height	Area%
1	6,118	23736	3817	1,145
2	6,727	34399	5197	1,659
3	7,395	2014874	218746	97,196
Total		2073008	227761	100,000

MS Spectrum

Line#:1 R.Time:7,483(Scan#:450)
MassPeaks:651
Spectrum Mode:Single 7,483(450) Base Peak:733(2807433)
BG Mode:None Segment 1 - Event 2

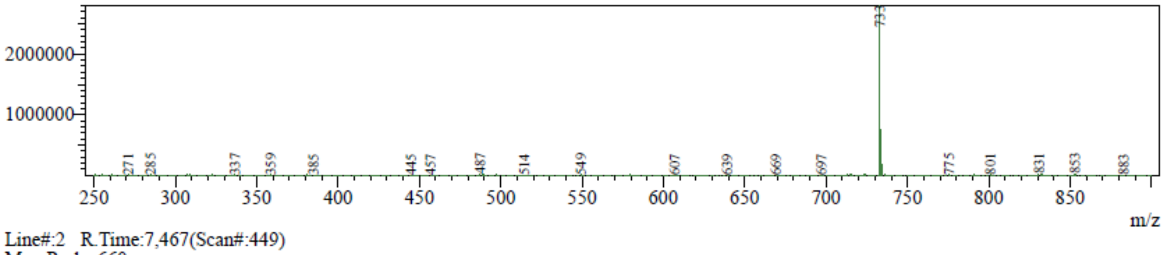

Line#:2 R.Time:7,467(Scan#:449)
MassPeaks:660
Spectrum Mode:Single 7,467(449) Base Peak:475(1076152)
BG Mode:None Segment 1 - Event 1

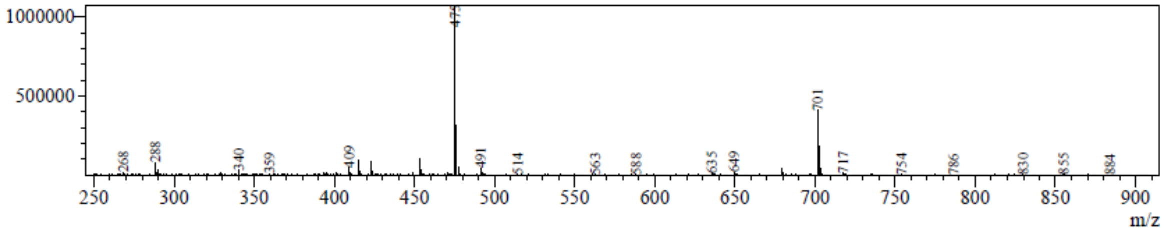

Figure S30. HPLC chromatogram and LC/MS spectra of **6,8-diiodosilybin B (26).** Detected at 285 nm. Chromolith C18, 0.4 mL/min, 25 °C. Product peak 7.395 min, purity 97%.

<Chromatogram>

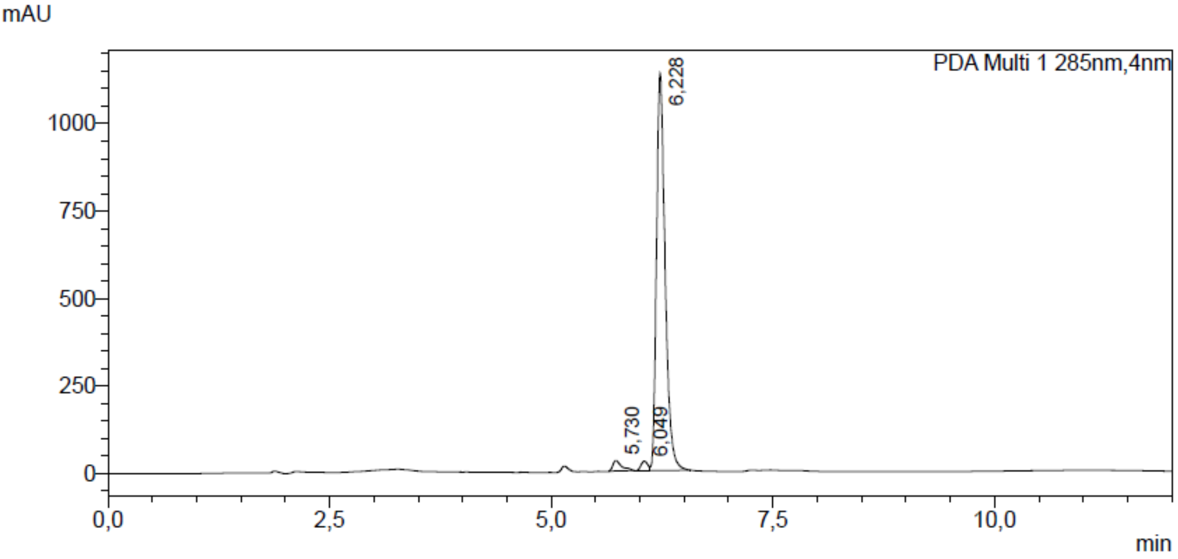

Peak Table

PDA Ch1 285nm

Peak#	Ret. Time	Area	Height	Area%
1	5,730	209385	30275	2,610
2	6,049	136398	26490	1,700
3	6,228	7676722	1132515	95,690
Total		8022505	1189281	100,000

MS Spectrum

Line#:1 R.Time:6,250(Scan#:376)
MassPeaks:514
Spectrum Mode:Single 6,250(376) Base Peak:733(7251052)
BG Mode:None Segment 1 - Event 2

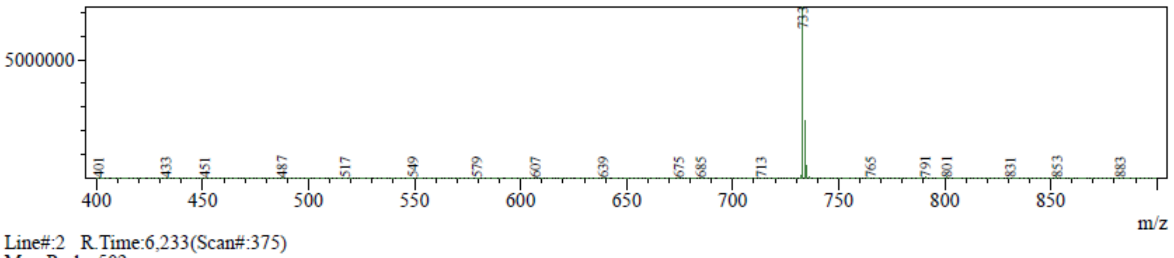

Line#:2 R.Time:6,233(Scan#:375)
MassPeaks:502
Spectrum Mode:Single 6,233(375) Base Peak:475(380314)
BG Mode:None Segment 1 - Event 1

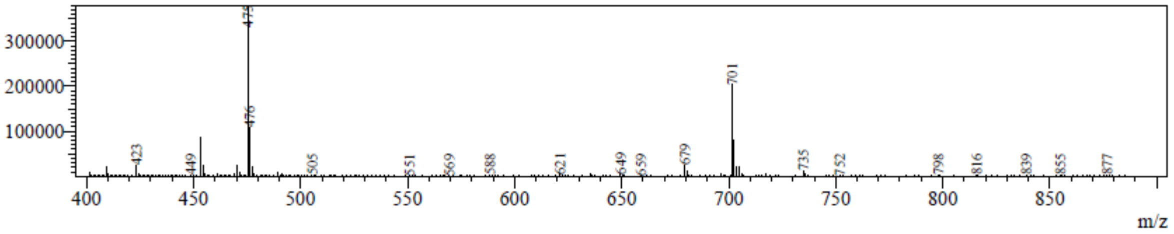

Figure S31. HPLC chromatogram and LC/MS spectra of **6,8-diiodosilychristin A (27)** Detected at 285 nm. Chromolith C18, 0.4 mL/min, 25 °C. Product peak 6.228 min, purity 96%.

<Chromatogram>

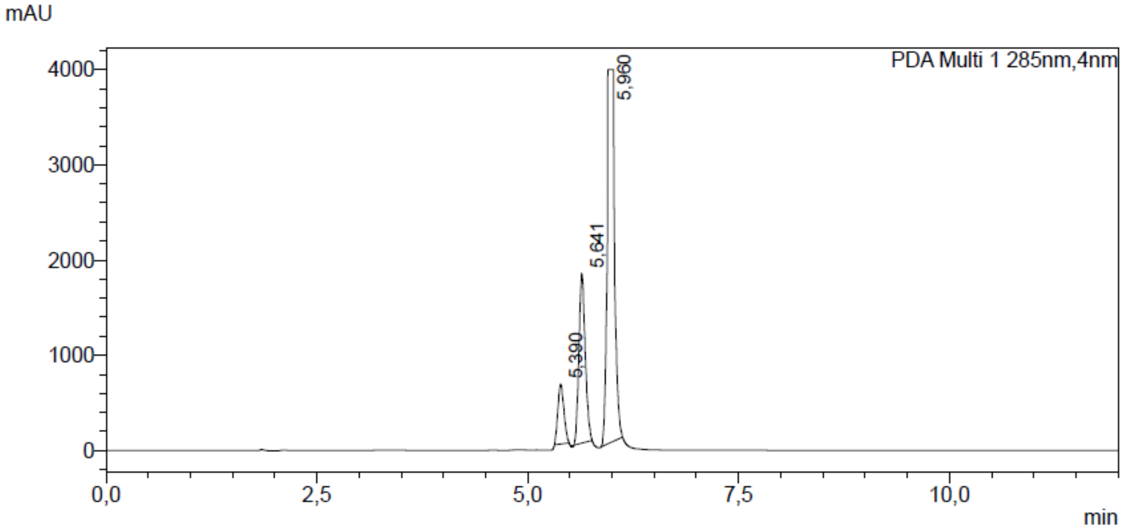

Peak Table

PDA Ch1 285nm

Peak#	Ret. Time	Area	Height	Area%
1	5,390	3114869	626078	8,519
2	5,641	9274287	1771639	25,365
3	5,960	24174833	3926659	66,117
Total		36563989	6324376	100,000

MS Spectrum

Line#:1 R.Time:6,017(Scan#:362)
MassPeaks:201
Spectrum Mode:Single 6,017(362) Base Peak:515(8807137)
BG Mode:None Segment 1 - Event 2

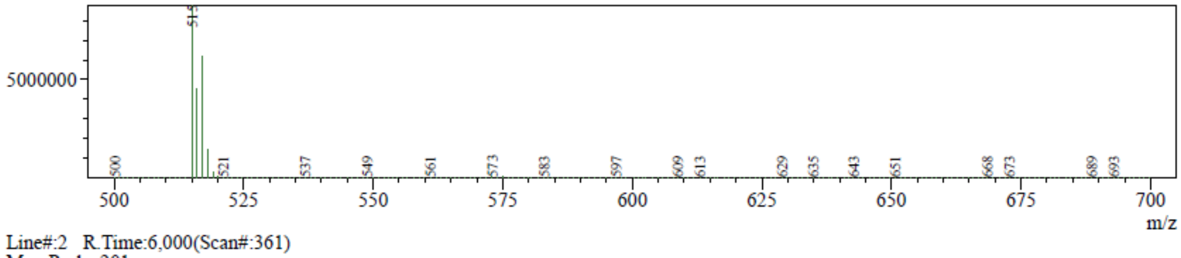

Line#:2 R.Time:6,000(Scan#:361)
MassPeaks:201
Spectrum Mode:Single 6,000(361) Base Peak:517(30929)
BG Mode:None Segment 1 - Event 1

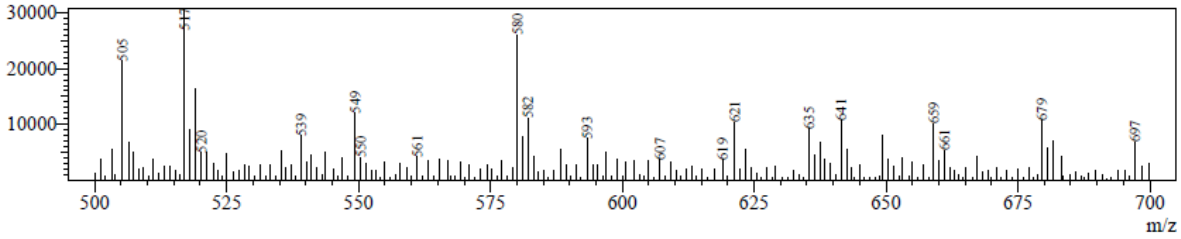

Figure S32. HPLC chromatogram and LC/MS spectra of **6-chlorosilybin B (28a) and 8-chlorosilybin B (28b).** Detected at 285 nm. Chromolith C18, 0.4 mL/min, 25 °C. The product was isolated as a mixture containing 8-chlorosilybin B (peak 5.641 min, 25%), 6-chlorosilybin B (peak 5.960 min, 66%), and silybin B (peak 5.390 min, 9%).

HRMS Analysis

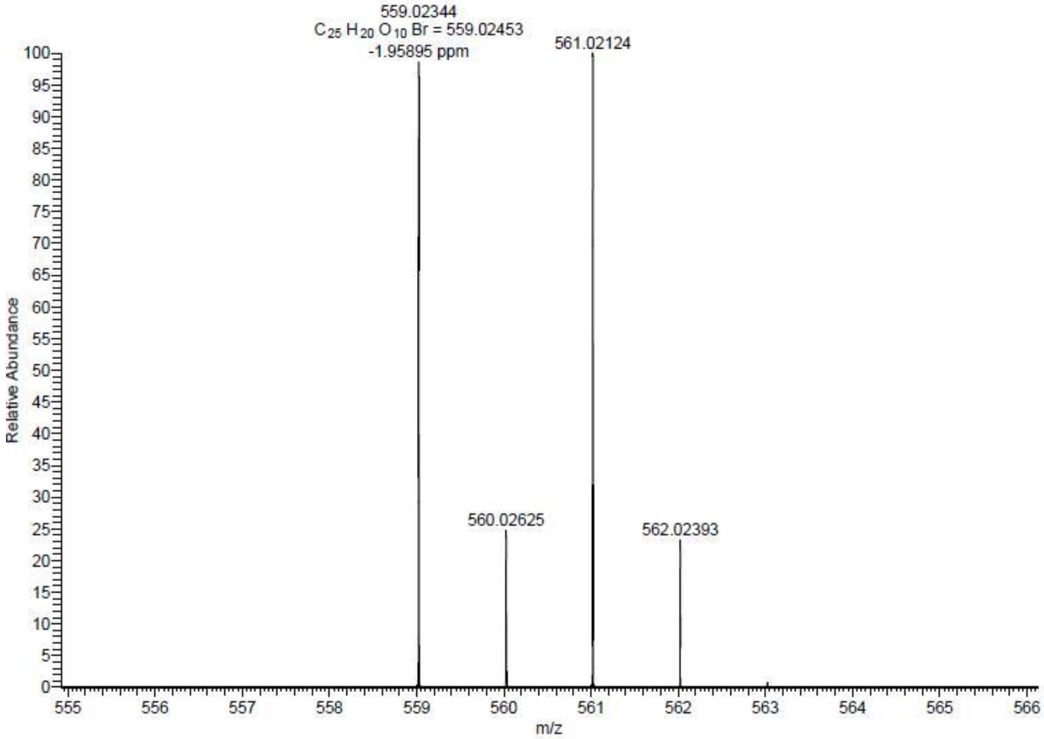

Figure S33. HRMS (ESI-) analysis of **8-bromosilybin A (19).** Calculated for C25H20O10^{79}Br *m/z* 559.02453, measured 559.02344 (-1.95895 ppm).

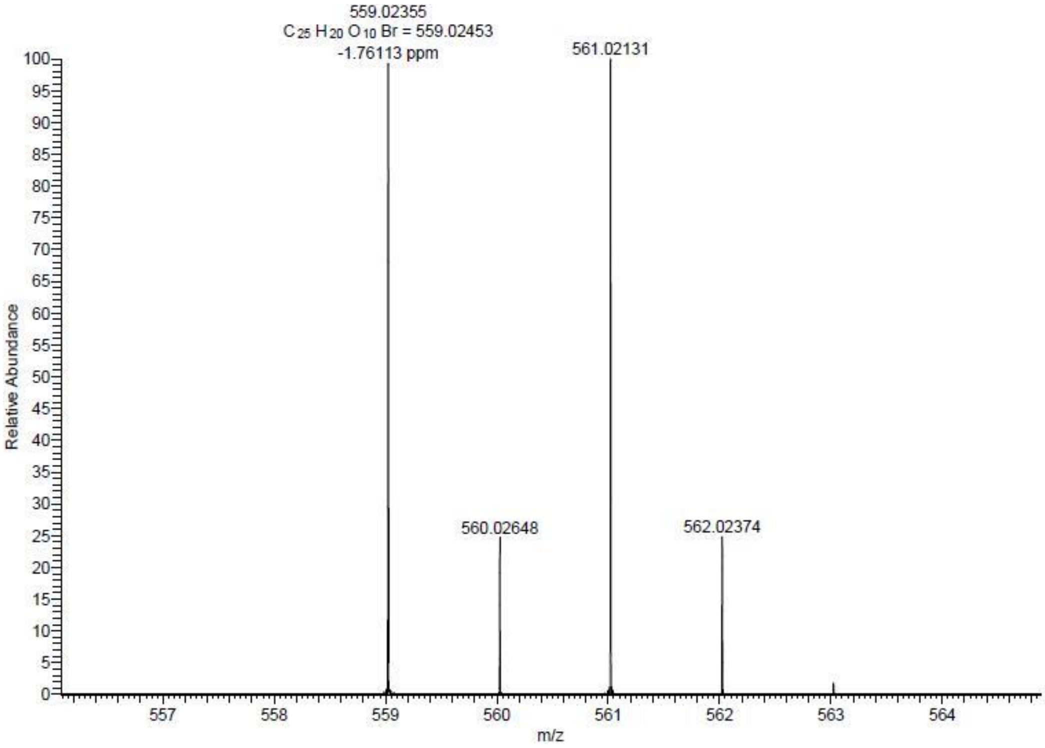

Figure S34. HRMS (ESI-) analysis of **8-bromosilybin B (20).** Calculated for C25H20O10^{79}Br *m/z* 559.02453, measured 559.02355 (-1.76113 ppm).

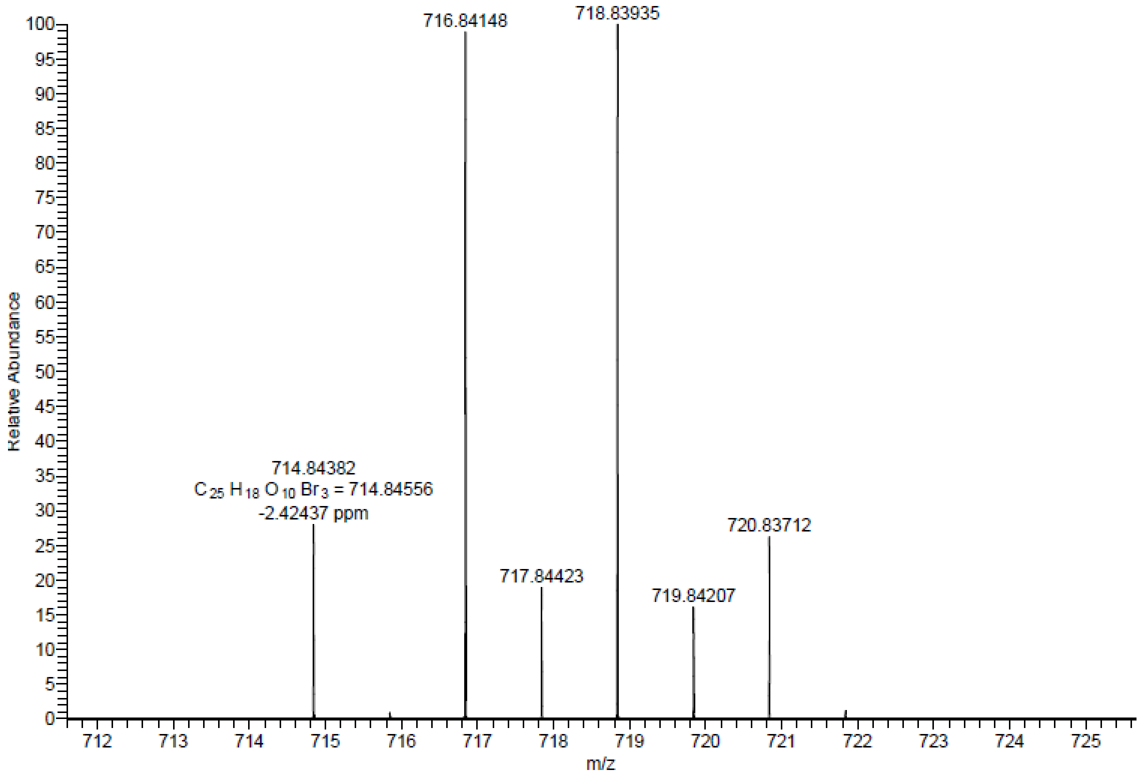

Figure S35. HRMS (ESI-) analysis of **6,8,21-tribromosilybin A (21).** Calculated for $C_{25}H_{18}O_{10}{}^{79}Br_3$ *m/z* 714.84556, measured 714.84382 (-2.42437 ppm).

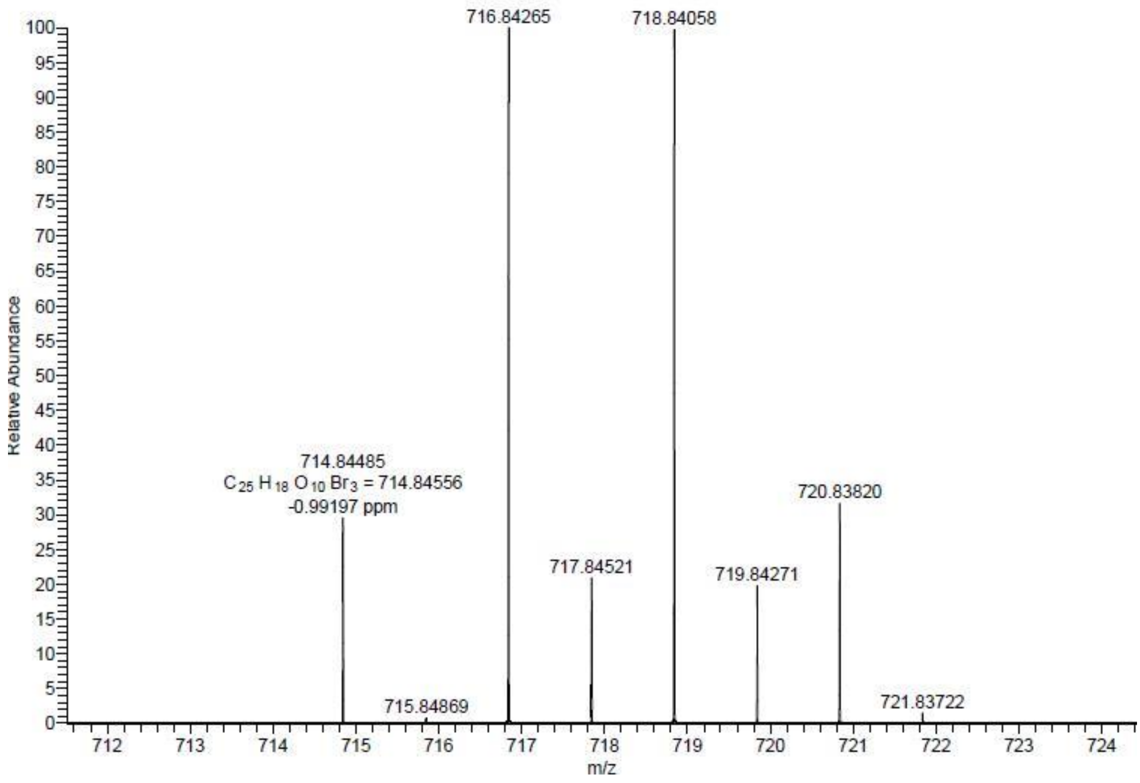

Figure S36. HRMS (ESI-) analysis of **6,8,21-tribromosilybin B (22).** $C_{25}H_{18}O_{10}{}^{79}Br_3$ *m/z* 714.84556, measured 714.84485 (-0.99197 ppm).

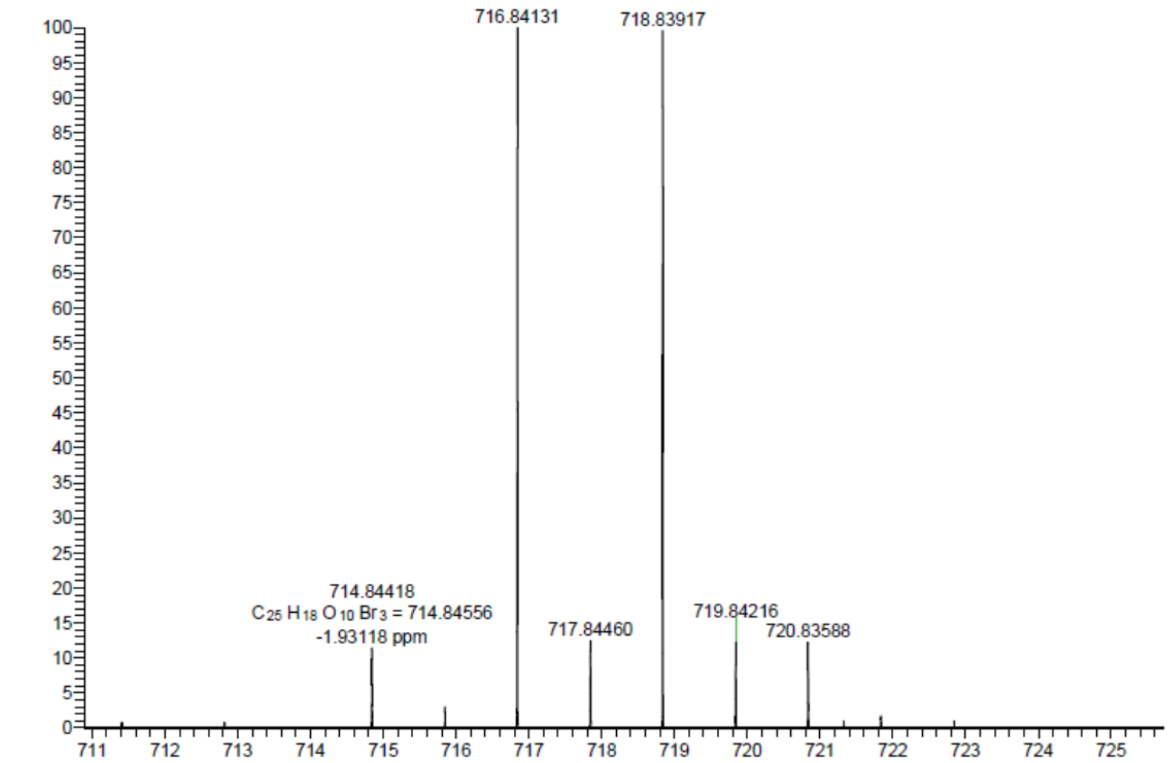

Figure S37. HRMS (ESI-) analysis of **6,8,20-tribromosilychristin A (23).** $C_{25}H_{18}O_{10}{}^{79}Br_3$ *m*/*z* 714.84556, measured 714.84418 (-1.93118 ppm).

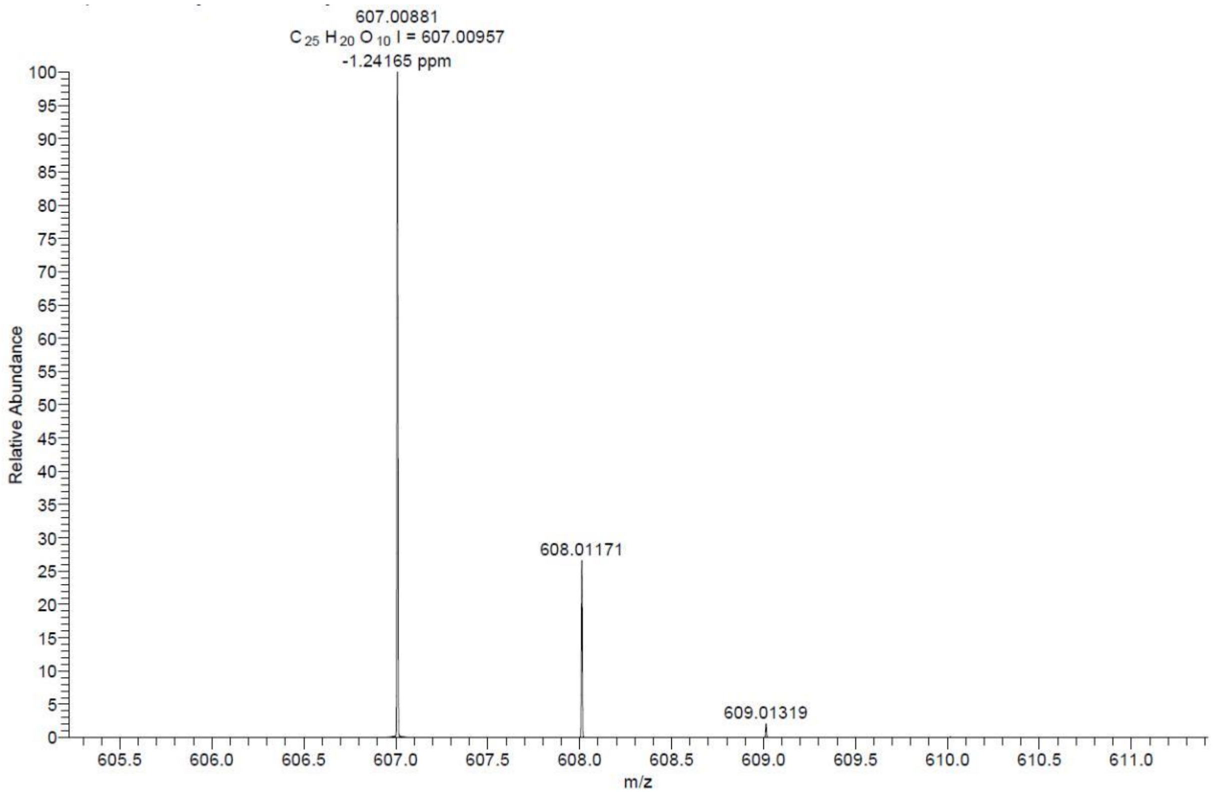

Figure S38. HRMS (ESI-) analysis of 8-iodosilybin (**24**). $C_{25}H_{20}O_{10}I$ *m*/*z* 607.00957, measured 607.00881 (-1.24165 ppm).

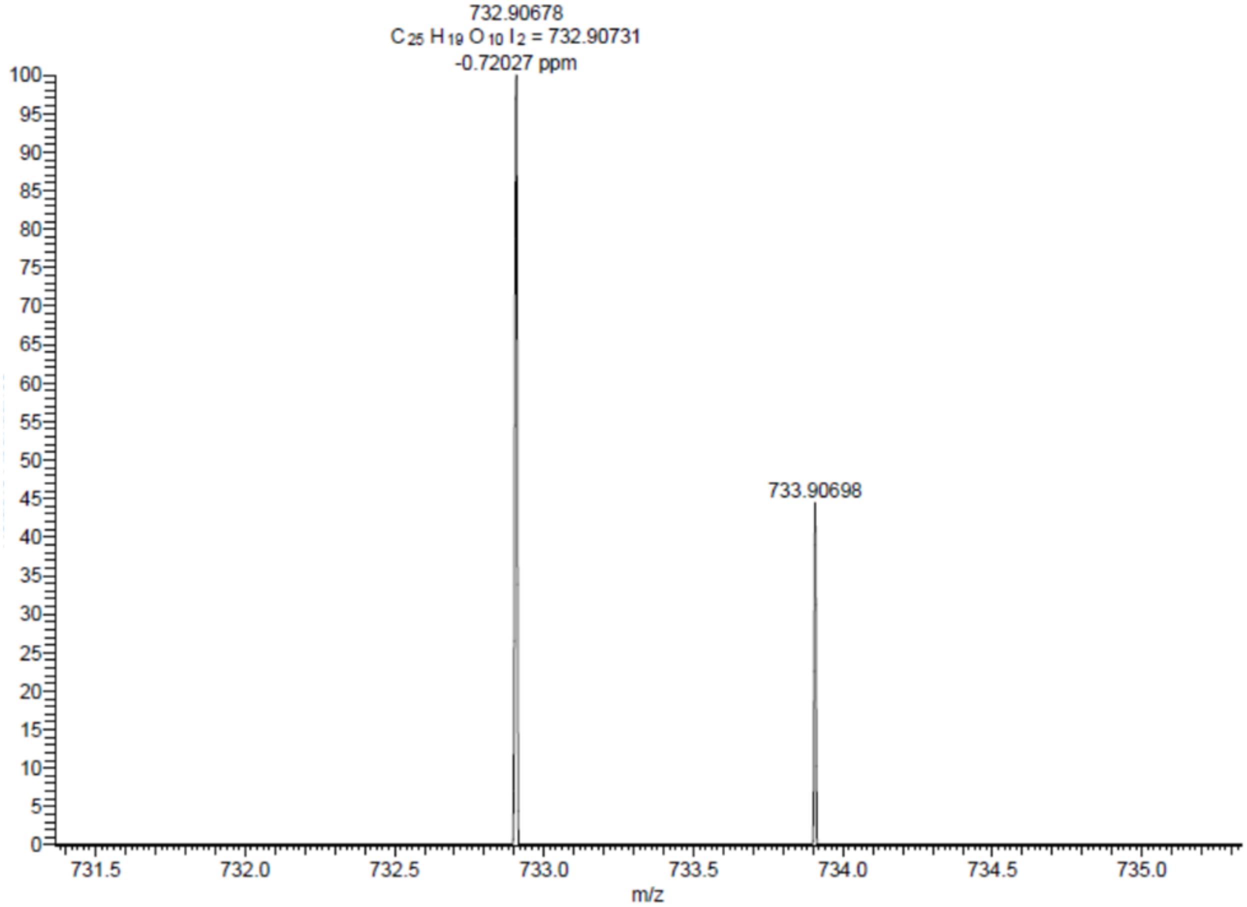

Figure S39. HRMS (ESI-) analysis of **6,8-diiodosilybin A (25).** $C_{25}H_{19}O_{10}I_2$ *m*/*z* 732.90731, measured 732.90678 (-0.72027 ppm).

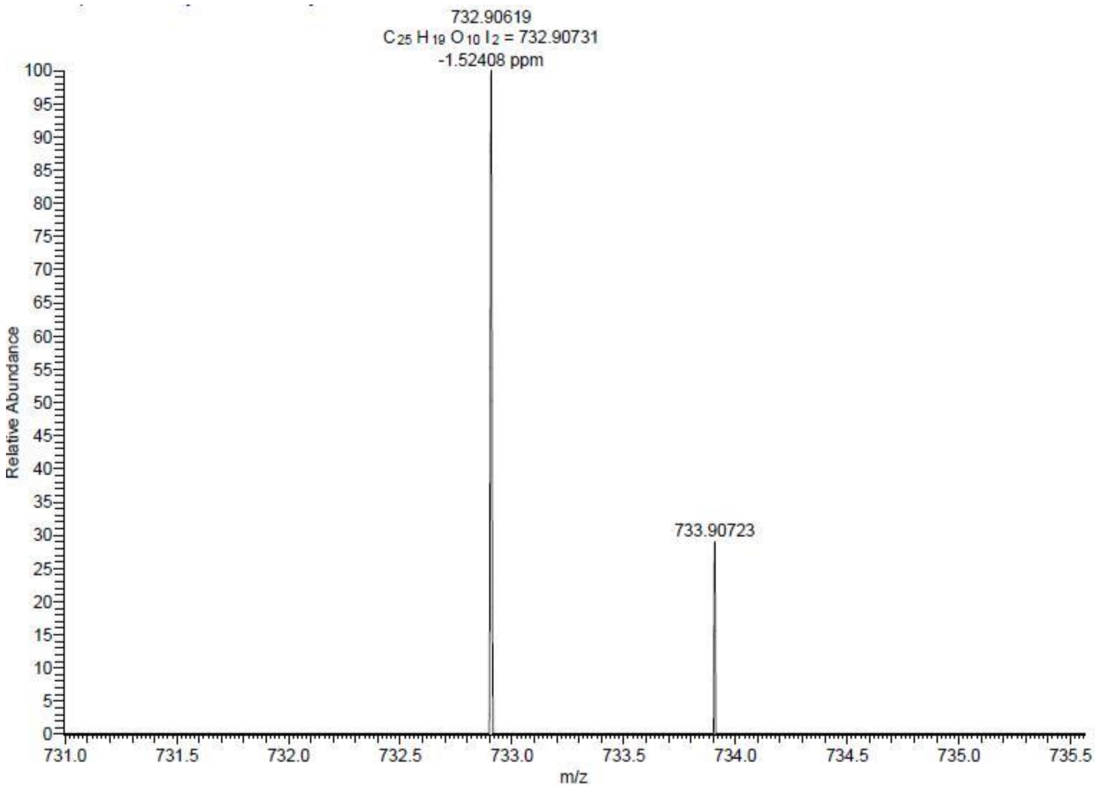

Figure S40. HRMS (ESI-) analysis of **6,8-diiodosilybin B (26).** $C_{25}H_{19}O_{10}I_2$ *m*/*z* 732.90731, measured 732.90619 (-1.52408 ppm).

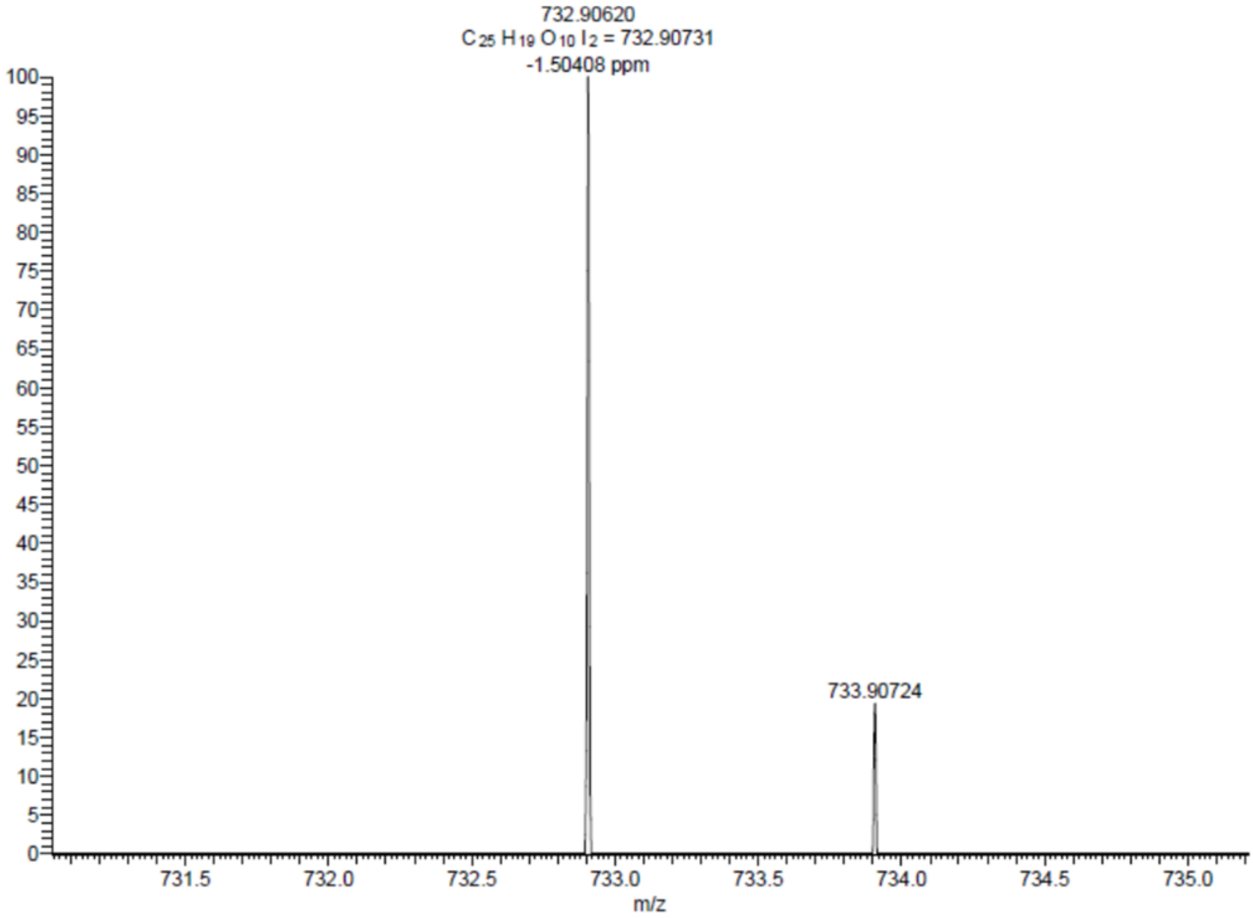

Figure S41. HRMS (ESI-) analysis of **6,8-diiodosilychristin A (27).** $C_{25}H_{19}O_{10}I_2$ *m/z* 732.90731, measured 732.90620 (-1.50408 ppm).

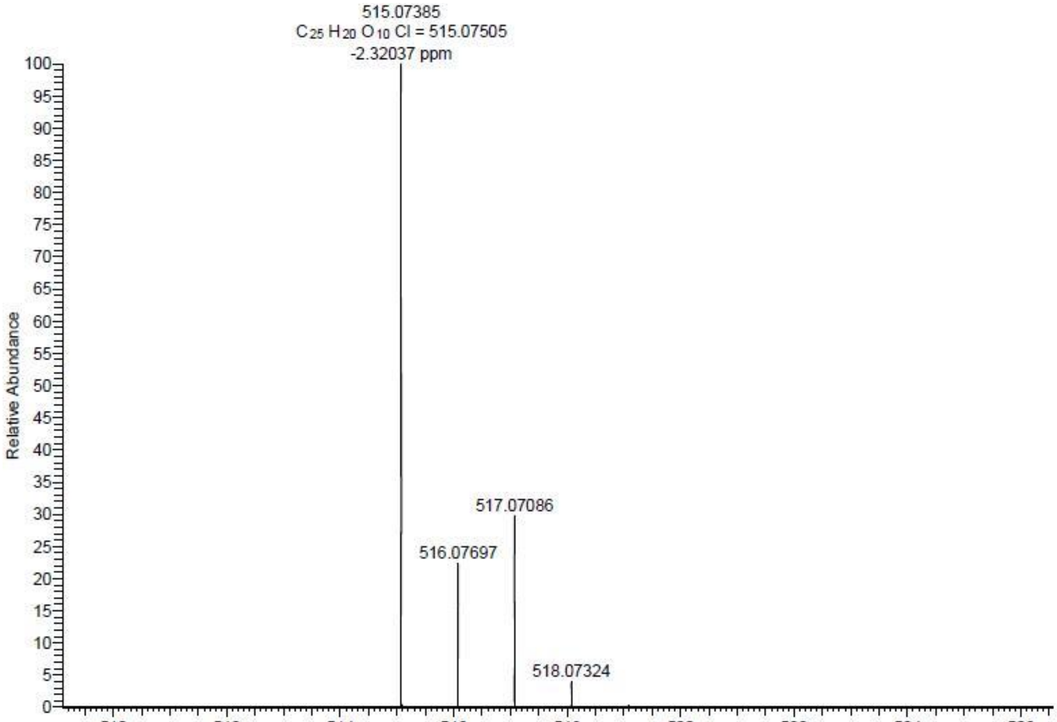

Figure S42. HRMS (ESI-) analysis of **6-chlorosilybin B (28a) and 8-chlorosilybin B (28b).** $C_{25}H_{20}O_{10}Cl$ *m/z* 515.07505, measured 515.07385 (-2.32037 ppm).
